# Large-scale phylogenetic analyses reveal multiple gains of actinorhizal nitrogen-fixing symbioses in angiosperms associated with climate change

**DOI:** 10.1038/srep14023

**Published:** 2015-09-10

**Authors:** Hong-Lei Li, Wei Wang, Peter E. Mortimer, Rui-Qi Li, De-Zhu Li, Kevin D. Hyde, Jian-Chu Xu, Douglas E. Soltis, Zhi-Duan Chen

**Affiliations:** 1 State Key Laboratory of Systematic and Evolutionary Botany, Institute of Botany, Chinese Academy of Sciences, Beijing 100093, China; 2 Shenzhen Key Laboratory of Southern Subtropical Plant Diversity, Fairy Lake Botanical Garden, Shenzhen, Chinese Academy of Sciences, Shenzhen 518004, Guangdong, China; 3 World Agroforestry Centre, East and Central Asia, Kunming 650201, China; 4 Key Laboratory for Plant Diversity and Biogeography of East Asia, Kunming Institute of Botany, Chinese Academy of Sciences, Kunming 650201, China; 5 Plant Germplasm and Genomics Center, Germplasm Bank of Wild Species, Kunming Institute of Botany, Chinese Academy of Sciences, Kunming, Yunnan 650201, China; 6 School of Science, Mae Fah Luang University, Chiang Rai 57100, Thailand; 7 Florida Museum of Natural History, University of Florida, Gainesville, FL 32611, USA

## Abstract

Nitrogen is fundamental to all life forms and is also one of the most limiting of nutrients for plant growth. Several clades of angiosperms have developed symbiotic relationships with actinorhizal bacteria that fix atmospheric nitrogen and increase access to this nutrient. However, the evolutionary patterns of actinorhizal nitrogen-fixing symbioses remain unclear to date. Furthermore the underlying environmental pressures that led to the gain of symbiotic actinorhizal nitrogen fixation have never been investigated. Here, we present the most comprehensive genus-level phylogenetic analysis of the nitrogen-fixing angiosperms based on three plastid loci. We found that actinorhizal nitrogen-fixing species are distributed in nine distinct lineages. By dating the branching events, we determined that seven actinorhizal nitrogen-fixing lineages originated during the Late Cretaceous, and two more emerged during the Eocene. We put forward a hypothesis that multiple gains of actinorhizal nitrogen-fixing symbioses in angiosperms may have been associated with increased global temperatures and high levels of atmospheric carbon dioxide during these two time periods, as well as the availability of open habitats with high light conditions. Our nearly complete genus-level time-tree for the nitrogen-fixing clade is a significant advance in understanding the evolutionary and ecological background of this important symbiosis between plants and bacteria.

Nitrogen is fundamental to all life forms and is a critical component of photosynthesis and amino acid production in plants. Nitrogen is also one of the most limiting of nutrients for plant growth^1^. One mechanism that has evolved in angiosperms in response to environments in which nitrogen is limiting is symbiosis with actinorhizal (*Frankia*)^2^,^3^ or rhizobial^4^,^5^ bacteria that fix atmospheric nitrogen (N_2_)^2^. Both actinorhizal and rhizobial bacteria convert atmospheric N_2_ into ammonium (NH_4_) in the process of nitrogen-fixation, which takes place in the root nodules, organs on the plant roots that house the nitrogen-fixing bacteria.

Despite the benefits to plants that are associated with nitrogen-fixing symbioses, only about 2.5% of angiosperm families are able to form these symbioses^6^. Of these families, 25 genera from Betulaceae, Casuarinaceae, Coriariaceae, Datiscaceae, Elaeagnaceae, Myricaceae, Rhamnaceae, and Rosaceae are actinorhizal (Table S1)^2^,^3^ and most of the 730 genera of Fabaceae (legumes) and *Parasponia* of Cannabaceae are rhizobial^4^,^5^.

Molecular phylogenetic analyses have revealed that families of nodulating plants, together with 18 families of non-nodulating plants, are confined to a single large nitrogen-fixing clade^6^,^7^,^8^,^9^. The close relationships among nitrogen-fixing plant families provide evidence of a single origin of the “predisposition” for root-nodule symbioses with the possibility of recurrent losses or recurrent gains of nitrogen-fixing symbioses in the nitrogen-fixing clade^6^. Based on a phylogeny of 3,467 angiosperm species, Werner *et al.*^10^ further confirmed a single origin of the precursor to symbioses with nitrogen-fixing bacteria (the predisposition for nitrogen fixation), followed by multiple gains of symbioses (see ref. 6). The eight actinorhizal nitrogen-fixing families are so diverse that they were considered distantly related in conventional taxonomic systems^11^,^12^. Subsequent analyses using improved phylogenetic trees suggested that within the actinorhizal nitrogen-fixing lineages there were a minimum of six gains with two subsequent losses for actinorhizal nitrogen-fixing symbioses^13^. A further four independent gains are hypothesized by comparing the morphology of nodules from 18 actinorhizal nitrogen-fixing genera^3^,^14^,^15^. Doyle^16^ has suggested that symbiotic nitrogen-fixing symbioses occurred at least eight times in actinorhizal nitrogen-fixing lineages. However, these numbers may not be accurate, as they are based on samples of only 13 of 25 genera with actinorhizal nitrogen-fixing symbioses. Although Werner *et al.*^10^ contributed greatly to our understanding of evolution of nitrogen-fixing symbioses, their study included only 438 of approximately 1300 genera in the nitrogen-fixing clade^17^, and 18 of the 25 plant genera with actinorhizal nitrogen-fixing symbioses. Despite tremendous progress in our understanding of the evolution of nitrogen-fixing symbioses in angiosperms, the patterns of actinorhizal nitrogen fixation through time remain unclear to date due to limitations in the sampling strategies of past studies and the absence of a comprehensive dated phylogenetic tree for the nitrogen-fixing clade of angiosperms^9^,^17^.

The oldest fossils of angiosperms from the nitrogen-fixing clade are of Myricaceae pollen, from the Cenomanian period of the Cretaceous, 97.5–91 Ma^18^. The earliest putative nodule fossil comes from mesofossil assemblages from the Santonian (~84 Ma) of the Cretaceous^19^. The ancestor of the nitrogen-fixing clade evolved in the early Cretaceous (ca. 92–110 Ma)^9^,^17^. This evidence suggests that plants and nitrogen-fixing bacteria first evolved a symbiotic relationship in the Late Cretaceous. Since the Cretaceous, the Earth’s environment has been characterized by climate change and major plate tectonic events that have shaped the evolution of angiosperms^20^,^21^. The response of the actinorhizal nitrogen-fixing plants to environmental fluctuations since the Late Cretaceous also has not been established.

Here, we constructed the most comprehensive phylogeny to date of genera of angiosperms with nitrogen-fixing symbioses, basing our analysis on three plastid loci. Using molecular dating techniques we estimated the ages of the actinorhizal nitrogen-fixing lineages and investigated potential environmental factors associated with the gain of actinorhizal nitrogen-fixing symbioses in angiosperms.

## Results

### Phylogeny of angiosperms with nitrogen-fixing symbiosis

**Figure 1:**
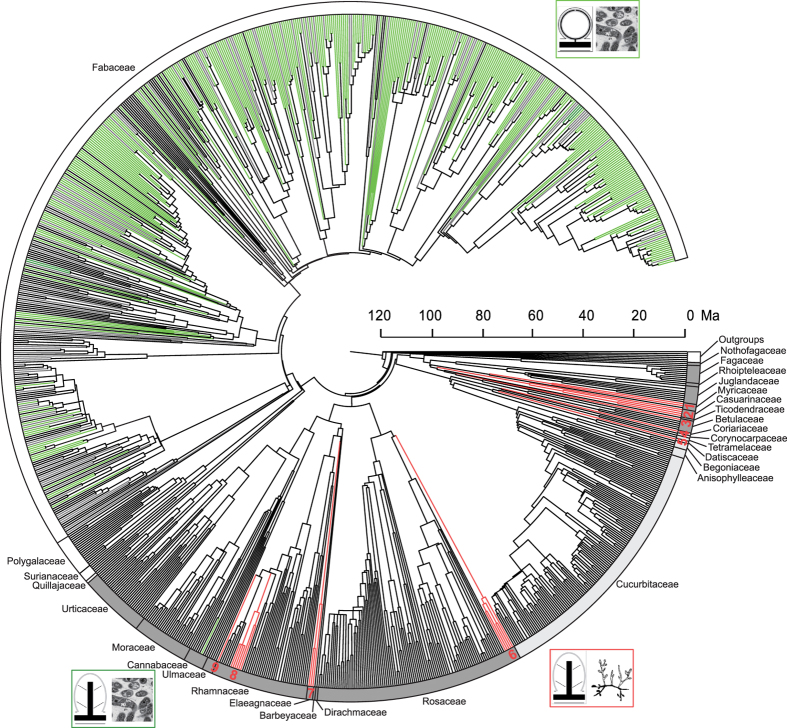
Chronogram of the nitrogen-fixing clade. Dating analysis was performed using PL method. Green indicates rhizobial lineages; red represents actinorhizal nitrogen-fixing lineages (numbered 1–9); grey represents unknown; black represents no nitrogen-fixing symbiosis. Green box indicates the nodule morphological characteristics of rhizobial nitrogen-fixing plants and their symbiotic partners, and red box indicates that of actinorhizal nitrogen-fixing plants and their symbiotic partners.

In general, the topologies and support values for the nitrogen-fixing clade inferred using maximum likelihood (ML) and Bayesian methods are congruent (Figs S1,S2). No major conflicts (nodes with bootstrap support value >50% and posterior probability >0.5) were detected. Twenty-four actinorhizal nitrogen-fixing genera are distributed in nine lineages (lineages 1–9; Fig. 1, S1, S2), and their likely sister groups based on our analyses are given in Table 1.

### Age estimation

Age estimates for the nitrogen-fixing clade from penalized likelihood (PL) analysis in r8s are shown in Fig. 1 and from the Bayesian relaxed clock (BRC) analysis in BEAST in Fig. S4. The 95% highest posterior density (HPD) of the Fabales crown age ranges from 104.0 (97.9–107.1) (PL) to 101.7 (91.6–110.6) (BRC) Ma, the Fagales from 104.1 (99.9–106.5) to 104.9 (100.0–110.1) Ma, the Cucurbitales from 86.2 (78.8–91.0) to 86.9 (71.3–103.3) Ma, and the Rosales from 106.1 (103.6–108.5) to 106.5 (100.2–112.6) Ma.

**Figure 2:**
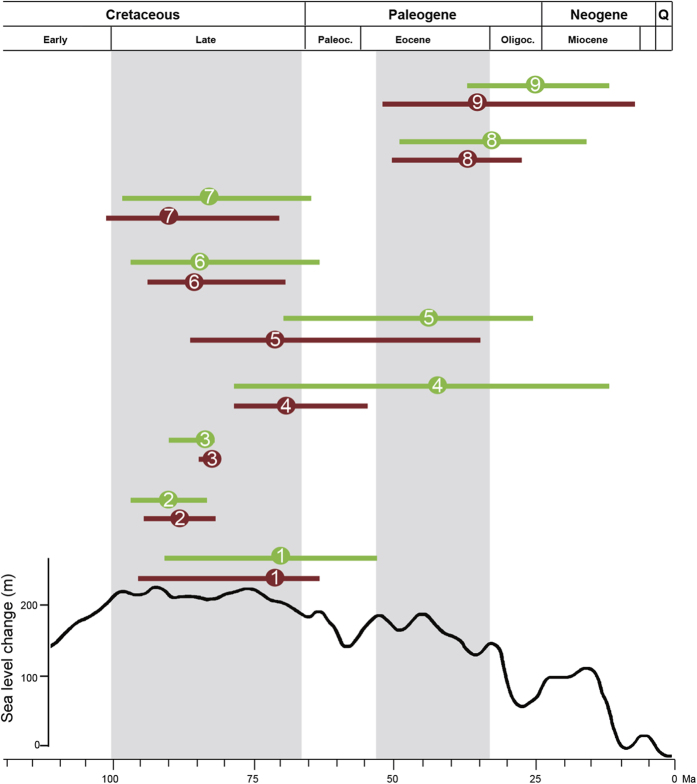
The 95% confidence interval of the divergence time of the actinorhizal nitrogen-fixing lineages. Red indicates results from r8s. Green indicates results from BEAST. All numbers are in accordance with results shown in Fig. 1.

Estimated stem ages for the actinorhizal nitrogen-fixing lineages provided by PL and BRC methods are highly consistent, as shown by the 95% HPD in Fig. 2 and Table S2. Our estimates for the origin of stem group of Elaeagnaceae (lineage 7) from the PL and BRC analyses range from 91.1 (71.4–101.8) to 82.8 (64.5–97.8) Ma; Casuarinaceae (lineage 2) 90.0 (84.3–93.8) to 92.5 (88.4–97.0) Ma, Dryadoideae (lineage 6) 87.6 (68.6–93.7) to 86.1 (63.3–96.1) Ma, *Alnus* (lineage 3) 83.0 (83.0–84.3) to 85.9 (84.1–88.6) Ma, *Myrica* + *Comptonia* (lineage 1) 71.0 (61.7–95.7) to 69.8 (55.1–88.9) Ma, *Datisca* (lineage 5) 66.4 (30.6–85.8) to 42.7 (22.4–66.2) Ma, *Coriaria* (lineage 4) 65.7 (56.0–79.5) to 41.4 (10.2–79.5) Ma, Colletieae (lineage 8) 36.8 (25.2–50.0) to 31.9 (18.9–47.5) Ma, and *Ceanothus* (lineage 9) 34.1 (6.9–52.6) 24.0 to (12.2–37.8) Ma.

## Discussion

Our analyses, which are based on an extensive generic sampling of nitrogen-fixing angiosperms, strongly support the monophyly of the nitrogen-fixing clade. The relationships among the Cucurbitales, Fabales, Fagales, and Rosales, and the branching of the families within each order (Fig. S1, S2), are also in accordance with the results of previous studies^7^,^8^,^9^,^13^. The actinorhizal nitrogen-fixing lineages are distributed in Rosales, Cucurbitales, and Fagales, whereas the rhizobial nitrogen-fixing lineages are limited to Fabaceae and *Parasponia* of Cannabaceae (Fig. 1). The nodule structure of actinorhizal nodules markedly differs from that of legume nodules (resembling a root vs. a shoot; Fig. 1)^2^. The infection threads of *Parasponia* are similar to those of some early diverging legumes e.g., *Andira* and *Chamaecrista*^2^.

PL and BRC methods resulted in similar time estimates for the major lineages of the nitrogen-fixing clade (Fig. 1, S4). The crown ages of the four orders are in accordance with the results of previous studies (Table S2) and the two methods generated highly consistent results for the ages of the nine actinorhizal nitrogen-fixing lineages except lineages 4 and 5 (Fig. 2). The BRC analysis indicates that lineages 4 (Coriariaceae) and 5 (Datiscaceae) occurred during the Paleogene, whereas the PL analysis results in a late Cretaceous time for each. Yokoyama *et al.*^22^, based on molecular-based estimates, suggested a late Cretaceous origin of Coriariaceae, and the extant Datiscaceae also date to the late Cretaceous^23^,^24^. The 95% HPDs resulting from our two analyses also overlapped for the lineages 4 and 5. Thus, we consider that Coriariaceae and Datiscaceae originated in the late Cretaceous. The stem ages of lineages 1, 2, and 3 fall in the late Cretaceous, in accordance with the results of Xiang *et al.*^25^, and the stem age of lineage 6 agrees with the results of Chin *et al.*^26^. The stem age of lineage 7 agrees with a number of analyses (e.g., Bell *et al.*^17^; Magallón *et al.*^27^). The stem ages of lineages 8 and 9 agree with the results of Richardson *et al.*^28^. A comparison of time estimates of actinorhizal nitrogen-fixing lineages is shown in Table S2. The congruence among these studies enhances the reliability of our divergence date estimates for the origin of the actinorhizal nitrogen-fixing lineages.

According to our analyses, the oldest actinorhizal nitrogen-fixing lineage diverged from its extant sister in the Late Cretaceous (PL: 71.4–101.8 Ma; BRC: 64.5–97.8 Ma), and the earliest nodule fossil dates to the Santonian (83.6–86.3 Ma)^19^. These results support the hypothesis that the symbiosis between angiosperms and actinorhizal nitrogen-fixing bacteria may have originated in the Late Cretaceous^16^. The inferred credibility intervals of the estimated times of divergence indicate that the rise of extant actinorhizal nitrogen-fixing lineages occurred during two periods. The first appearance of actinorhizal nitrogen-fixing lineages was during the Late Cretaceous, when seven extant actinorhizal nitrogen-fixing lineages originated (lineages 1–7), and the second appearance was during the Eocene (lineages 8–9).

During the late Cretaceous, the global climate was relatively warm with a surface temperature 3–5 °C warmer than today^29^,^30^,^31^,^32^,^33^, and atmospheric CO_2_ levels were relatively high (ca. 2–4 times present atmospheric level)^32^,^33^. The temperature dropped about 3 °C, and atmospheric CO_2_ levels fell to 1.5 times present atmospheric level in the Paleocene (66.0–56.0 Ma)^29^,^30^,^31^,^32^,^33^. During the Eocene, the global surface temperature increased 3–4 °C^29^,^30^,^31^,^32^,^33^, and atmospheric CO_2_ levels rose to about 2.5–3 times the present atmospheric level^32^,^33^. Previous studies have indicated that high temperature and CO_2_ levels are vital to the nitrogen-fixing process^34^,^35^. In *Myrica gale*, nodule vesicles are rare, and nitrogenase activity is undetectable in the perennial actinorhizal nodules during cold periods. Some research has suggested that a doubling of CO_2_ content promoted increased nitrogen as activity in alders (*Alnus glutinosa*)^35^, plants having a nitrogen fixing symbiosis. This suggests that actinorhizal nitrogen-fixing symbioses in plants could be associated with or perhaps selected for by an increase in atmospheric CO_2_. It is noteworthy that our dated tree indicates that the evolution of nitrogen-fixing symbiosis in members of the nitrogen-fixing clade occurred at roughly the time of high temperature and high atmospheric CO_2_ levels^29^,^30^,^31^,^32^,^33^. This hypothesis, based on the observed correlation of the evolution of actinorhizal nitrogen-fixing symbioses and elevated temperatures and increased CO_2_ levels, requires rigorous testing via future research. The gains of symbiotic actinorhizal nitrogen fixation would also likely have been strongly influenced by the environment in which the plant groups occurred. Actinorhizal nitrogen-fixing plants share the tendency to grow in soils where nitrogen availability is low (Table S1). The overwhelming majority of actinorhizal nitrogen-fixing plants are trees, shrubs, or lianas, which are often early successional pioneer species (Table S1). Historical records that fossilized nodules belonging to an actinorhizal nitrogen-fixing plant species in Elaeagnaceae were found in a typical early successional habitat^36^ support contemporary observations. A few actinorhizal nitrogen-fixing plants are trees (e.g., *Alnus* and *Casuarina*); however, such species are often fast-growing and short-lived and tend not to form stable, closed communities. For example, *Alnus* has the tendency to grow in open places and play an important role in early successional habitats^37^,^38^,^39^. Only *Datisca* is herbaceous and inhabits rocky places along streambeds and in desert regions (Table S1)^40^. As pioneers, these actinorhizal nitrogen-fixing plants can take advantage of the ecological niche presented by low nitrogen availability. In addition, the habitats of actinorhizal nitrogen-fixing plants are often open and have good light conditions. High light conditions can supply energy for the nitrogen-fixing process by allowing for higher rates of photosynthesis, leading to an increased production of photosynthate^41^,^42^. Interestingly, the green leaves of some species of alder (e.g., *Alnus glutinosa*) are retained longer than those of other temperate deciduous trees that are unable to fix nitrogen^43^. This longer period of retaining leaves in alders prolongs the production of photosynthate for these plants in early autumn^44^.

## Conclusions

By assembling a large three-locus dataset, we produced, to our knowledge, the most comprehensive genus-level phylogeny for the nitrogen-fixing clade to date. Our phylogenetic tree with dense sampling of nitrogen-fixing angiosperms, including 24 of the 25 actinorhizal nitrogen-fixing genera and the majority of known rhizobial nitrogen-fixing genera so far, should be beneficial to research on the nitrogen-fixing symbioses. This phylogeny will be a useful tool for comparative biology of diverse features, including morphological and ecological diversification, physiological function, and genome evolution. Our analyses confirmed that actinorhizal nitrogen-fixing species are distributed in at least nine distinct lineages. Based on our divergence time estimates, we show that there were multiple evolutionary pulses of the gains of nitrogen-fixing symbioses through time, seven actinorhizal nitrogen-fixing lineages evolved during the Late Cretaceous, while during the Eocene, two more actinorhizal nitrogen-fixing lineages emerged. Furthermore, based on our divergence time estimates, we hypothesize that the gain of actinorhizal nitrogen-fixing symbioses in angiosperms may have coincided with important climatic changes, such as high CO_2_, high global surface temperatures, and the availability of open habitats with high light conditions.

The hypothesis remains to be further tested by studying fossils, comparative physiology, and the genetic control of features, such as nodules, that enable nitrogen-fixing symbioses.

## Materials and Methods

### Taxon sampling, molecular markers, and DNA alignment

A total of 1,002 genera representing all families of the nitrogen-fixing clade and approximately 80% of all genera in the clade^45^ were included in our analysis. Our sampling scheme included 24 of the 25 known actinorhizal nitrogen-fixing plant genera (the only genus missing is *Talguenea*), *Parasponia* (a non-legume that hosts rhizobial nodules), and 578 of ca. 730 genera in Fabaceae. Six species of eudicots outside of the nitrogen-fixing clade were selected as outgroups^9^. Three genetic markers from the plastid genome, *rbcL*, *matK*, and *trnL-F*, were used in the phylogenetic analysis. Sequence data for all taxa were obtained from GenBank. For each species, sequences generated from the same specimens were selected. For each region, if multiple sequences were available, the longest sequence was used, or, when sequences were the same length, one was randomly selected. Species names and GenBank accession numbers are listed in Table S3.

The *rbcL* sequences were aligned directly using MUSCLE with the default set at the high-accuracy parameter^46^, and alignment was checked and adjusted manually with BioEdit v5.0.9^47^. For the fast-evolving *matK* and *trnL-F* regions, a two-step strategy was employed for each region to generate a high-quality alignment. The first step involved dividing sequences into clusters at the family level according to sequence length and taxonomic unit. Each sequence cluster was aligned by MUSCLE under default high-accuracy parameters and then manually adjusted. In the second step, sequence clusters were aligned with one another by employing the profile-profile alignment algorithm in MUSCLE. In the final step, manual adjustments were made to the complete alignment.

### Phylogenetic analysis

To detect sequences of questionable quality, we first conducted a phylogenetic analysis for each of the three individual data sets using ML in RAxML v7.6.6^48^. The GTR + I + Γ model was selected as the best-fit model, as determined by ModelTest v3.7^49^. The sequences of those species that were placed in problematic positions compared to other well-supported analyses (e.g., Cardoso *et al.*^50^; Schaefer & Renner^51^) were removed from the final analyses. Phylogenetic analysis for the combined data set was performed using ML in RAxML v7.6.6^48^. Each marker sequence of the concatenated alignment was assigned a separate GTR + I + Γ model. We ran 1,000 non-parametric bootstrap replicates to assess uncertainty in the topology and branch length estimates. The program was run on the CIPRES network (http://www.phylo.org/).

### Molecular dating

To date the branching events within the nitrogen-fixing clade, we used a penalized likelihood method^52^ based on the complete data set of 1,008 terminals. We used the phylogeny and branch lengths estimated from the ML analysis as inputs into r8s v1.7^52^. We selected 27 fossil taxa as minimum calibration points; these taxa could be confidently assigned to clades and nodes represented in our data set (Table S4). The root age was fixed at 125 Ma based on the earliest reported date of eudicot pollen occurrence^53^. To determine the variation around the ages of each node, we sampled 100 bootstrap trees, generated from the ML analysis, and approximated the 95% credibility interval for each calibration using the profile command in r8s.

For the purpose of comparison, we also estimated divergence times using a Bayesian relaxed clock method^54^. Due to the logistical constraints of using the BRC methodology as implemented in BEAST (a full analysis required more than 1 year to perform), we reduced our combined data set to a smaller subset, containing 232 taxa, on the basis of three criteria: (i) sampling possible sisters of nitrogen-fixing taxa, (ii) for large clades, sampling the sister lineage to the remainder of the clade, and (iii) using taxa with the longest sequence regions. Divergence times were obtained using the program BEAST v1.7.5^54^ and the reduced data set. Parameters were set as follows: GTR + I + Γ model, estimated base frequencies, Γ categories 4, uncorrelated lognormal relaxed clock model, and the Yule speciation process. Eleven fossils were treated with the minimum age constraints within the lognormal distribution (see Table S5). Additionally, the fossil age of eudicot pollen^53^ was used to constrain the root age, with a normal distribution, at a mean age of 125 Ma, and a standard deviation of one. Markov Chain Monte Carlo analyses were run for 10 billion generations. The effective sample size was ensured to be over 200, as detected by Tracer v.1.5 (http://beast.bio.ed.ac.uk/software/tracer/). The maximum clade credibility tree was reconstructed using TreeAnnotator 1.5.4 that are part of the BEAST package.

## Additional Information

**How to cite this article**: Li, H.-L. *et al.* Large-scale phylogenetic analyses reveal multiple gains of actinorhizal nitrogen-fixing symbioses in angiosperms associated with climate change. *Sci. Rep.* **5**, 14023; doi: 10.1038/srep14023 (2015).

## Supplementary Material

Supplementary Information

## Figures and Tables

**Table 1:** Actinorhizal nitrogen-fixing lineages identified in this study, with recommendations for comparative studies of closely related non nitrogen-fixing taxa.

**Actinorhizal lineages**	**Clear sister for comparative work?**	**Bootstrap values**
1–*Myrica* + *Comptonia*	*Canacomyrica*	100%
2–Casuarinaceae	Ticodendraceae + Betulaceae	100%
3–*Alnus*	Remaining Betulaceae	100%
4–*Coriaria*	*Corynocarpus*	100%
5–*Datisca*	Tetramelaceae	<50%
6–Dryadoideae	Rosoideae	70%
7–Elaeagnaceae	*Barbeya*	<50%
8–Colletieae	*Granitites* + *Alphitonia*	<50%
9–*Ceanothus*	Pomaderreae	63%

The lineage numbers correspond to those in Fig. 1.
