# Supplementary material for: Large-scale phylogenetic analyses reveal multiple gains of actinorhizal nitrogen-fixing symbioses in angiosperms associated with climate change
Source: Sci Rep. 2015 Sep 10;5:14023. doi: 10.1038/srep14023 (PMC4650596; doi:10.1038/srep14023)
Supplement: Supplementary Information [file srep14023-s1.pdf]

Supplementary Information

Large-scale phylogenetic analyses reveal multiple gains of actinorhizal nitrogen-fixing symbioses in angiosperms associated with climate change

Hong-Lei Li[1,2,#], Wei Wang[1,#], Peter E. Mortimer[3,4], Rui-Qi Li[1], De-Zhu Li[4,5], Kevin D. Hyde[3,4,6], Jian-Chu Xu[3,4] , Douglas E. Soltis[7] & Zhi-Duan Chen[1]

[1]State Key Laboratory of Systematic and Evolutionary Botany, Institute of Botany, Chinese Academy of Sciences, Beijing 100093, China, [2]Shenzhen Key Laboratory of Southern Subtropical Plant Diversity, Fairy Lake Botanical Garden, Shenzhen, Chinese Academy of Sciences, Shenzhen 518004, Guangdong, China, [3]World Agroforestry Centre, East and Central Asia, Kunming 650201, China, [4]Key Laboratory for Plant Diversity and Biogeography of East Asia, Kunming Institute of Botany, Chinese Academy of Sciences, Kunming 650201, China, [5]Plant Germplasm and Genomics Center, Germplasm Bank of Wild Species, Kunming Institute of Botany, Chinese Academy of Sciences, Kunming, Yunnan 650201, China, [6]School of Science, Mae Fah Luang University, Chiang Rai 57100, Thailand, [7]Florida Museum of Natural History, University of Florida, Gainesville, FL 32611, USA.

[#] H.-L.L. and W.W. contributed equally to this work.

Correspondence and requests for materials should be addressed to Z.-D.C. (zhiduan@ibcas.ac.cn)

Table of Contents

Figure S1 | 1008-species tree from maximum likelihood analysis.

Figure S2 | The 231-taxa tree from Bayesian inference (BI).

Figure S3 | Chronogram of the nitrogen-fixing clade by Bayesian method.

Table S1 | Actinorhizal nitrogen-fixing plant taxa.

Table S2 | The age (million years ago, Ma) and the 95% highest posterior density (HPD) estimated for the stem of the nitrogen-fixing lineages.

Table S3 | List of taxa used in this study.

Table S4 | Minimal age constraints used in PL analysis.

Table S5 | Fossil constraints used in BRC analysis.

Supplementary Figure Legends

Figure S1 | 1008-species tree from maximum likelihood analysis. Numbers at nodes are BS proportions greater than 50%.

Figure S2 | The 231-taxa tree from Bayesian inference (BI). Numbers at nodes are PP≥0.5.

Figure S3 | Chronogram of the nitrogen-fixing clade by Bayesian method based on three combined markers.

Table S1 | Actinorhizal nitrogen-fixing plant taxa, distribution, habit, species numbers and habitat.

Genus	Distribution	Habit	Number of total species	Habitat
Adolphia	N America	shrubs	1	rocky places, steppes, river deltas, valley slopes and coasts
Allocasuarina	Australia	trees	59	soils markedly deficient in nutrients
Alnus	Europe, Asia, N. America, Andes Mts.	shrubs, trees	47	rocky places, sand, coasts and stream banks
Casuarina	Tropical Asia, Australia, S-W Pacific	shrubs, trees	18	stream banks
Ceanothus	N America	shrubs, trees	55	rocky places and coasts
Cercocarpus	Mexico, S-W United States	shrubs, trees	20	rocky places
Ceuthostoma	Oceania	shrubs, trees	2	rocky places, sand and stream banks
Chamaebatia	Sierra Nevada Mts.	shrubs	2	rocky places
Colletia	S America	shrubs	17	valley slopes and roadside
Comptonia	N America	shrubs	1	rocky places and sand
Coriaria	Mediterranean, Asia, New Zealand, N America	shrubs, subshrubs	16	river beds and road cuts
Cowania	Mexico, S-W United States	shrubs	25	rocky places
Datisca	Europe, Asia, N America	herbs	2	rocky places, stream beds and deserts
Discaria	S America, Australia, New Zealand	shrubs, trees	10	rocky places and sand
Dryas	Arctic	shrubs	3	rocky places and sand
Elaeagnus	Europe, Asia, N America	shrubs, trees, lianas	45	rocky places, steppes, river deltas, coastals and rain foreast
Gymnostoma	Australia, New Caledonia, Sumatra	shrubs, trees	18	rocky places, sand and stream banks
Hippophae	Europe, Asia	shrubs	3	rocky places, river deltas, valley slopes and coasts
Kentrothamnus	S America	shrubs	1	rocky places
Myrica	All continents except Australia	shrubs, trees	60	rocky places, coasts, bogs, sand and stream banks
Parasponia	Melanesia, Polynesia	shrubs, trees	5	open secondary forest
Purshia	W-N America	shrubs	4	rocky places
Retanilla	S America	shrubs	4	rocky places, coasts
Shepherdia	N America	shrubs	3	rocky places, steppes, river deltas, valley slopes and coasts
Trevoa	S America	shrubs	6	coasts

Table S2. The age (million years ago, Ma) and the 95% highest posterior density (HPD) estimated for the crown of the orders and the stem of the actinorhizal nitrogen fixers(1-9). The node numbers correspond to those in Fig. 1. * indicates crown ages.

lineage names	Age (LHPD-UPHD)		Comparison with previous analyses
	PL	BRC	
1-*Myrica+Comptonia*	71.0 (61.7–95.7)	69.8 (55.1–88.9)	69.7 (60.4–81.7)[1]
2-Casuarinaceae	90.0 (84.3–93.8)	92.5 (88.4–97.0)	82.8 (74.7–88.6)[1]
3-*Alnus*	83.0 (83.0–84.3)	85.9 (84.1–88.6)	59.6 (46.8–66.0)[1]
4-*Coriaria*	65.7 (56.0–79.5)	41.4 (10.2–79.5)	>63.1 ±14.7[*,2]
5-*Datisca*	66.4 (30.6–85.8)	42.7 (22.4–66.2)	>68[*,3]
6-Dryadoideae	87.6 (68.6–93.7)	86.1 (63.3–96.1)	>63.1(44.7–83.2)[*,4]
7-Elaeagnaceae	91.1 (71.4–101.8)	82.8 (64.5–97.8)	62 (52–72)[5]
8-Colletieae	36.8 (25.2–50.0)	31.9 (18.9–47.5)	32[6]
9-*Ceanothus*	34.1 (6.9–52.6)	24.0 (12.2–37.8)	34[6]
Fabales	104.0 (97.9–107.1)*	101.7 (91.6-110.6)*	98.7 (88.1-108.4)[*,7]
Rosales	106.1 (103.6–108.5)*	106.5 (100.2-112.6)*	99.0 (89-106.9)[*,7]
Fagales	104.1 (99.9–106.5)*	104.9 (100.0-110.1)*	102.8 (99.6-106.8)[*,7]
Cucurbitales	86.2 (78.8–91.0)*	86.9 (71.3-103.3)*	64.6 (49.8-97.1)[*,7]
N-fixing clade	111.0 (109.2-112.9)*	114.7 (109.8-119.2)*	112.7 (108.6-117.4)[*,7]

Table S3 | List of taxa used in this study with GenBank accession number. "*" represents taxa included in reduced matrix.

Family	Species	Nitrogen-fixing states	*matK*	*rbcL*	*trnL-F*
Ingroup:					
Anisophylleaceae	*Anisophyllea corneri**	lacking nitrogen-fixing symbiosis	AY968444	AF027109	AY968559
Anisophylleaceae	*Combretocarpus rotundatus**	lacking nitrogen-fixing symbiosis	AY968447	AF127698	AY968561
Anisophylleaceae	*Polygonanthus amazonicus*	lacking nitrogen-fixing symbiosis	-	AY973489	-
Anisophylleaceae	*Poga oleosa*	lacking nitrogen-fixing symbiosis	AY973466	AY973488	AY973430
Barbeyaceae	*Barbeya oleoides**	lacking nitrogen-fixing symbiosis	JF317418	U60314	-
Begoniaceae	*Begonia poculifera**	lacking nitrogen-fixing symbiosis	GU397108	-	JN133338
Begoniaceae	*Hillebrandia sandwicensis**	lacking nitrogen-fixing symbiosis	GU397085	-	AY968564
Begoniaceae	*Symbegonia sanguinea**	lacking nitrogen-fixing symbiosis	-	-	AY238596
Betulaceae	*Alnus japonica**	actinorhizal symbiosis	AB038176	FJ844577	AY211427
Betulaceae	*Betula platyphylla**	lacking nitrogen-fixing symbiosis	AY372023	AY263927	AY147068
Betulaceae	*Carpinus betulus**	lacking nitrogen-fixing symbiosis	AJ417513	AY263928	AY211398
Betulaceae	*Corylus avellana**	lacking nitrogen-fixing symbiosis	AY373441	AY263929	AY147072
Betulaceae	*Ostrya virginiana**	lacking nitrogen-fixing symbiosis	AB015460	HQ590198	AY211425
Betulaceae	*Ostryopsis davidiana**	lacking nitrogen-fixing symbiosis	AB015461	AF081515	AY147071
Cannabaceae	*Aphananthe aspera**	lacking nitrogen-fixing symbiosis	AF345320	AF500339	AF501594
Cannabaceae	*Cannabis sativa**	lacking nitrogen-fixing symbiosis	AF345317	AF500344	AF501598
Cannabaceae	*Celtis sinensis**	lacking nitrogen-fixing symbiosis	AF345316	HQ427254	-
Cannabaceae	*Chaetachme aristata**	lacking nitrogen-fixing symbiosis	JF270688	D86310	-
Cannabaceae	*Gironniera subaequalis**	lacking nitrogen-fixing symbiosis	AF345319	AF500340	-
Cannabaceae	*Humulus lupulus**	lacking nitrogen-fixing symbiosis	AY257528	AF206777	AF501599
Cannabaceae	*Lozanella enantiophylla**	lacking nitrogen-fixing symbiosis	-	AF500341	AF501595
Cannabaceae	*Parasponia rigida**	rhizobial symbiosis	-	U59820	AY488675
Cannabaceae	*Pteroceltis tatarinowii**	lacking nitrogen-fixing symbiosis	AF345324	AF500343	AF501597
Cannabaceae	*Trema micrantha**	lacking nitrogen-fixing symbiosis	GQ982115	U03844	AY488708
Casuarinaceae	*Allocasuarina verticillata**	actinorhizal symbiosis	AY191657	X69527	-
Casuarinaceae	*Casuarina equisetifolia**	actinorhizal symbiosis	AB015462	AY263930	AY147090
Casuarinaceae	*Ceuthostoma terminale**	actinorhizal symbiosis	AY033838	AY033860	-
Casuarinaceae	*Gymnostoma webbianum**	actinorhizal symbiosis	AY191680	X69531	-
Coriariaceae	*Coriaria sarmentosa**	actinorhizal symbiosis	AB016464	AF149000	AY968381
Corynocarpaceae	*Corynocarpus laevigata**	lacking nitrogen-fixing symbiosis	NC 014807	NC 014807	NC 014807
Cucurbitaceae	*Actinostemma tenerum*	lacking nitrogen-fixing symbiosis	DQ536631	DQ535779	-
Cucurbitaceae	*Austrobryonia argillicola**	lacking nitrogen-fixing symbiosis	EF487555	EF487548	EF487571
Cucurbitaceae	*Bayabusua clarkei**	lacking nitrogen-fixing symbiosis	DQ536635	DQ535741	-
Cucurbitaceae	*Bolbostemma paniculatum**	lacking nitrogen-fixing symbiosis	DQ469139	DQ501255	-
Cucurbitaceae	*Bryonia dioica**	lacking nitrogen-fixing symbiosis	DQ536641	DQ535786	EU102368
Cucurbitaceae	*Cogniauxia podolaena**	lacking nitrogen-fixing symbiosis	DQ536653	DQ535794	-
Cucurbitaceae	*Cucumis melo**	lacking nitrogen-fixing symbiosis	JF412791	JF412791	HM597025
Cucurbitaceae	*Cucurbita ecuadorensis**	lacking nitrogen-fixing symbiosis	HQ438598	HQ438630	HQ438676
Cucurbitaceae	*Cyclantheropsis parviflora**	lacking nitrogen-fixing symbiosis	EF634361	EF634363	EF634364
Cucurbitaceae	*Ecballium elaterium**	lacking nitrogen-fixing symbiosis	AY973019	AF534746	EU102416
Cucurbitaceae	*Fevillea pergamentacea**	lacking nitrogen-fixing symbiosis	DQ536679	DQ535813	-
Cucurbitaceae	*Halosicyos ragonesei**	lacking nitrogen-fixing symbiosis	DQ536684	DQ535755	DQ535871
Cucurbitaceae	*Herpetospermum pedunculosum**	lacking nitrogen-fixing symbiosis	DQ536687	DQ535818	-
Cucurbitaceae	*Indofevillea khasiana**	lacking nitrogen-fixing symbiosis	-	DQ501256	-
Cucurbitaceae	*Marah macrocarpus**	lacking nitrogen-fixing symbiosis	AY968453	AY968524	AY968571
Cucurbitaceae	*Momordica cochinchinensis**	lacking nitrogen-fixing symbiosis	GQ163378	EF487554	EF487577
Cucurbitaceae	*Neoalsomitra sarcophylla**	lacking nitrogen-fixing symbiosis	AY968454	AY968525	AY968572
Cucurbitaceae	*Nothoalsomitra suberosa**	lacking nitrogen-fixing symbiosis	DQ536709	DQ535762	-
Cucurbitaceae	*Odosicyos bosseri**	lacking nitrogen-fixing symbiosis	DQ536710	DQ535832	-
Cucurbitaceae	*Psiguria bignoniacea**	lacking nitrogen-fixing symbiosis	FJ037900	FJ038017	FJ039219
Cucurbitaceae	*Schizopepon bryoniifolius**	lacking nitrogen-fixing symbiosis	AY968456	AY973025	-

Cucurbitaceae	*Selysia prunifera**	lacking nitrogen-fixing symbiosis	DQ536728	DQ535844	HM164539
Cucurbitaceae	*Sicyos angulatus**	lacking nitrogen-fixing symbiosis	DQ536732	AY862554	-
Cucurbitaceae	*Siraitia grosvenorii**	lacking nitrogen-fixing symbiosis	DQ536736	DQ535850	-
Cucurbitaceae	*Thladiantha cordifolia**	lacking nitrogen-fixing symbiosis	GQ163458	JF944645	-
Cucurbitaceae	*Xerosicyos danguyi**	lacking nitrogen-fixing symbiosis	AY968459	AY973026	AY968573
Cucurbitaceae	*Zanonia indica**	lacking nitrogen-fixing symbiosis	EU436422	EU436396	-
Cucurbitaceae	*Zehneria anomala**	lacking nitrogen-fixing symbiosis	EU541412	EU541411	EU541413
Cucurbitaceae	*Muellerargia timorensis*	lacking nitrogen-fixing symbiosis	DQ536704	DQ535777	HM597062
Cucurbitaceae	*Diplocyclos palmatus*	lacking nitrogen-fixing symbiosis	DQ536671	AY862552	-
Cucurbitaceae	*Indomelothria blumei*	lacking nitrogen-fixing symbiosis	GU799539	GU799532	-
Cucurbitaceae	*Melothria pendula*	lacking nitrogen-fixing symbiosis	DQ536699	DQ535828	-
Cucurbitaceae	*Melancium campestre*	lacking nitrogen-fixing symbiosis	GU799545	GU799537	-
Cucurbitaceae	*Posadaea sphaerocarpa*	lacking nitrogen-fixing symbiosis	GU799544	DQ535836	-
Cucurbitaceae	*Cucumeropsis mannii*	lacking nitrogen-fixing symbiosis	EU436402	GU799534	-
Cucurbitaceae	*Coccinia sessilifolia*	lacking nitrogen-fixing symbiosis	AY968446	AY968520	AY968568
Cucurbitaceae	*Ruthalicia eglandulosa*	lacking nitrogen-fixing symbiosis	EU436418	EU436393	-
Cucurbitaceae	*Scopellaria marginata*	lacking nitrogen-fixing symbiosis	DQ536751	DQ535862	-
Cucurbitaceae	*Papuasicyos papuanus*	lacking nitrogen-fixing symbiosis	EU590121	EU590122	EU590123
Cucurbitaceae	*Peponium vogelii*	lacking nitrogen-fixing symbiosis	HQ608272	DQ535835	-
Cucurbitaceae	*Lagenaria breviflora*	lacking nitrogen-fixing symbiosis	AY935934	AY935747	AY968570
Cucurbitaceae	*Citrullus colocynthis*	lacking nitrogen-fixing symbiosis	DQ536649	DQ535791	-
Cucurbitaceae	*Raphidiocystis phyllocalyx*	lacking nitrogen-fixing symbiosis	-	DQ535839	-
Cucurbitaceae	*Solena heterophylla*	lacking nitrogen-fixing symbiosis	DQ536737	DQ535851	-
Cucurbitaceae	*Lemurosicyos variegatus*	lacking nitrogen-fixing symbiosis	-	DQ501257	-
Cucurbitaceae	*Borneosicyos simplex*	lacking nitrogen-fixing symbiosis	DQ536638	DQ535785	DQ535869
Cucurbitaceae	*Cephalopentandra ecirrhosa*	lacking nitrogen-fixing symbiosis	DQ536645	AF534744	-
Cucurbitaceae	*Acanthosicyos horridus*	lacking nitrogen-fixing symbiosis	DQ536630	DQ535778	-
Cucurbitaceae	*Praecitrullus fistulosus*	lacking nitrogen-fixing symbiosis	DQ536719	DQ535837	-
Cucurbitaceae	*Benincasa hispida*	lacking nitrogen-fixing symbiosis	DQ536636	AY862549	-
Cucurbitaceae	*Trochomeria polymorpha*	lacking nitrogen-fixing symbiosis	EU436420	EU436395	-
Cucurbitaceae	*Dactyliandra welwitschii*	lacking nitrogen-fixing symbiosis	DQ536669	DQ535750	-
Cucurbitaceae	*Ctenolepis cerasiformis*	lacking nitrogen-fixing symbiosis	DQ536656	AY862550	-
Cucurbitaceae	*Zombitsia lucorum*	lacking nitrogen-fixing symbiosis	-	DQ501260	-
Cucurbitaceae	*Anangia macrosepala*	lacking nitrogen-fixing symbiosis	EU436397	-	-
Cucurbitaceae	*Neoachmandra japonica*	lacking nitrogen-fixing symbiosis	DQ536753	DQ535864	-
Cucurbitaceae	*Peponopsis adhaerens*	lacking nitrogen-fixing symbiosis	DQ536716	DQ535766	-
Cucurbitaceae	*Polyclathra cucumerina*	lacking nitrogen-fixing symbiosis	DQ536717	DQ535767	-
Cucurbitaceae	*Sicana odorifera*	lacking nitrogen-fixing symbiosis	DQ536729	DQ535845	-
Cucurbitaceae	*Calycophysum pedunculatum*	lacking nitrogen-fixing symbiosis	-	DQ535743	-
Cucurbitaceae	*Anacaona sphaerica*	lacking nitrogen-fixing symbiosis	DQ536756	EU036995	EU036998
Cucurbitaceae	*Penelopeia suburceolata*	lacking nitrogen-fixing symbiosis	DQ536713	DQ535834	-
Cucurbitaceae	*Tecunumania quetzalteca*	lacking nitrogen-fixing symbiosis	DQ536738	DQ535852	-
Cucurbitaceae	*Schizocarpum palmeri*	lacking nitrogen-fixing symbiosis	DQ536725	DQ535769	-
Cucurbitaceae	*Cionosicyos macranthus*	lacking nitrogen-fixing symbiosis	DQ536648	DQ535790	HM015107
Cucurbitaceae	*Abobra tenuifolia*	lacking nitrogen-fixing symbiosis	DQ536629	AF008961	-
Cucurbitaceae	*Cayaponia africana*	lacking nitrogen-fixing symbiosis	DQ536642	DQ535787	HM015079
Cucurbitaceae	*Eureiandra formosa*	lacking nitrogen-fixing symbiosis	-	DQ535812	-
Cucurbitaceae	*Bambekea racemosa*	lacking nitrogen-fixing symbiosis	-	DQ535783	-
Cucurbitaceae	*Dendrosicyos socotranus*	lacking nitrogen-fixing symbiosis	AY973018	AY973022	-
Cucurbitaceae	*Trochomeriopsis diversifolia*	lacking nitrogen-fixing symbiosis	DQ536746	DQ535859	-
Cucurbitaceae	*Seyrigia humbertii*	lacking nitrogen-fixing symbiosis	AY968457	AY968526	-
Cucurbitaceae	*Corallocarpus bainesii*	lacking nitrogen-fixing symbiosis	DQ536654	DQ535795	-
Cucurbitaceae	*Guraniopsis longipedicellata*	lacking nitrogen-fixing symbiosis	DQ536682	DQ535816	-
Cucurbitaceae	*Apodanthera mandonii*	lacking nitrogen-fixing symbiosis	DQ536634	DQ535782	-
Cucurbitaceae	*Dieterlea maxima*	lacking nitrogen-fixing symbiosis	DQ536670	DQ535807	-
Cucurbitaceae	*Melothrianthus smilacifolius*	lacking nitrogen-fixing symbiosis	DQ536700	DQ535764	-
Cucurbitaceae	*Cucurbitella asperata*	lacking nitrogen-fixing symbiosis	-	DQ535748	-
Cucurbitaceae	*Tumamoca macdougalii*	lacking nitrogen-fixing symbiosis	DQ536747	DQ535860	-

Cucurbitaceae	*Kedrostis africana*	lacking nitrogen-fixing symbiosis	DQ536692	AJ235782	-
Cucurbitaceae	*Ceratosanthes palmata*	lacking nitrogen-fixing symbiosis	DQ536646	DQ535788	-
Cucurbitaceae	*Doyerea emetocathartica*	lacking nitrogen-fixing symbiosis	DQ536672	DQ535808	DQ535870
Cucurbitaceae	*Ibervillea lindheimeri*	lacking nitrogen-fixing symbiosis	DQ536690	DQ535821	-
Cucurbitaceae	*Wilbrandia verticillata*	lacking nitrogen-fixing symbiosis	DQ536749	DQ535861	-
Cucurbitaceae	*Gurania sp.*	lacking nitrogen-fixing symbiosis	FJ037901	FJ038016	FJ039220
Cucurbitaceae	*Helmontia leptantha*	lacking nitrogen-fixing symbiosis	-	DQ535757	DQ521607
Cucurbitaceae	*Biswarea tonglensis*	lacking nitrogen-fixing symbiosis	DQ536637	DQ535742	-
Cucurbitaceae	*Edgaria darjeelingensis*	lacking nitrogen-fixing symbiosis	DQ536677	-	-
Cucurbitaceae	*Trichosanthes villosa*	lacking nitrogen-fixing symbiosis	EU037007	EU037005	EU037008
Cucurbitaceae	*Luffa acutangula*	lacking nitrogen-fixing symbiosis	DQ536695	DQ535826	-
Cucurbitaceae	*Gymnopetalum chinense*	lacking nitrogen-fixing symbiosis	EU155606	EU155601	EU155630
Cucurbitaceae	*Hodgsonia heteroclita*	lacking nitrogen-fixing symbiosis	EU155607	-	EU155631
Cucurbitaceae	*Echinocystis lobata*	lacking nitrogen-fixing symbiosis	AY491653	DQ535809	-
Cucurbitaceae	*Sechiopsis tetraptera*	lacking nitrogen-fixing symbiosis	DQ536726	DQ535842	-
Cucurbitaceae	*Sechium edule*	lacking nitrogen-fixing symbiosis	DQ536727	AY862553	-
Cucurbitaceae	*Parasicyos dieterleae*	lacking nitrogen-fixing symbiosis	DQ536712	DQ535763	-
Cucurbitaceae	*Microsechium helleri*	lacking nitrogen-fixing symbiosis	DQ536701	-	-
Cucurbitaceae	*Sicyosperma gracile*	lacking nitrogen-fixing symbiosis	DQ536734	DQ535772	-
Cucurbitaceae	*Pseudocyclanthera australis*	lacking nitrogen-fixing symbiosis	EF066333	-	-
Cucurbitaceae	*Rytidostylis ciliata*	lacking nitrogen-fixing symbiosis	DQ536724	DQ535841	-
Cucurbitaceae	*Cyclanthera brachystachya*	lacking nitrogen-fixing symbiosis	DQ536667	DQ535749	-
Cucurbitaceae	*Hanburia mexicana*	lacking nitrogen-fixing symbiosis	DQ536685	DQ535756	-
Cucurbitaceae	*Elateriopsis oerstedii*	lacking nitrogen-fixing symbiosis	DQ536678	DQ535752	-
Cucurbitaceae	*Echinopepon wrightii*	lacking nitrogen-fixing symbiosis	DQ536676	DQ535811	DQ536816
Cucurbitaceae	*Frantzia tacaco*	lacking nitrogen-fixing symbiosis	EU436404	EU436380	-
Cucurbitaceae	*Apatzingania arachoidea*	lacking nitrogen-fixing symbiosis	DQ536633	DQ535739	-
Cucurbitaceae	*Brandegea bigelovii*	lacking nitrogen-fixing symbiosis	DQ536639	DQ535866	-
Cucurbitaceae	*Vaseyanthus insularis*	lacking nitrogen-fixing symbiosis	DQ536748	DQ535776	-
Cucurbitaceae	*Telfairia pedata*	lacking nitrogen-fixing symbiosis	-	DQ535853	-
Cucurbitaceae	*Ampelosycios humblotii*	lacking nitrogen-fixing symbiosis	DQ521608	DQ501254	-
Cucurbitaceae	*Tricyclandra leandrii*	lacking nitrogen-fixing symbiosis	-	DQ501259	-
Cucurbitaceae	*Baijiania yunnanensis*	lacking nitrogen-fixing symbiosis	DQ469138	DQ501258	-
Cucurbitaceae	*Hemsleya heterosperma*	lacking nitrogen-fixing symbiosis	DQ536686	DQ535817	-
Cucurbitaceae	*Gomphogyne cirromitrata*	lacking nitrogen-fixing symbiosis	EU436405	EU436381	-
Cucurbitaceae	*Gynostemma pentaphyllum*	lacking nitrogen-fixing symbiosis	AY968451	AY968523	-
Cucurbitaceae	*Alsomitra macrocarpa*	lacking nitrogen-fixing symbiosis	DQ536632	DQ535780	-
Cucurbitaceae	*Chalema synanthera*	lacking nitrogen-fixing symbiosis	DQ536647	DQ535789	-
Cucurbitaceae	*Sicydium tamnifolium*	lacking nitrogen-fixing symbiosis	DQ536731	DQ535846	-
Cucurbitaceae	*Pseudosicydium acariianthum*	lacking nitrogen-fixing symbiosis	EU436417	EU436392	-
Cucurbitaceae	*Pteropepon parodii*	lacking nitrogen-fixing symbiosis	DQ536722	DQ535838	-
Cucurbitaceae	*Gerrardanthus grandiflorus*	lacking nitrogen-fixing symbiosis	GQ163343	DQ535805	-
Cucurbitaceae	*Siolmatra brasiliensis*	lacking nitrogen-fixing symbiosis	DQ536735	DQ535849	-
Cucurbitaceae	*Zygosicyos tripartitus*	lacking nitrogen-fixing symbiosis	DQ536755	DQ535732	-
Cucurbitaceae	*Cucumella aspera*	lacking nitrogen-fixing symbiosis	DQ785842	-	-
Cucurbitaceae	*Oreosyce africana*	lacking nitrogen-fixing symbiosis	DQ785853	-	-
Cucurbitaceae	*Myrmecosicyos messorius*	lacking nitrogen-fixing symbiosis	-	-	DQ535872
Cucurbitaceae	*Mukia javanica*	lacking nitrogen-fixing symbiosis	EF174477		EF174485
Datiscaceae	*Datisca glomerata**	actinorhizal symbiosis	AY968449	L21940	AY968567
Dirachmaceae	*Dirachma socotrana**	lacking nitrogen-fixing symbiosis	JF317423	AJ225789	-
Elaeagnaceae	*Elaeagnus umbellata**	actinorhizal symbiosis	AY257529	HM849968	HM769678
Elaeagnaceae	*Hippophae rhamnoides subsp. sinensis**	actinorhizal symbiosis	JF317428	JF317488	GU561430
Elaeagnaceae	*Shepherdia canadensis**	actinorhizal symbiosis	-	U17039	GQ245525
Fabaceae	*Amherstia nobilis**	lacking nitrogen-fixing symbiosis	EU361849	AM234234	AF365210
Fabaceae	*Amorpha fruticosa**	rhizobial symbiosis	AF270861	U74212	AF208899
Fabaceae	*Apuleia leiocarpa**	lacking nitrogen-fixing symbiosis	EU361858	U74249	EU361737
Fabaceae	*Ateleia herbert-smithii**	rhizobial symbiosis	AY386953	U74201	AF309840

Fabaceae	*Baphia massaiensis**	rhizobial symbiosis	AF142683	U74196	AF309860
Fabaceae	*Caesalpinia calycina**	lacking nitrogen-fixing symbiosis	EU361899	AM234236	AY899691
Fabaceae	*Cassia grandis**	lacking nitrogen-fixing symbiosis	EU361909	AM234244	AF365092
Fabaceae	*Cercis canadensis**	lacking nitrogen-fixing symbiosis	EU361912	U74188	AF365054
Fabaceae	*Cicer arietinum**	rhizobial symbiosis	EU835853	EU835853	DQ315487
Fabaceae	*Cladrastis delavayi**	rhizobial symbiosis	AY386861	-	AF311370
Fabaceae	*Cyclolobium nutans**	rhizobial symbiosis	AF142686	-	AF309857
Fabaceae	*Dalbergia sissoo**	rhizobial symbiosis	AF203582	GU135159	EF451118
Fabaceae	*Detarium macrocarpum**	lacking nitrogen-fixing symbiosis	EU361929	AM234239	AF365182
Fabaceae	*Duparquetia orchidacea**	lacking nitrogen-fixing symbiosis	EU361937	-	EU361800
Fabaceae	*Genista monspessulana**	rhizobial symbiosis	AY386862	HM850024	JF338274
Fabaceae	*Griffonia physocarpa**	lacking nitrogen-fixing symbiosis	EU361961	AM234265	EU361813
Fabaceae	*Hypocalyptus coluteoides**	rhizobial symbiosis	AY386886	-	-
Fabaceae	*Indigofera fulgens**	rhizobial symbiosis	JF270831	JF265484	-
Fabaceae	*Isotropis cuneifolia**	rhizobial symbiosis	AF298483	AF308712	-
Fabaceae	*Lecointea peruviana**	lacking nitrogen-fixing symbiosis	EU361990	AM234260	AF365039
Fabaceae	*Lotus japonicus**	rhizobial symbiosis	NC 002694	NC 002694	NC 002694
Fabaceae	*Millettia pinnata**	rhizobial symbiosis	NC 016708	NC 016708	NC 016708
Fabaceae	*Mimosa pigra**	rhizobial symbiosis	GU135076	GU135239	DQ344560
Fabaceae	*Myrospermum sousanum**	undetermined	AY386959	U74207	AF309851
Fabaceae	*Ormosia macrocalyx**	rhizobial symbiosis	GQ982056	GQ981822	-
Fabaceae	*Pentaclethra macrophylla**	rhizobial symbiosis	AF521853	AM234250	AF278485
Fabaceae	*Poeppigia procera**	lacking nitrogen-fixing symbiosis	EU362026	AM234246	AY899682
Fabaceae	*Ptychosema anomalum**	undetermined	-	-	AF518165
Fabaceae	*Pultenaea reticulata**	rhizobial symbiosis	AF298517	-	AF113790
Fabaceae	*Robinia pseudoacacia**	rhizobial symbiosis	AF142728	U74220	AF529391
Fabaceae	*Styphnolobium japonicum**	rhizobial symbiosis	AY386954	GQ436343	EF466270
Fabaceae	*Swartzia cardiosperma**	rhizobial symbiosis	EU362053	AM234259	AF365040
Fabaceae	*Trifolium burchellianum**	rhizobial symbiosis	AF522118	AM235018	DQ311759
Fabaceae	*Xeroderris stuhlmannii**	rhizobial symbiosis	AF142708	AF308727	-
Fabaceae	*Strophostyles umbellata*	rhizobial symbiosis	DQ443470	-	-
Fabaceae	*Dolichopsis paraguariensis*	rhizobial symbiosis	AY509942	-	-
Fabaceae	*Mysanthus uleanus*	undetermined	AY509941	-	-
Fabaceae	*Macroptilium atropurpureum*	rhizobial symbiosis	EU717409	EU717268	-
Fabaceae	*Helicotropis linearis*	rhizobial symbiosis	JN008258	-	-
Fabaceae	*Leptospron adenanthum*	rhizobial symbiosis	JN008271	-	-
Fabaceae	*Sigmoidotropis ampla*	rhizobial symbiosis	JN008240	-	-
Fabaceae	*Ancistrotropis peduncularis*	rhizobial symbiosis	JN008272	-	-
Fabaceae	*Condylostylis venusta*	rhizobial symbiosis	JN008261	-	-
Fabaceae	*Cochliasanthus caracalla*	rhizobial symbiosis	JN008274	-	-
Fabaceae	*Phaseolus vulgaris*	rhizobial symbiosis	DQ886273	DQ886273	AY077945
Fabaceae	*Oxyrhynchus volubilis*	rhizobial symbiosis	AY509935	AF308717	-
Fabaceae	*Ramirezella nitida*	rhizobial symbiosis	JN008263	-	-
Fabaceae	*Vigna unguiculata*	rhizobial symbiosis	AY589510	EU717266	GQ411554
Fabaceae	*Physostigma venenosum*	lacking nitrogen-fixing symbiosis	AY582998	-	-
Fabaceae	*Vatovaea pseudolablab*	undetermined	AH013772	-	-
Fabaceae	*Spathionema kilimandscharicum*	undetermined	AY582990	-	-
Fabaceae	*Lablab purpureus*	rhizobial symbiosis	EU717408	EU717267	-
Fabaceae	*Dipogon lignosus*	rhizobial symbiosis	AY582988	AB045800	-
Fabaceae	*Wajira grahamiana*	rhizobial symbiosis	AY583005	-	-
Fabaceae	*Sphenostylis angustifolia*	rhizobial symbiosis	AY582978	-	-
Fabaceae	*Macrotyloma uniflorum*	rhizobial symbiosis	EU717410	EU717269	-
Fabaceae	*Decorsea schlechteri*	rhizobial symbiosis	AY582975	-	-
Fabaceae	*Alistilus jumellei*	rhizobial symbiosis	JN008191	-	-
Fabaceae	*Nesphostylis holosericea*	undetermined	AY582979	-	-
Fabaceae	*Dolichos oliveri*	rhizobial symbiosis	JN008183	-	-
Fabaceae	*Strongylodon macrobotrys*	rhizobial symbiosis	-	AF308729	-
Fabaceae	*Cologania lemmonii*	rhizobial symbiosis	EU717405	EU717264	-

Fabaceae	*Dumasia villosa*	rhizobial symbiosis	EU717406	EU717265	-
Fabaceae	*Pueraria phaseoloides*	rhizobial symbiosis	EU717404	EU717263	-
Fabaceae	*Pseudovigna argentea*	rhizobial symbiosis	EU717403	EU717262	-
Fabaceae	*Neorautanenia mitis*	rhizobial symbiosis	JN008178	AF308715	-
Fabaceae	*Calopogonium caeruleum*	rhizobial symbiosis	-	AF308723	-
Fabaceae	*Pachyrhizus erosus*	rhizobial symbiosis	EU717401	EU717260	-
Fabaceae	*Neonotonia wightii*	rhizobial symbiosis	EU717402	EU717261	-
Fabaceae	*Amphicarpaea bracteata*	rhizobial symbiosis	EU717399	EU717257	AF417049
Fabaceae	*Phylacium majus*	undetermined	-	AB045815	-
Fabaceae	*Teramnus uncinatus*	rhizobial symbiosis	EU717400	EU717258	-
Fabaceae	*Glycine max*	rhizobial symbiosis	DQ317523	DQ317523	DQ131547
Fabaceae	*Bituminaria bituminosa*	rhizobial symbiosis	EU717398	U74221	-
Fabaceae	*Otholobium bolusii*	rhizobial symbiosis	JN008180	-	-
Fabaceae	*Psoralidium tenuiflorum*	rhizobial symbiosis	EF549987	-	-
Fabaceae	*Pediomelum argophyllum*	rhizobial symbiosis	EF549988	-	HM590336
Fabaceae	*Rupertia physodes*	undetermined	EF549955	-	-
Fabaceae	*Cullen australasicum*	rhizobial symbiosis	EF550002	EU717254	-
Fabaceae	*Psoralea cinerea*	rhizobial symbiosis	AF142699	-	-
Fabaceae	*Orbexilum melanocarpum*	rhizobial symbiosis	EF549932	-	-
Fabaceae	*Hoita macrostachya*	rhizobial symbiosis	EF549954	-	-
Fabaceae	*Otoptera burchellii*	rhizobial symbiosis	JN008176	-	-
Fabaceae	*Psophocarpus tetragonolobus*	rhizobial symbiosis	EU717412	EU717271	-
Fabaceae	*Erythrina sousae*	rhizobial symbiosis	EU717411	EU717270	-
Fabaceae	*Cajanus cajan*	rhizobial symbiosis	EU717414	EU717273	-
Fabaceae	*Bolusafra bituminosa*	rhizobial symbiosis	EU717413	EU717272	-
Fabaceae	*Rhynchosia clivorum*	rhizobial symbiosis	-	AF308720	-
Fabaceae	*Eriosema squarrosum*	rhizobial symbiosis	-	AM235007	-
Fabaceae	*Flemingia glutinosa*	rhizobial symbiosis	-	GQ436333	-
Fabaceae	*Adenodolichos rupestris*	rhizobial symbiosis	-	AF308700	-
Fabaceae	*Butea monosperma*	rhizobial symbiosis	JN008175	-	-
Fabaceae	*Spatholobus parviflorus*	rhizobial symbiosis	EU106113	AB045825	-
Fabaceae	*Kennedia nigricans*	rhizobial symbiosis	EU717424	EU717283	-
Fabaceae	*Hardenbergia violacea*	rhizobial symbiosis	EU717425	U74241	-
Fabaceae	*Shuteria vestita*	rhizobial symbiosis	EU717423	AF308725	-
Fabaceae	*Mucuna sp.*	rhizobial symbiosis	EU717422	EU717281	-
Fabaceae	*Craspedolobium schochii*	undetermined	JF953574	JF941298	-
Fabaceae	*Kummerowia stipulacea*	rhizobial symbiosis	EU717417	U74229	-
Fabaceae	*Lespedeza cuneata*	rhizobial symbiosis	EU717416	U74215	-
Fabaceae	*Campylotropis macrocarpa*	rhizobial symbiosis	EU717418	EU717277	-
Fabaceae	*Tadehagi triquetrum*	rhizobial symbiosis	JN407128	GQ436342	-
Fabaceae	*Phyllodium pulchellum*	rhizobial symbiosis	HM049524	-	-
Fabaceae	*Hylodesmum podocarpum*	rhizobial symbiosis	GU572325	-	-
Fabaceae	*Desmodium pauciflorum*	rhizobial symbiosis	EU717421	EU717280	-
Fabaceae	*Pseudarthria hookeri*	rhizobial symbiosis	JF270902	JF265559	-
Fabaceae	*Alysicarpus ovalifolius*	rhizobial symbiosis	-	JN628036	-
Fabaceae	*Uraria crinita*	rhizobial symbiosis	JN407138.2	JN407297	-
Fabaceae	*Apios americana*	rhizobial symbiosis	EU717426	EU717285	-
Fabaceae	*Dewevrea bilabiata*	rhizobial symbiosis	-	AB045799	-
Fabaceae	*Platycyamus regnellii*	rhizobial symbiosis	AF142709	AB045817	AF311378
Fabaceae	*Disynstemon paullinioides*	undetermined	GU951670	-	-
Fabaceae	*Abrus precatorius*	rhizobial symbiosis	AF142705	U74224	-
Fabaceae	*Galactia striata*	rhizobial symbiosis	EU717428	EU717287	FJ613502
Fabaceae	*Collaea argentina*	rhizobial symbiosis	-	-	FJ613490
Fabaceae	*Rhodopis planisiliqua*	undetermined	-	AF308728	-
Fabaceae	*Camptosema coccineum*	rhizobial symbiosis	-	-	FJ613485
Fabaceae	*Dioclea reflexa*	rhizobial symbiosis	HQ707540	-	-
Fabaceae	*Canavalia rosea*	rhizobial symbiosis	HQ707517	AB045793	-
Fabaceae	*Philenoptera violacea*	rhizobial symbiosis	JF270890	JF265547	-

Fabaceae	*Capassa violacea*	rhizobial symbiosis	AF142719	-	-
Fabaceae	*Leptoderris fasciculata*	rhizobial symbiosis	-	AB045807	-
Fabaceae	*Ophrestia radicosa*	rhizobial symbiosis	EU717430	AF308726	-
Fabaceae	*Hesperothamnus pentaphyllus*	undetermined	-	AB045805	-
Fabaceae	*Mundulea sericea*	rhizobial symbiosis	AF142713	AB045814	AY009136
Fabaceae	*Chadsia versicolor*	rhizobial symbiosis	-	AB045794	-
Fabaceae	*Tephrosia rhodesica*	rhizobial symbiosis	EU717429	EU717288	-
Fabaceae	*Apurimacia dolichocarpa*	undetermined	FJ968527	-	-
Fabaceae	*Piscidia piscipula*	rhizobial symbiosis	AF142710	AB045816	AF311379
Fabaceae	*Neodunnia richardiana*	undetermined	AF142726	-	-
Fabaceae	*Pongamiopsis amygdalina*	undetermined	AF142711	AB045819	-
Fabaceae	*Muellera frutescens*	undetermined	-	AB045813	-
Fabaceae	*Willardia mexicana*	undetermined	-	AF308721	-
Fabaceae	*Lonchocarpus heptaphyllus*	rhizobial symbiosis	HM446705	GQ981789	-
Fabaceae	*Deguelia hatschbachii*	rhizobial symbiosis	-	AB045798	-
Fabaceae	*Derris laxiflora*	rhizobial symbiosis	AF142715	U74234	-
Fabaceae	*Aganope sp.*	rhizobial symbiosis	-	AF308702	-
Fabaceae	*Brachypterum robustum*	rhizobial symbiosis	AF142716	-	-
Fabaceae	*Pongamia pinnata*	rhizobial symbiosis	-	AY289676	-
Fabaceae	*Fordia cauliflora*	undetermined	HM049511	AB045802	-
Fabaceae	*Austrosteenisia blackii*	undetermined	AF142707	U74242	AF311381
Fabaceae	*Dalbergiella nyasae*	rhizobial symbiosis	AF142706	AF308724	-
Fabaceae	*Centrosema virginianum*	rhizobial symbiosis	-	AF308706	-
Fabaceae	*Clitoria ternatea*	rhizobial symbiosis	EU717427	U74237	-
Fabaceae	*Craibia brevicaudata*	rhizobial symbiosis	-	AB045795	-
Fabaceae	*Cyamopsis senegalensis*	rhizobial symbiosis	AF142698	-	-
Fabaceae	*Microcharis karinensis*	rhizobial symbiosis	AY650279	-	-
Fabaceae	*Phylloxylon spinosa*	undetermined	AY650280	-	AF274358
Fabaceae	*Vaughania perreri*	undetermined	-	-	AF274372
Fabaceae	*Rhynchotropis poggei*	undetermined	-	-	AF274361
Fabaceae	*Indigastrum argyraeum*	rhizobial symbiosis	-	-	AF274364
Fabaceae	*Genistidium dumosum*	undetermined	AF543858	-	AF529394
Fabaceae	*Peteria thompsoniae*	undetermined	AF547190	-	AF529395
Fabaceae	*Coursetia hypoleuca*	rhizobial symbiosis	AF547193	-	AF529410
Fabaceae	*Sphinctospermum constrictum*	undetermined	AF547191	-	AF529392
Fabaceae	*Olneya tesota*	rhizobial symbiosis	AF543859	-	AF529393
Fabaceae	*Hebestigma cubense*	rhizobial symbiosis	AF543850	-	AF400134
Fabaceae	*Poitea glyciphylla*	rhizobial symbiosis	AY650278	-	AF400144
Fabaceae	*Hybosema robustum*	undetermined	AF547194	-	AF400137
Fabaceae	*Gliricidia sepium*	rhizobial symbiosis	AF547197	JF738386	AF400138
Fabaceae	*Lennea modesta*	undetermined	AF543851	-	AF400135
Fabaceae	*Sesbania sesban*	rhizobial symbiosis	HQ730422	Z95541	-
Fabaceae	*Hippocrepis comosa*	rhizobial symbiosis	JN894094	JN890863	-
Fabaceae	*Coronilla varia*	rhizobial symbiosis	-	U74222	-
Fabaceae	*Securigera varia*	rhizobial symbiosis	AF543846	-	-
Fabaceae	*Anthyllis vulneraria*	rhizobial symbiosis	JN894822	JN892206	HQ323827
Fabaceae	*Ornithopus compressus*	rhizobial symbiosis	AF142727	HM850215	-
Fabaceae	*Dorycnium pentaphyllum*	rhizobial symbiosis	-	FR865124	-
Fabaceae	*Endosamara racemosa*	undetermined	-	AY308805	-
Fabaceae	*Wisteria sinensis*	rhizobial symbiosis	AF142732	Z95544	-
Fabaceae	*Callerya reticulata*	rhizobial symbiosis	AF142733	-	AF311380
Fabaceae	*Afgekia sericea*	undetermined	-	AB045785	-
Fabaceae	*Glycyrrhiza lepidota*	rhizobial symbiosis	AF142730	AB126685	AF124238
Fabaceae	*Melilotus alba*	rhizobial symbiosis	AF142738	DQ006095	DQ311713
Fabaceae	*Trigonella caerulea*	rhizobial symbiosis	AF522143.2	-	GQ488615
Fabaceae	*Medicago lupulina*	rhizobial symbiosis	HM159569	AY395551	JQ041869
Fabaceae	*Ononis repens*	rhizobial symbiosis	JN894068	JN890826	-
Fabaceae	*Lathyrus sativus*	rhizobial symbiosis	HM029371	HM029371	DQ311696

Fabaceae	*Pisum sativum*	rhizobial symbiosis	HM029370	HM029370	DQ311717
Fabaceae	*Lens culinaris*	rhizobial symbiosis	AF522089	-	-
Fabaceae	*Vicia villosa*	rhizobial symbiosis	AF522161	HM850464	DQ311955
Fabaceae	*Galega orientalis*	rhizobial symbiosis	AF522083	-	DQ311694
Fabaceae	*Parochetus communis*	rhizobial symbiosis	AF522115	-	DQ311716
Fabaceae	*Caragana arborescens*	rhizobial symbiosis	AF142737	FJ537211	DQ311691
Fabaceae	*Halimodendron halodendron*	rhizobial symbiosis	-	FJ537237	-
Fabaceae	*Calophaca soongorica*	rhizobial symbiosis	-	FJ537236	-
Fabaceae	*Alhagi maurorum*	rhizobial symbiosis	AY386880	-	-
Fabaceae	*Onobrychis montana*	rhizobial symbiosis	AY386879	-	HM542660
Fabaceae	*Hedysarum vicioides*	rhizobial symbiosis	HM142267	U74246	-
Fabaceae	*Gueldenstaedtia verna subsp. Multiflora*	rhizobial symbiosis	HM049545	GQ436359	-
Fabaceae	*Biserrula pelecinus*	rhizobial symbiosis	-	-	AF126995
Fabaceae	*Astragalus adsurgens*	rhizobial symbiosis	HM142258	EF685984	AF126980
Fabaceae	*Oxytropis anertii*	rhizobial symbiosis	HM142266	HM142226	-
Fabaceae	*Carmichaelia williamsii*	rhizobial symbiosis	AY386873	-	AF127000
Fabaceae	*Clianthus puniceus*	rhizobial symbiosis	AY386914	-	AF126998
Fabaceae	*Swainsona pterostylis*	rhizobial symbiosis	AF142735	-	AF126999
Fabaceae	*Sphaerophysa salsula*	rhizobial symbiosis	-	-	AF126996
Fabaceae	*Colutea arborescens*	rhizobial symbiosis	AY386874	-	AF126993
Fabaceae	*Lessertia herbacea*	rhizobial symbiosis	AY920453	-	AF126997
Fabaceae	*Sutherlandia frutescens*	rhizobial symbiosis	AY386913	-	AF126994
Fabaceae	*Chorizema cordatum*	rhizobial symbiosis	-	U74218	-
Fabaceae	*Goodia lotifolia*	rhizobial symbiosis	-	U74258	-
Fabaceae	*Daviesia latifolia*	rhizobial symbiosis	AY386887	-	-
Fabaceae	*Bossiaea cordigera*	rhizobial symbiosis	AY386888	-	-
Fabaceae	*Gompholobium minus*	rhizobial symbiosis	AY386891	-	-
Fabaceae	*Dillwynia parvifolia*	rhizobial symbiosis	-	-	AF113777
Fabaceae	*Eutaxia microphylla*	rhizobial symbiosis	-	-	AF113780
Fabaceae	*Urodon capitatus*	undetermined	-	-	AF113792
Fabaceae	*Euchilopsis linearis*	rhizobial symbiosis	-	-	AF113779
Fabaceae	*Phyllota phylicoides*	rhizobial symbiosis	AF298509	-	AF113785
Fabaceae	*Aotus ericoides*	rhizobial symbiosis	AY386884	-	-
Fabaceae	*Latrobea hirtella*	rhizobial symbiosis	AF298484	-	AF113781
Fabaceae	*Stonesiella selaginoides*	undetermined	-	-	AF113791
Fabaceae	*Almaleea cambagei*	rhizobial symbiosis	-	-	AF113775
Fabaceae	*Oxylobium ellipticum*	rhizobial symbiosis	AF298505	-	AF113784
Fabaceae	*Gastrolobium punctatum*	rhizobial symbiosis	AY386885	-	-
Fabaceae	*Brachysema melanopetalum*	rhizobial symbiosis	AF298427	-	-
Fabaceae	*Jansonia formosa*	undetermined	AF298482	-	-
Fabaceae	*Nemcia alternifolia*	rhizobial symbiosis	AF298487	-	-
Fabaceae	*Podolobium aestivum*	rhizobial symbiosis	AF298511	-	-
Fabaceae	*Callistachys lanceolata*	rhizobial symbiosis	AF298433	-	-
Fabaceae	*Mirbelia depressa*	rhizobial symbiosis	AF298485	-	-
Fabaceae	*Jacksonia horrida*	rhizobial symbiosis	AF298481	-	-
Fabaceae	*Dalhousiea africana*	lacking nitrogen-fixing symbiosis	-	-	AF310998
Fabaceae	*Airyantha schweinfurthii*	undetermined	-	-	AF310997
Fabaceae	*Baphiopsis parviflora*	lacking nitrogen-fixing symbiosis	-	-	AF309861
Fabaceae	*Leucomphalos callicarpus*	lacking nitrogen-fixing symbiosis	-	-	AF309862
Fabaceae	*Pericopsis mooniana*	rhizobial symbiosis	-	U74210	-
Fabaceae	*Plagiocarpus axillaris*	undetermined	GQ246160	-	-
Fabaceae	*Lamprolobium fruticosum*	rhizobial symbiosis	GQ246159	-	-
Fabaceae	*Templetonia hookeri*	rhizobial symbiosis	GQ246157	-	-
Fabaceae	*Hovea purpurea*	rhizobial symbiosis	AY386889	-	-
Fabaceae	*Brongniartia alamosana*	rhizobial symbiosis	AF142688	-	-
Fabaceae	*Harpalyce arborescens*	rhizobial symbiosis	AF142689	-	-
Fabaceae	*Tabaroa caatingicola*	undetermined	GQ246162	-	-

Fabaceae	*Poecilanthe parvifolia*	rhizobial symbiosis	AF142687	-	-
Fabaceae	*Bowdichia virgilioides*	rhizobial symbiosis	AY386937	-	AF309486
Fabaceae	*Acosmium panamense*	rhizobial symbiosis	AF142684	-	AF208891
Fabaceae	*Diplotropis brasiliensis*	rhizobial symbiosis	AY386939	-	-
Fabaceae	*Clathrotropis brachypetala*	rhizobial symbiosis	-	-	AF309827
Fabaceae	*Sophora flavescens*	rhizobial symbiosis	HM049520	AB127037	AB127029
Fabaceae	*Echinosophora koreensis*	undetermined	-	AB127036	AB127028
Fabaceae	*Euchresta formosana*	rhizobial symbiosis	-	AB127039	AB127031
Fabaceae	*Ammodendron argenteum*	undetermined	AY386957	-	-
Fabaceae	*Maackia amurensis*	rhizobial symbiosis	AY386944	-	-
Fabaceae	*Piptanthus nepalensis*	rhizobial symbiosis	AY386924	-	-
Fabaceae	*Thermopsis rhombifolia*	rhizobial symbiosis	AY386866	-	HM590355
Fabaceae	*Baptisia australis*	rhizobial symbiosis	AY386900	-	AF309831
Fabaceae	*Salweenia wardii*	undetermined	-	U74251	-
Fabaceae	*Platycelyphium voense*	undetermined	-	-	AF309864
Fabaceae	*Dicraeopetalum stipulare*	undetermined	GQ246142	-	AF310995
Fabaceae	*Bolusanthus speciosus*	rhizobial symbiosis	AF142685	JF265305	AF310994
Fabaceae	*Liparia splendens subsp. comantha*	rhizobial symbiosis	-	AM259363	-
Fabaceae	*Podalyria calyptrata*	rhizobial symbiosis	-	U74217	-
Fabaceae	*Amphithalea phylicoides*	rhizobial symbiosis	-	AM180190	-
Fabaceae	*Stirtonanthus taylorianus*	undetermined	-	AM259369	-
Fabaceae	*Xiphotheca phylicoides*	rhizobial symbiosis	-	AM260746	-
Fabaceae	*Cyclopia galioides*	rhizobial symbiosis	-	AM261715	-
Fabaceae	*Cadia purpurea*	rhizobial symbiosis	-	U74192	AF309863
Fabaceae	*Virgilia oroboides subsp. oroboides*	rhizobial symbiosis	-	AM260739	-
Fabaceae	*Calpurnia aurea*	rhizobial symbiosis	AY386951	U74239	AF310993
Fabaceae	*Rafnia perfoliata*	rhizobial symbiosis	-	AM235014	-
Fabaceae	*Lotononis galpinii*	rhizobial symbiosis	-	Z95538	-
Fabaceae	*Crotalaria trichotoma*	rhizobial symbiosis	HM049507	GQ436334	-
Fabaceae	*Lebeckia sericea*	rhizobial symbiosis	GQ246144	-	-
Fabaceae	*Aspalathus linearis*	rhizobial symbiosis	-	-	FJ620662
Fabaceae	*Argyrolobium uniflorum*	rhizobial symbiosis	-	Z95548	EU341594
Fabaceae	*Melolobium microphyllum*	rhizobial symbiosis	-	Z95539	-
Fabaceae	*Dichilus lebeckioides*	rhizobial symbiosis	GQ246143	U74223	-
Fabaceae	*Anarthrophyllum desideratum*	undetermined	AY386923	-	-
Fabaceae	*Polhillia pallens*	undetermined	-	AM235012	-
Fabaceae	*Lupinus luteus*	rhizobial symbiosis	HM851129	HM850145	DQ417012
Fabaceae	*Laburnum anagyroides*	rhizobial symbiosis	FR869998	FR869999	DQ417004
Fabaceae	*Calicotome villosa*	rhizobial symbiosis	-	-	JF338229
Fabaceae	*Chamaecytisus albidus*	rhizobial symbiosis	-	-	DQ417003
Fabaceae	*Cytisus scoparius*	rhizobial symbiosis	AY386902	HM849943	JF338224
Fabaceae	*Spartium junceum*	rhizobial symbiosis	AY386901	HM850377	DQ417002
Fabaceae	*Echinospartum boissieri*	undetermined	-	-	AF385415
Fabaceae	*Adenocarpus telonensis*	rhizobial symbiosis	-	Z95545	-
Fabaceae	*Retama monosperma*	rhizobial symbiosis	-	-	JF338259
Fabaceae	*Stauracanthus genistoides subsp. Genistoides*	rhizobial symbiosis	-	-	AF385417
Fabaceae	*Ulex minor*	rhizobial symbiosis	HM851133	HM850432	AF385419
Fabaceae	*Errazurizia benthamii*	undetermined	AY391803	-	-
Fabaceae	*Eysenhardtia texana*	rhizobial symbiosis	AY391807	-	-
Fabaceae	*Apoplanesia paniculata*	undetermined	AF270860	-	AF208898
Fabaceae	*Parryella filifolia*	rhizobial symbiosis	AY391812	-	-
Fabaceae	*Psorothamnus polydenius var. jonesii*	rhizobial symbiosis	AY391819	-	-
Fabaceae	*Dalea wrightii*	rhizobial symbiosis	AY391802	-	-
Fabaceae	*Marina calycosa*	rhizobial symbiosis	AY391808	-	-

Fabaceae	*Zornia sp.*	rhizobial symbiosis	AF203584	-	AF208903
Fabaceae	*Amicia glandulosa*	rhizobial symbiosis	AF203583	-	AF208902
Fabaceae	*Poiretia angustifolia*	rhizobial symbiosis	AF270864	-	AF208904
Fabaceae	*Nissolia schottii*	lacking nitrogen-fixing symbiosis	AF270867	-	AF208907
Fabaceae	*Chaetocalyx blanchetiana*	lacking nitrogen-fixing symbiosis	AF272070	-	AF208905
Fabaceae	*Adesmia lanata*	rhizobial symbiosis	AF270863	-	AF208901
Fabaceae	*Cyclocarpa stellaris*	rhizobial symbiosis	AF272067	-	-
Fabaceae	*Soemmeringia semperflorens*	undetermined	AH009909	-	AF208937
Fabaceae	*Geissaspis descampsii*	rhizobial symbiosis	AF272064	-	AF208931
Fabaceae	*Humularia corbisieri*	rhizobial symbiosis	AF272069	-	AF208936
Fabaceae	*Bryaspis lupulina*	rhizobial symbiosis	AF272068	-	AF208932
Fabaceae	*Kotschya ochreata*	rhizobial symbiosis	AF272065	-	AF208935
Fabaceae	*Smithia ciliata*	rhizobial symbiosis	AF272066	-	AF208933
Fabaceae	*Aeschynomene indica*	rhizobial symbiosis	AH009907	AF308701	AF208927
Fabaceae	*Pictetia aculeata*	undetermined	AF203577	-	AF260906
Fabaceae	*Diphysa ormocarpoides*	rhizobial symbiosis	AF203601	-	AF208912
Fabaceae	*Ormocarpum trichocarpum*	rhizobial symbiosis	JF270878	JF265535	-
Fabaceae	*Peltiera nitida*	undetermined	GU951672	-	-
Fabaceae	*Ormocarpopsis itremoensis*	undetermined	AF203567	-	AF208918
Fabaceae	*Machaerium sp.*	rhizobial symbiosis	AF142692	-	AF208925
Fabaceae	*Weberbauerella brongniartioides*	undetermined	AH009903	-	AF208909
Fabaceae	*Discolobium psoraleifolium*	rhizobial symbiosis	AF270874	-	AF208964
Fabaceae	*Riedeliella graciliflora*	rhizobial symbiosis	AH009910	-	AF208949
Fabaceae	*Stylosanthes hamata*	rhizobial symbiosis	AF203594	-	AF208944
Fabaceae	*Arachis hypogaea*	rhizobial symbiosis	EU307349	U74247	DQ131546
Fabaceae	*Chapmannia sericea*	rhizobial symbiosis	AF203591	-	AF208943
Fabaceae	*Fissicalyx fendleri*	undetermined	AF272063	-	AF208938
Fabaceae	*Fiebrigiella gracilis*	undetermined	AF203590	-	AF208939
Fabaceae	*Geoffroea decorticans*	rhizobial symbiosis	AF270880	-	AF208962
Fabaceae	*Cascaronia astragalina*	undetermined	AF272072	-	AF208958
Fabaceae	*Platymiscium pinnatum*	rhizobial symbiosis	EU735966	FJ038050	EU736022
Fabaceae	*Cranocarpus martii*	rhizobial symbiosis	AF270875	AB045796	AF208951
Fabaceae	*Brya ebenus*	rhizobial symbiosis	AF270876	AB045788	AF208950
Fabaceae	*Grazielodendron riodocense*	lacking nitrogen-fixing symbiosis	AF270862	-	AF208952
Fabaceae	*Platypodium elegans*	rhizobial symbiosis	AF270877	GQ981836	AF208961
Fabaceae	*Paramachaerium schomburgkii*	rhizobial symbiosis	AF272062	-	AF208959
Fabaceae	*Etaballia guianensis*	rhizobial symbiosis	AH009902	-	AF208960
Fabaceae	*Pterocarpus indicus*	rhizobial symbiosis	AF142691	JF738773	AF208953
Fabaceae	*Maraniona lavinii*	undetermined	AY247263	-	-
Fabaceae	*Inocarpus fagifer*	rhizobial symbiosis	AF270878	-	AF208965
Fabaceae	*Tipuana tipu*	rhizobial symbiosis	AF270882	-	AF208956
Fabaceae	*Ramorinoa girolae*	undetermined	AF270881	-	AF208957
Fabaceae	*Centrolobium sp.*	rhizobial symbiosis	AF270883	-	AF208966
Fabaceae	*Zollernia splendens*	rhizobial symbiosis	-	-	AF311372
Fabaceae	*Uribea tamarindoides*	undetermined	AY553719	-	AF311000
Fabaceae	*Holocalyx balansae*	lacking nitrogen-fixing symbiosis	AY553714	U74244	EF466269
Fabaceae	*Hymenolobium mesoamericanum*	rhizobial symbiosis	AH009905	-	AF309852
Fabaceae	*Vataireopsis surinamensis*	lacking nitrogen-fixing symbiosis	AF142680	-	AF208894
Fabaceae	*Andira aubletii*	rhizobial symbiosis	JX295893	-	JX275923
Fabaceae	*Calia arizonica*	rhizobial symbiosis	AY386864	-	-
Fabaceae	*Exostyles venusta*	lacking nitrogen-fixing symbiosis	-	-	AF309838.2
Fabaceae	*Harleyodendron unifoliolatum*	lacking nitrogen-fixing symbiosis	-	-	AF309837
Fabaceae	*Sweetia fruticosa*	rhizobial symbiosis	AY386911	-	-
Fabaceae	*Luetzelburgia auriculata*	lacking nitrogen-fixing symbiosis	AY553716	-	AF309834
Fabaceae	*Vatairea sp.*	lacking nitrogen-fixing symbiosis	AF270859	-	AF309836
Fabaceae	*Cladastris sinensis*	lacking nitrogen-fixing symbiosis	-	Z95551	-
Fabaceae	*Pickeringia montana*	lacking nitrogen-fixing symbiosis	AY386863	-	-
Fabaceae	*Aldina latifolia*	lacking nitrogen-fixing symbiosis	-	U74252	-

Fabaceae	*Castanospermum australe*	lacking nitrogen-fixing symbiosis	-	U74202	AF311375
Fabaceae	*Alexa canaracunensis*	rhizobial symbiosis	-	-	AF311376
Fabaceae	*Xanthocercis zambesiaca*	lacking nitrogen-fixing symbiosis	JF270996	U74189	AF311365
Fabaceae	*Angylocalyx sp.*	lacking nitrogen-fixing symbiosis	AY553715	-	AF311366
Fabaceae	*Dipteryx oleifera*	lacking nitrogen-fixing symbiosis	GQ981983	GQ981725	-
Fabaceae	*Pterodon pubescens*	lacking nitrogen-fixing symbiosis	AH009912	-	AF208895
Fabaceae	*Taralea oppositifolia*	lacking nitrogen-fixing symbiosis	-	-	AF309855
Fabaceae	*Dussia macroprophyllata*	rhizobial symbiosis	AY386903	-	-
Fabaceae	*Cordyla africana*	lacking nitrogen-fixing symbiosis	JF270724	U74204	-
Fabaceae	*Amburana cearensis*	lacking nitrogen-fixing symbiosis	AY553712	-	AF309846
Fabaceae	*Mildbraediodendron excelsum*	lacking nitrogen-fixing symbiosis	-	-	AF309847
Fabaceae	*Myroxylon balsamum*	rhizobial symbiosis	FJ151488	U74208	AF309850
Fabaceae	*Myrocarpus frondosus*	rhizobial symbiosis	AY386925	-	AF311002
Fabaceae	*Trischidium molle*	undetermined	-	-	EF466362
Fabaceae	*Cyathostegia mathewsii*	rhizobial symbiosis	HM347487	-	AF309841
Fabaceae	*Bobgunnia fistuloides*	rhizobial symbiosis	EU361885	AM234258	AF365038
Fabaceae	*Candolleodendron brachystachyum*	undetermined	-	-	EF466264
Fabaceae	*Bocoa prouacensis*	undetermined	FJ037904	FJ038032	FJ039261
Fabaceae	*Gleditsia sinensis*	rhizobial symbiosis	AY386930.2	AY904374	AY899686
Fabaceae	*Gymnocladus dioica*	lacking nitrogen-fixing symbiosis	EU361966	U74193	AY899685
Fabaceae	*Umtiza listeriana*	undetermined	EU362062	AM234237	AF365126
Fabaceae	*Arcoa gonavensis*	undetermined	EU361861	-	AY232787
Fabaceae	*Ceratonia siliqua*	lacking nitrogen-fixing symbiosis	EU361911	U74203	AF365075
Fabaceae	*Acrocarpus fraxinifolius*	lacking nitrogen-fixing symbiosis	EU361843	AY904371	AY899683
Fabaceae	*Tetrapterocarpon geayi*	undetermined	-	AY904372	AY899684
Fabaceae	*Erythrostemon gilliesii*	lacking nitrogen-fixing symbiosis	-	JN796934	EU361765
Fabaceae	*Poincianella mexicana*	lacking nitrogen-fixing symbiosis	EU361904	-	EU361772
Fabaceae	*Pomaria jamesii*	undetermined	EU362029	-	EU361830
Fabaceae	*Stahlia monosperma*	lacking nitrogen-fixing symbiosis	EU362050	-	EU361838
Fabaceae	*Balsamocarpon brevifolium*	lacking nitrogen-fixing symbiosis	EU361864	AY308524	EU361739
Fabaceae	*Hoffmannseggia glauca*	lacking nitrogen-fixing symbiosis	EU361969	AY308532	AF365069
Fabaceae	*Zuccagnia punctata*	undetermined	-	AY308547	EU361842
Fabaceae	*Haematoxylum brasiletto*	lacking nitrogen-fixing symbiosis	AY386905	AY904383	AY899696
Fabaceae	*Mezoneuron kauaiense*	undetermined	EU361903	-	EU361770
Fabaceae	*Guilandina crista*	lacking nitrogen-fixing symbiosis	EU361900	-	EU361761
Fabaceae	*Pterolobium stellatum*	lacking nitrogen-fixing symbiosis	EU362032	-	AF365073
Fabaceae	*Cordeauxia edulis*	lacking nitrogen-fixing symbiosis	EU361920	AY904378	AY899690
Fabaceae	*Tara spinosa*	lacking nitrogen-fixing symbiosis	-	-	EU361776
Fabaceae	*Stuhlmannia moavi*	undetermined	-	AY904395	AY899707
Fabaceae	*Pterogyne nitens*	lacking nitrogen-fixing symbiosis	EU362031	AY904377	AY899689
Fabaceae	*Vouacapoua macropetala*	rhizobial symbiosis	EU362063	-	AF365110
Fabaceae	*Senna alata*	lacking nitrogen-fixing symbiosis	EU362042	U74250	AF365091
Fabaceae	*Chamaecrista nictitans*	rhizobial symbiosis	EU361914	GQ248565	-
Fabaceae	*Batesia floribunda*	lacking nitrogen-fixing symbiosis	EU361869	AY904375	AY899687
Fabaceae	*Melanoxylon brauna*	rhizobial symbiosis	EU362000	AY904388	AY899700
Fabaceae	*Recordoxylon speciosum*	rhizobial symbiosis	-	AY904387	AY899699
Fabaceae	*Lemuropisum edule*	undetermined	EU361991	AY904426	AY899796
Fabaceae	*Delonix elata*	lacking nitrogen-fixing symbiosis	EU361928	AM234235	AY899731
Fabaceae	*Colvillea racemosa*	lacking nitrogen-fixing symbiosis	EU361916	AY904425	AY899739
Fabaceae	*Conzattia multiflora*	lacking nitrogen-fixing symbiosis	AY386918.2	AY904416	AY899729
Fabaceae	*Heteroflorum sp.*	undetermined	-	AY904414	AY899727
Fabaceae	*Cercidium andicola*	lacking nitrogen-fixing symbiosis	AY944552	AY904410	AY899722
Fabaceae	*Parkinsonia praecox*	lacking nitrogen-fixing symbiosis	AY944553	AY904409	AY899721
Fabaceae	*Schizolobium parahyba*	lacking nitrogen-fixing symbiosis	EU362036	AY904398	AY899711
Fabaceae	*Peltophorum pterocarpum*	lacking nitrogen-fixing symbiosis	EU362023	AM234243	AY899713
Fabaceae	*Bussea perrieri*	lacking nitrogen-fixing symbiosis	EU361896.2	-	EU361757
Fabaceae	*Campsiandra comosa*	rhizobial symbiosis	EU361908	-	EU361780

Fabaceae	*Arapatiella psilophylla*	lacking nitrogen-fixing symbiosis	EU361859	-	EU361738
Fabaceae	*Jacqueshuberia brevipes*	undetermined	EU361984	-	EU361815
Fabaceae	*Sclerolobium sp.*	rhizobial symbiosis	-	AM234242	AF365111
Fabaceae	*Tachigali sp.*	rhizobial symbiosis	EU362040	-	AF365112
Fabaceae	*Dinizia excelsa*	lacking nitrogen-fixing symbiosis	AF521827	-	AF278479
Fabaceae	*Dimorphandra conjugata*	rhizobial symbiosis	EU361934	-	AF365099
Fabaceae	*Mora gonggrijpii*	lacking nitrogen-fixing symbiosis	EU362005	-	AF365104
Fabaceae	*Burkea africana*	lacking nitrogen-fixing symbiosis	EU361895	JF265317	EU361755
Fabaceae	*Diptychandra aurantiaca*	lacking nitrogen-fixing symbiosis	EU361935	-	AF309478
Fabaceae	*Moldenhawera brasiliensis*	rhizobial symbiosis	EU362004	AY904390	AY899702
Fabaceae	*Pachyelasma tessmannii*	undetermined	EU362013	-	AF365105
Fabaceae	*Erythrophleum ivorense*	rhizobial symbiosis	EU361948	U74205	AF365102
Fabaceae	*Calpocalyx dinklagei*	undetermined	AY944551	AM234257	AF278483
Fabaceae	*Xylia torreana*	rhizobial symbiosis	JF271001	JF265660	-
Fabaceae	*Pseudoprosopis gilletii*	undetermined	AF521861	-	-
Fabaceae	*Adenanthera pavonina*	rhizobial symbiosis	AF521808	-	AF278486
Fabaceae	*Amblygonocarpus andongensis*	lacking nitrogen-fixing symbiosis	AF521812	-	AF278487
Fabaceae	*Tetrapleura tetraptera*	lacking nitrogen-fixing symbiosis	AF521865	-	AF278510
Fabaceae	*Elephantorrhiza elephantina*	rhizobial symbiosis	AF521828	JF265409	AF278484
Fabaceae	*Entada phaseoloides*	rhizobial symbiosis	EU328415	JF738904	-
Fabaceae	*Piptadeniastrum africanum*	rhizobial symbiosis	AF521857	-	AF278488
Fabaceae	*Plathymenia reticulata*	rhizobial symbiosis	AF521858	-	AF278509
Fabaceae	*Fillaeopsis discophora*	undetermined	AF521833	-	AF278508
Fabaceae	*Newtonia hildebrandtii*	lacking nitrogen-fixing symbiosis	AF521848	-	AF278502
Fabaceae	*Cylicodiscus gabunensis*	lacking nitrogen-fixing symbiosis	AF521819	-	-
Fabaceae	*Dicrostachys cinerea*	undetermined	-	-	AF522941
Fabaceae	*Alantsilodendron alluaudianum*	undetermined	AF521809	-	AF278523
Fabaceae	*Gagnebina pervilleana*	undetermined	AF521837	-	AF278528
Fabaceae	*Dichrostachys cinerea subsp. africana*	rhizobial symbiosis	JF270739	JF265387	-
Fabaceae	*Calliandropsis nervosus*	undetermined	AF521816	-	AF278520
Fabaceae	*Vachellia farnesiana*	rhizobial symbiosis	HM020715	HM850439	HM020805
Fabaceae	*Prosopsis sp.*	undetermined	EU812013	-	-
Fabaceae	*Xerocladia viridiramis*	rhizobial symbiosis	EU000438	-	-
Fabaceae	*Prosopis velutina*	rhizobial symbiosis	EU025910	JN796946	-
Fabaceae	*Indopiptadenia oudhensis*	rhizobial symbiosis	-	JQ388196	-
Fabaceae	*Desmanthus bicornutus*	rhizobial symbiosis	AF523108	-	AF522939
Fabaceae	*Kanaloa kahoolawensis*	undetermined	AF521839	-	AF278489
Fabaceae	*Schleinitzia insularum*	rhizobial symbiosis	AF521862	-	AF278491
Fabaceae	*Leucaena leucocephala*	rhizobial symbiosis	AY574102	GU135204	AF522942
Fabaceae	*Mimozyganthus carinatus*	rhizobial symbiosis	AY944556	-	DQ344570
Fabaceae	*Piptadeniopsis lomentifera*	undetermined	AY944559	-	-
Fabaceae	*Prosopidastrum angusticarpum*	undetermined	EF165252	-	-
Fabaceae	*Neptunia monosperma*	rhizobial symbiosis	AF523090	-	AF522944
Fabaceae	*Anadenanthera colubrina*	rhizobial symbiosis	EU812064	-	AF278481
Fabaceae	*Parkia multijuga*	lacking nitrogen-fixing symbiosis	EU362018	AM234251	AF365050
Fabaceae	*Stryphnodendron moricolor*	rhizobial symbiosis	FJ037919	FJ038054	-
Fabaceae	*Pseudopiptadenia suaveolens*	rhizobial symbiosis	DQ790637	FJ038053	FJ039233
Fabaceae	*Microlobius foetidus*	rhizobial symbiosis	AF523095	-	AF522960
Fabaceae	*Parapiptadenia rigida*	rhizobial symbiosis	AF521849	-	AF278505
Fabaceae	*Adenopodia spicata*	lacking nitrogen-fixing symbiosis	JF270628	JF265272	-
Fabaceae	*Piptadenia flava*	rhizobial symbiosis	DQ790616	-	AY574110
Fabaceae	*Acacia schweinfurthii*	lacking nitrogen-fixing symbiosis	AF523101	JF265257	AF522979
Fabaceae	*Mariosousa dolichostachya*	rhizobial symbiosis	EU812056	-	-
Fabaceae	*Senegalia visco*	rhizobial symbiosis	EU812059	-	HM020824
Fabaceae	*Acaciella tequilana*	rhizobial symbiosis	EU812044	-	HM020831
Fabaceae	*Zapoteca tetragona*	lacking nitrogen-fixing symbiosis	AF523097	-	AF522966
Fabaceae	*Faidherbia albida*	rhizobial symbiosis	AF523081	JF265429	AF522954

Fabaceae	*Cedrelinga cateniformis*	rhizobial symbiosis	AF521818	AM234256	AF278511
Fabaceae	*Pithecellobium clypearia*	rhizobial symbiosis	HQ415281	GQ436357	-
Fabaceae	*Pararchidendron pruinosum*	undetermined	AF274127	-	AF522961
Fabaceae	*Archidendron hirsutum*	rhizobial symbiosis	EU361860	AM234253	AF365042
Fabaceae	*Samanea saman*	rhizobial symbiosis	AF523073	-	AF522965
Fabaceae	*Paraserianthes lophantha*	rhizobial symbiosis	AF274128	Z70148	AF522962
Fabaceae	*Abarema macradenia*	rhizobial symbiosis	GQ981925	GQ981652	-
Fabaceae	*Cathormion umbellatum*	rhizobial symbiosis	AF274122	-	AF522949
Fabaceae	*Chloroleucon mangense*	rhizobial symbiosis	AF523072	-	AF522950
Fabaceae	*Ebenopsis ebano*	rhizobial symbiosis	AF274123	-	AF522951
Fabaceae	*Havardia albicans*	rhizobial symbiosis	AF523085	-	AF522956
Fabaceae	*Cojoba rufescens*	rhizobial symbiosis	GQ981971	GQ981709	-
Fabaceae	*Lysiloma tergeminum*	rhizobial symbiosis	AF523089	-	AF522959
Fabaceae	*Albizia kalkora*	rhizobial symbiosis	AF523083	HQ427141	AF522945
Fabaceae	*Calliandra juzepczukii*	rhizobial symbiosis	EU812063	-	AF522948
Fabaceae	*Pseudosamanea guachapele*	rhizobial symbiosis	AF523079	-	AF522964
Fabaceae	*Enterolobium schomburgkii*	rhizobial symbiosis	GQ981984	GQ981727	-
Fabaceae	*Zygia racemosa*	rhizobial symbiosis	FJ037923	FJ038060	FJ039293
Fabaceae	*Inga edulis*	rhizobial symbiosis	AF523078	FJ173737	AF522957
Fabaceae	*Eligmocarpus cynometroides*	undetermined	EU361939	-	EU361801
Fabaceae	*Baudouinia sp.*	undetermined	EU361872	-	EU361741
Fabaceae	*Mendoravia dumaziana*	undetermined	EU362001	-	EU361823
Fabaceae	*Labichea punctata*	undetermined	EU361989	-	AF365076
Fabaceae	*Petalostylis labicheoides*	lacking nitrogen-fixing symbiosis	EU362024	AF308719	AF365077
Fabaceae	*Storckiella australiensis*	undetermined	EU362052	AM234249	AF365078
Fabaceae	*Koompassia excelsa*	lacking nitrogen-fixing symbiosis	EU361988	-	AF365029
Fabaceae	*Martiodendron parviflorum*	lacking nitrogen-fixing symbiosis	EU361999	-	AF365086
Fabaceae	*Zenia insignis*	undetermined	EU362065	AF308722	AF365085
Fabaceae	*Dialium guianense*	lacking nitrogen-fixing symbiosis	EU361930	AM234245	AF365079
Fabaceae	*Dicorynia guianensis*	lacking nitrogen-fixing symbiosis	EU361931	FJ038034	FJ039275
Fabaceae	*Distemonanthus benthamianus*	lacking nitrogen-fixing symbiosis	EU361936	-	AF365084
Fabaceae	*Piliostigma thonningii*	lacking nitrogen-fixing symbiosis	-	JF265551	FJ801153
Fabaceae	*Phanera outimouta*	lacking nitrogen-fixing symbiosis	EU361877	-	EU361745
Fabaceae	*Lysiphyllum gilvum*	lacking nitrogen-fixing symbiosis	EU361876	-	-
Fabaceae	*Barklya syringifolia*	undetermined	EU361878	-	-
Fabaceae	*Lasiobema championii var. championii*	undetermined	EU361873	-	-
Fabaceae	*Tylosema fassoglense*	lacking nitrogen-fixing symbiosis	EU361874	-	EU361743
Fabaceae	*Gigasiphon macrosiphon*	undetermined	EU361953	-	EU361810
Fabaceae	*Bauhinia galpinii*	lacking nitrogen-fixing symbiosis	EU361875	AM234262	-
Fabaceae	*Brenierea insignis*	undetermined	EU361889	AM234269	AF365060
Fabaceae	*Adenolobus garipensis*	undetermined	EU361844	AM234268	-
Fabaceae	*Neoapaloxylon madagascariense*	undetermined	-	-	AY958518
Fabaceae	*Goniorrhachis marginata*	lacking nitrogen-fixing symbiosis	EU361959	AM234232	AF365185
Fabaceae	*Barnebydendron riedelii*	rhizobial symbiosis	EU361868	-	AF365209
Fabaceae	*Schotia afra*	lacking nitrogen-fixing symbiosis	EU362037	AM235016	AF365122
Fabaceae	*Pseudosindora palustris*	undetermined	-	-	AY958484
Fabaceae	*Sindoropsis le-testui*	undetermined	EU362049	-	AF365189
Fabaceae	*Tessmannia africana*	lacking nitrogen-fixing symbiosis	EU362057	-	AF365191
Fabaceae	*Sindora klaineana*	lacking nitrogen-fixing symbiosis	EU362045	-	AY187228
Fabaceae	*Copaifera officinalis*	lacking nitrogen-fixing symbiosis	EU361918	-	AY958460
Fabaceae	*Baikiaea insignis*	lacking nitrogen-fixing symbiosis	EU361863	-	AF365179
Fabaceae	*Hylodendron gabunense*	lacking nitrogen-fixing symbiosis	EU361971	-	AF365186
Fabaceae	*Gilletiodendron pierreanum*	undetermined	EU361957	-	AF365184
Fabaceae	*Eperua falcata*	lacking nitrogen-fixing symbiosis	EU361945	FJ038036	FJ039125
Fabaceae	*Eurypetalum tessmannii*	undetermined	EU361950	-	AF365137
Fabaceae	*Augouardia letestui*	undetermined	EU361862	-	AF365164
Fabaceae	*Stemonocoleus micranthus*	undetermined	EU362051	-	AF365178

Fabaceae	*Peltogyne confertiflora*	lacking nitrogen-fixing symbiosis	EU362021	AF308718	AF365163
Fabaceae	*Guibourtia conjugata*	lacking nitrogen-fixing symbiosis	JF270804	JF265457	-
Fabaceae	*Hymenaea courbaril*	rhizobial symbiosis	EU361972	JQ591780	AF365160
Fabaceae	*Daniellia klainei*	lacking nitrogen-fixing symbiosis	EU361927	-	AF365135
Fabaceae	*Brandzeia filicifolia*	undetermined	EU361870	-	AY187226
Fabaceae	*Hardwickia binata*	lacking nitrogen-fixing symbiosis	EU361967	-	AY187227
Fabaceae	*Colophospermum mopane*	lacking nitrogen-fixing symbiosis	EU361915	JF265343	AF365165
Fabaceae	*Kingiodendron pinnatum*	lacking nitrogen-fixing symbiosis	EU361987	-	AF365169
Fabaceae	*Oxystigma msoo*	undetermined	EU362011	-	AF365168
Fabaceae	*Prioria copaifera*	rhizobial symbiosis	EU362030	-	AF365171
Fabaceae	*Gossweilerodendron balsamiferum*	rhizobial symbiosis	EU361960	-	AF365166
Fabaceae	*Endertia spectabilis*	undetermined	EU361943	-	AF365136
Fabaceae	*Lysidice rhodostegia*	rhizobial symbiosis	EU361995	-	AF365152
Fabaceae	*Saraca palembanica*	rhizobial symbiosis	EU362035	AM234238	AF365157
Fabaceae	*Afzelia quanzensis*	rhizobial symbiosis	EU361848	JF265273	AF365130
Fabaceae	*Intsia bijuga*	rhizobial symbiosis	EU361981	JF738765	AF365149
Fabaceae	*Brodriguesia santosii*	lacking nitrogen-fixing symbiosis	EU361890	-	EU361750
Fabaceae	*Polystemonanthus dinklagei*	undetermined	EU362028	-	AF365226
Fabaceae	*Dicymbe altsonii*	rhizobial symbiosis	EU361932	-	AF365217
Fabaceae	*Tamarindus indica*	rhizobial symbiosis	EU362056	AB378728	AF365206
Fabaceae	*Hymenostegia ngounyensis*	lacking nitrogen-fixing symbiosis	EU361977	-	AF365142
Fabaceae	*Scorodophloeus zenkeri*	lacking nitrogen-fixing symbiosis	EU362041	-	AF365125
Fabaceae	*Plagiosiphon sp.*	undetermined	EU361926	-	EU361789
Fabaceae	*Neochevalierodendron stephanii*	undetermined	EU362006	-	AF365151
Fabaceae	*Crudia gabonensis*	rhizobial symbiosis	EU361922	AM234230	AF365172
Fabaceae	*Lebruniodendron leptanthum*	undetermined	-	-	EU361817
Fabaceae	*Cynometra mannii*	rhizobial symbiosis	EU361925	AM234231	AF365114
Fabaceae	*Maniltoa gemmipara*	rhizobial symbiosis	EU361998	-	AF365120
Fabaceae	*Zenkerella citrina*	lacking nitrogen-fixing symbiosis	EU362066	-	AF365127
Fabaceae	*Normandiodendron becquaertii*	undetermined	EU362007	-	AF365119
Fabaceae	*Humboldtia vahliana*	rhizobial symbiosis	EU361970	-	AF365212
Fabaceae	*Talbotiella gentii*	undetermined	EU362055	-	AF365159
Fabaceae	*Leonardoxa africana*	undetermined	EU361992	-	AF365118
Fabaceae	*Loesenera kalantha*	lacking nitrogen-fixing symbiosis	EU361994	-	AF233472
Fabaceae	*Paloue induta*	undetermined	EU362015	-	FJ817578
Fabaceae	*Elizabetha paraensis*	rhizobial symbiosis	EU361941	-	AF365208
Fabaceae	*Browneopsis ucayalina*	undetermined	EU361894	AM234233	AF365199
Fabaceae	*Paloveopsis emarginata*	undetermined	-	-	FJ817570
Fabaceae	*Ecuadendron acosta-solisianum*	undetermined	EU361938	-	AF365207
Fabaceae	*Brownea sp.*	rhizobial symbiosis	AY386932	U74186	EU361754
Fabaceae	*Heterostemon conjugatus*	lacking nitrogen-fixing symbiosis	EU361968	-	EU598689
Fabaceae	*Macrolobium bifolium*	rhizobial symbiosis	EU361996	-	AF365200
Fabaceae	*Cryptosepalum staudtii*	lacking nitrogen-fixing symbiosis	EU361923	-	AF365258
Fabaceae	*Paramacrolobium coeruleum*	lacking nitrogen-fixing symbiosis	EU362017	-	AF365242
Fabaceae	*Gilbertiodendron klainei*	rhizobial symbiosis	EU361955	-	EU361811
Fabaceae	*Pellegriniodendron diphyllum*	lacking nitrogen-fixing symbiosis	EU362020	-	AF365243
Fabaceae	*Librevillea klainei*	undetermined	EU361993	-	AF365262
Fabaceae	*Didelotia africana*	lacking nitrogen-fixing symbiosis	EU361933	-	AF365260
Fabaceae	*Anthonotha macrophylla*	lacking nitrogen-fixing symbiosis	EU361853	-	AF365234
Fabaceae	*Oddoniodendron micranthum*	undetermined	EU362008	-	AF365225
Fabaceae	*Berlinia congolensis*	lacking nitrogen-fixing symbiosis	EU361881	-	AF365216
Fabaceae	*Englerodendron usambarense*	undetermined	EU361944	-	AF365218
Fabaceae	*Isoberlinia scheffleri*	lacking nitrogen-fixing symbiosis	EU361983	AM234240	AF365221
Fabaceae	*Microberlinia brazzavillensis*	lacking nitrogen-fixing symbiosis	EU362003	-	AF365222
Fabaceae	*Brachystegia boehmii*	rhizobial symbiosis	EU361886	-	EU361749
Fabaceae	*Icuria dunensis*	undetermined	EU361979	-	AF365232
Fabaceae	*Aphanocalyx cynometroides*	undetermined	EU361855	AM234241	AF365244

Fabaceae	*Julbernardia brieyi*	lacking nitrogen-fixing symbiosis	EU361985	-	AF365264
Fabaceae	*Tetraberlinia bifoliolata*	lacking nitrogen-fixing symbiosis	EU362060	-	AF365227
Fabaceae	*Bikinia durandii*	undetermined	EU361883	-	AY116896
Fagaceae	*Fagus crenata**	lacking nitrogen-fixing symbiosis	AB046496	AB060567	AF533693
Fagaceae	*Lithocarpus henryi**	lacking nitrogen-fixing symbiosis	EF057119	AY147097	AY147086
Fagaceae	*Quercus robur**	lacking nitrogen-fixing symbiosis	AJ491718	AB125025	AF268937
Fagaceae	*Castanopsis fissa*	lacking nitrogen-fixing symbiosis	FJ185053	JF941177	EF057141
Fagaceae	*Castanea crenata*	lacking nitrogen-fixing symbiosis	AB107636	AB060565	AF344181
Fagaceae	*Chrysolepis sempervirens*	lacking nitrogen-fixing symbiosis	U92863	AF206750	-
Fagaceae	*Formanodendron doichangensis*	lacking nitrogen-fixing symbiosis	FJ185046	-	-
Fagaceae	*Trigonobalanus verticillata*	lacking nitrogen-fixing symbiosis	AB084770	AB084768	AY147085
Fagaceae	*Colombobalanus excelsa*	lacking nitrogen-fixing symbiosis	AY042456	-	-
Juglandaceae	*Alfaroa williamsii**	lacking nitrogen-fixing symbiosis	U92849	-	-
Juglandaceae	*Annamocarya sinensis**	lacking nitrogen-fixing symbiosis	AY263919	AY263935	AY147080
Juglandaceae	*Carya ovata**	lacking nitrogen-fixing symbiosis	U92850	AY263931	AY147074
Juglandaceae	*Engelhardia fenzelii**	lacking nitrogen-fixing symbiosis	AY147099	AY147095	AY147076
Juglandaceae	*Juglans mandshurica**	lacking nitrogen-fixing symbiosis	AF118033	AY263932	AY147077
Juglandaceae	*Alfaropsis roxburghiana*	lacking nitrogen-fixing symbiosis	HQ415289	-	-
Juglandaceae	*Platycarya strobilacea*	lacking nitrogen-fixing symbiosis	AY147100	AY263933	AY147078
Juglandaceae	*Cyclocarya paliurus*	lacking nitrogen-fixing symbiosis	AY147098	AY147094	AY147075
Juglandaceae	*Pterocarya hupehensis*	lacking nitrogen-fixing symbiosis	AY263918	AY263934	AY147079
Moraceae	*Artocarpus camansi**	lacking nitrogen-fixing symbiosis	-	JF739111	FJ917088
Moraceae	*Bagassa guianensis**	lacking nitrogen-fixing symbiosis	FJ037931	FJ038118	FJ917066
Moraceae	*Brosimum alicastrum**	lacking nitrogen-fixing symbiosis	GQ981947	AF500346	AF501601
Moraceae	*Broussonetia papyrifera**	lacking nitrogen-fixing symbiosis	AF345326	AF500347	AF501602
Moraceae	*Ficus pumila**	lacking nitrogen-fixing symbiosis	HM851109	AF500352	AF501606
Moraceae	*Helicostylis tomentosa**	lacking nitrogen-fixing symbiosis	FJ514761	FJ038122	FJ039339
Moraceae	*Hullettia dumosa**	lacking nitrogen-fixing symbiosis	-	-	FJ917070
Moraceae	*Maclura tricuspidata**	lacking nitrogen-fixing symbiosis	JF317421	JF317480	JN006418
Moraceae	*Morus rubra**	lacking nitrogen-fixing symbiosis	GU145566	U06812	HM747181
Moraceae	*Parartocarpus venenosus**	lacking nitrogen-fixing symbiosis	-	-	FJ917110
Moraceae	*Prainea limpato subsp. papuana**	lacking nitrogen-fixing symbiosis	-	JF738760	FJ917072
Moraceae	*Streblus indicus**	lacking nitrogen-fixing symbiosis	GQ434235	GQ436641	-
Moraceae	*Poulsenia armata*	lacking nitrogen-fixing symbiosis	-	GQ981838	-
Moraceae	*Perebea xanthochyma*	lacking nitrogen-fixing symbiosis	GQ982060	GQ981827	-
Moraceae	*Pseudolmedia spuria*	lacking nitrogen-fixing symbiosis	HM446734	HM446858	-
Moraceae	*Naucleopsis guianensis*	lacking nitrogen-fixing symbiosis	-	GQ428596	FJ039165
Moraceae	*Antiaris toxicaria*	lacking nitrogen-fixing symbiosis	GQ434236	GQ436642	-
Moraceae	*Maquira calophylla*	lacking nitrogen-fixing symbiosis	FJ514665	GQ428592	FJ039319
Moraceae	*Castilla elastica*	lacking nitrogen-fixing symbiosis	-	AF500348	FJ917062
Moraceae	*Dorstenia mannii*	lacking nitrogen-fixing symbiosis	-	AF500349	AF501604
Moraceae	*Treculia obovoidea*	lacking nitrogen-fixing symbiosis	-	-	FJ917069
Moraceae	*Trymatococcus oligandrus*	lacking nitrogen-fixing symbiosis	FJ037932	FJ038126	FJ039166
Moraceae	*Batocarpus costaricensis*	lacking nitrogen-fixing symbiosis	-	-	FJ917065
Moraceae	*Clarisia biflora*	lacking nitrogen-fixing symbiosis	-	-	FJ917067
Moraceae	*Milicia cf.*	lacking nitrogen-fixing symbiosis	-	-	AF278786
Moraceae	*Sorocea affinis*	lacking nitrogen-fixing symbiosis	GQ982100	GQ981880	HM747195
Moraceae	*Trophis racemosa*	lacking nitrogen-fixing symbiosis	GQ982120	GQ981908	HM747194
Myricaceae	*Canacomyrica monticola**	lacking nitrogen-fixing symbiosis	-	DQ310504	-
Myricaceae	*Comptonia peregrina**	actinorhizal symbiosis	U92856	DQ310505	-
Myricaceae	*Myrica gale**	actinorhizal symbiosis	AY191715	X69530	GQ245142
Nothofagaceae	*Nothofagus antarctica**	lacking nitrogen-fixing symbiosis	AY263924	AY263939	AY147091
Polygalaceae	*Polygala senega**	lacking nitrogen-fixing symbiosis	EU604034	AM234189	GQ889195
Polygalaceae	*Xanthophyllum sp.**	lacking nitrogen-fixing symbiosis	EU604044	AJ235799	-
Polygalaceae	*Moutabea aculeata*	lacking nitrogen-fixing symbiosis	-	AM234169	-
Polygalaceae	*Barnhartia floribunda*	lacking nitrogen-fixing symbiosis	-	AM234168	-
Polygalaceae	*Eriandra fragrans*	lacking nitrogen-fixing symbiosis	EU604051	AM234170	GQ889070
Polygalaceae	*Carpolobia alba*	lacking nitrogen-fixing symbiosis	EU604053	AM234176	GQ889064

Polygalaceae	*Atroxima afzeliana*	lacking nitrogen-fixing symbiosis	EU604049	AM234175	GQ889056
Polygalaceae	*Bredemeyera floribunda*	lacking nitrogen-fixing symbiosis	EU596520	EU644699	GQ889062
Polygalaceae	*Badiera fuertesii*	lacking nitrogen-fixing symbiosis	-	-	GQ889058
Polygalaceae	*Comesperma esulifolium*	lacking nitrogen-fixing symbiosis	EU596516	AM234179	GQ889068
Polygalaceae	*Monnina xalapensis*	lacking nitrogen-fixing symbiosis	EU604047	AM234184	GQ889088
Polygalaceae	*Phlebotaenia cuneata*	lacking nitrogen-fixing symbiosis	-	-	GQ889094
Polygalaceae	*Securidaca retusa*	lacking nitrogen-fixing symbiosis	EU604029	EU644681	GQ889230
Polygalaceae	*Acanthocladus guayaquilensis*	lacking nitrogen-fixing symbiosis	-	AM234190	-
Polygalaceae	*Salomonia cantoniensis*	lacking nitrogen-fixing symbiosis	-	-	GQ889225
Polygalaceae	*Heterosamara tatarinowii*	lacking nitrogen-fixing symbiosis	-	AM234208	-
Polygalaceae	*Muraltia thunbergii*	lacking nitrogen-fixing symbiosis	AM889730	GQ248650	-
Polygalaceae	*Nylandtia scoparia*	lacking nitrogen-fixing symbiosis	-	AJ829701	GQ889093
Quillajaceae	*Quillaja saponaria**	lacking nitrogen-fixing symbiosis	AY386843	U06822	-
Rhamnaceae	*Adolphia infesta**	actinorhizal symbiosis	-	AJ390055	-
Rhamnaceae	*Ampelozizyphus amazonicus**	lacking nitrogen-fixing symbiosis	-	AJ390037	-
Rhamnaceae	*Bathiorhamnus cryptophorous**	lacking nitrogen-fixing symbiosis	-	AJ390036	-
Rhamnaceae	*Berchemia discolor**	lacking nitrogen-fixing symbiosis	JF270655	AJ225786	-
Rhamnaceae	*Ceanothus sanguineus**	actinorhizal symbiosis	AF049815	U06795	-
Rhamnaceae	*Colletia ulcinia**	actinorhizal symbiosis	-	U59819	-
Rhamnaceae	*Colubrina asiatica**	lacking nitrogen-fixing symbiosis	GU135023	AJ390047	-
Rhamnaceae	*Condalia microphylla**	lacking nitrogen-fixing symbiosis	-	AJ390032	-
Rhamnaceae	*Crumenaria erecta**	lacking nitrogen-fixing symbiosis	-	AJ390042	-
Rhamnaceae	*Cryptandra myriantha**	lacking nitrogen-fixing symbiosis	-	AJ390060	-
Rhamnaceae	*Discaria chacaye**	actinorhizal symbiosis	-	AF307911	AY460415
Rhamnaceae	*Doerpfeldia cubensis**	lacking nitrogen-fixing symbiosis	-	AJ390038	-
Rhamnaceae	*Emmenosperma alphitonioides**	lacking nitrogen-fixing symbiosis	-	AJ390048	-
Rhamnaceae	*Frangula alnus**	lacking nitrogen-fixing symbiosis	AY257532	AJ390026	HQ323903
Rhamnaceae	*Gouania mauritiana**	lacking nitrogen-fixing symbiosis	JF317427	AJ390040	-
Rhamnaceae	*Granitites intangendus**	lacking nitrogen-fixing symbiosis	-	AJ306539	AJ306540
Rhamnaceae	*Hovenia trichocarpa var. robusta**	lacking nitrogen-fixing symbiosis	JF317429	JF317489	-
Rhamnaceae	*Karwinskia humboldtiana**	lacking nitrogen-fixing symbiosis	-	AJ390031	-
Rhamnaceae	*Kentrothamnus weddellianus**	actinorhizal symbiosis	-	-	AY460422
Rhamnaceae	*Krugiodendron ferreum**	lacking nitrogen-fixing symbiosis	-	AJ390028	-
Rhamnaceae	*Lasiodiscus mildbraedii**	lacking nitrogen-fixing symbiosis	-	AJ390050	-
Rhamnaceae	*Maesopsis eminii**	lacking nitrogen-fixing symbiosis	-	AJ390034	-
Rhamnaceae	*Noltea africana**	lacking nitrogen-fixing symbiosis	-	AJ390054	AY460407
Rhamnaceae	*Paliurus spina-christi**	lacking nitrogen-fixing symbiosis	-	AJ390051	-
Rhamnaceae	*Phylica arborea**	lacking nitrogen-fixing symbiosis	GQ248177	GQ248666	AF327603
Rhamnaceae	*Pomaderris rugosa**	lacking nitrogen-fixing symbiosis	-	AJ390063	-
Rhamnaceae	*Retanilla patagonica**	actinorhizal symbiosis	-	-	AY460424
Rhamnaceae	*Reynosia uncinata**	lacking nitrogen-fixing symbiosis	-	AJ390029	-
Rhamnaceae	*Rhamnella franguloides**	lacking nitrogen-fixing symbiosis	-	AJ390027	-
Rhamnaceae	*Rhamnidium elaeocarpum**	lacking nitrogen-fixing symbiosis	-	AJ390030	-
Rhamnaceae	*Rhamnus cathartica**	lacking nitrogen-fixing symbiosis	AY257533	L13189.2	-
Rhamnaceae	*Sageretia thea**	lacking nitrogen-fixing symbiosis	-	AJ225785	-
Rhamnaceae	*Schistocarpaea johnsonii**	lacking nitrogen-fixing symbiosis	-	AJ390046	-
Rhamnaceae	*Scutia buxifolia**	lacking nitrogen-fixing symbiosis	-	AJ390033	-
Rhamnaceae	*Siegfriedia darwinioides**	lacking nitrogen-fixing symbiosis	-	AJ390064	-
Rhamnaceae	*Spyridium globulosum**	lacking nitrogen-fixing symbiosis		AJ390058	EF528529
Rhamnaceae	*Stenanthemum pomaderroides**	lacking nitrogen-fixing symbiosis	-	AJ390057	-
Rhamnaceae	*Trevoa trinervis**	actinorhizal symbiosis	-	AJ390056	-
Rhamnaceae	*Trymalium floribundum**	lacking nitrogen-fixing symbiosis	-	AJ390062	-
Rhamnaceae	*Ventilago viminalis**	lacking nitrogen-fixing symbiosis	-	AJ390035	-
Rhamnaceae	*Alphitonia aff.*	lacking nitrogen-fixing symbiosis	-	AJ390049	-
Rhamnaceae	*Trichocephalus stipularis*	lacking nitrogen-fixing symbiosis	-	AM235105	-
Rhamnaceae	*Nesiota elliptica*	lacking nitrogen-fixing symbiosis	-	AJ225783	-
Rhamnaceae	*Ziziphus jujuba*	lacking nitrogen-fixing symbiosis	GQ434248	GQ436666	-

Rhamnaceae	*Helinus integrifolius*	lacking nitrogen-fixing symbiosis	JF270816	AJ390044	-
Rhamnaceae	*Pleuranthodes hillebrandii*	lacking nitrogen-fixing symbiosis	-	AJ390045	-
Rhamnaceae	*Reissekia smilacina*	lacking nitrogen-fixing symbiosis	-	AJ390041	-
Rhoipteleaceae	*Rhoiptelea chiliantha**	lacking nitrogen-fixing symbiosis	U92852	AF017687	AY147081
Rosaceae	*Adenostoma fasciculatum**	lacking nitrogen-fixing symbiosis	AF288093	-	AF348535
Rosaceae	*Agrimonia eupatoria**	lacking nitrogen-fixing symbiosis	JN893958	HM849744	GQ384718
Rosaceae	*Armeniaca zhengheensis**	lacking nitrogen-fixing symbiosis	JF955833	-	-
Rosaceae	*Cercocarpus betuloides**	actinorhizal symbiosis	AF288095	-	AF348537
Rosaceae	*Chamaebatia foliolosa**	actinorhizal symbiosis	AF288096	-	AF348538
Rosaceae	*Chamaebatiaria millefolium**	lacking nitrogen-fixing symbiosis	AF288097	U06797	AF348539
Rosaceae	*Comarum palustre**	lacking nitrogen-fixing symbiosis	JN896178	JN890807	GQ244816
Rosaceae	*Cowania stansburiana**	actinorhizal symbiosis	-	U59817	-
Rosaceae	*Crataegus monogyna**	lacking nitrogen-fixing symbiosis	AF288099	FN689370	AF348541
Rosaceae	*Dasiphora fruticosa**	lacking nitrogen-fixing symbiosis	AB458590	GQ436580	GQ245321
Rosaceae	*Dryas octopetala**	actinorhizal symbiosis	JF317424	JF317483	DQ860555
Rosaceae	*Duchesnea indica**	lacking nitrogen-fixing symbiosis	HM850689	HM850286	-
Rosaceae	*Fallugia paradoxa**	lacking nitrogen-fixing symbiosis	AF288101	U06802	AF348543
Rosaceae	*Filipendula vulgaris**	lacking nitrogen-fixing symbiosis	JN894238	JN891051	-
Rosaceae	*Geum rivale**	lacking nitrogen-fixing symbiosis	JN895978	JN893446	HM590295
Rosaceae	*Gillenia trifoliata**	lacking nitrogen-fixing symbiosis	AF288104	JQ391262	-
Rosaceae	*Holodiscus discolor**	lacking nitrogen-fixing symbiosis	AF288105	U06807	AF348546
Rosaceae	*Lindleya mespiloides**	lacking nitrogen-fixing symbiosis	DQ860448	U06810	DQ863220
Rosaceae	*Lyonothamnus floribundus**	lacking nitrogen-fixing symbiosis	AF288107	U06811	AF348548
Rosaceae	*Maddenia hypoleuca**	lacking nitrogen-fixing symbiosis	DQ851228	-	-
Rosaceae	*Neillia thibetica**	lacking nitrogen-fixing symbiosis	JF317430	JF317490	AF487227
Rosaceae	*Neviusia alabamensis**	lacking nitrogen-fixing symbiosis	AF288109	U06815	AF348550
Rosaceae	*Osmaronia cerasiformis**	lacking nitrogen-fixing symbiosis	-	U06816	-
Rosaceae	*Physocarpus insularis**	lacking nitrogen-fixing symbiosis	GU217789	GU217791	GU217792
Rosaceae	*Porteranthus trifoliatus**	lacking nitrogen-fixing symbiosis	-	-	AF348555
Rosaceae	*Potentilla anserina**	lacking nitrogen-fixing symbiosis	AF288113	JN893482	AF348556
Rosaceae	*Prinsepia sinensis**	lacking nitrogen-fixing symbiosis	AF288114	GQ436572	AF348558
Rosaceae	*Prunus laurocerasus**	lacking nitrogen-fixing symbiosis	AF288116	U06809	AF348559
Rosaceae	*Purshia tridentata**	actinorhizal symbiosis	AF288119	U06821	AF348562
Rosaceae	*Rhodotypos scandens**	lacking nitrogen-fixing symbiosis	AB073688	U06823	AF348566
Rosaceae	*Rosa woodsii**	lacking nitrogen-fixing symbiosis	EU025926	U06824	DQ778893
Rosaceae	*Rubus chamaemorus**	lacking nitrogen-fixing symbiosis	AY366358	JN891055	DQ860604
Rosaceae	*Sanguisorba officinalis**	lacking nitrogen-fixing symbiosis	AB073696	AY395560	AY634774
Rosaceae	*Sorbaria sorbifolia**	lacking nitrogen-fixing symbiosis	AF288125	GQ436611	AF348569
Rosaceae	*Sorbus aucuparia**	lacking nitrogen-fixing symbiosis	JN896162	HQ590284	AF327596
Rosaceae	*Stephanandra tanakae**	lacking nitrogen-fixing symbiosis	AF487219	DQ250748	AF487238
Rosaceae	*Vauquelinia californica**	lacking nitrogen-fixing symbiosis	DQ860449	JQ391403	AF348573
Rosaceae	*Waldsteinia fragarioides**	lacking nitrogen-fixing symbiosis	-	U06830	-
Rosaceae	*Aria alnifolia*	lacking nitrogen-fixing symbiosis	DQ860451	-	DQ863223
Rosaceae	*Heteromeles arbutifolia*	lacking nitrogen-fixing symbiosis	DQ860464	JQ391339	DQ863236
Rosaceae	*Cotoneaster pannosus*	lacking nitrogen-fixing symbiosis	AF288098	JQ391291	AF348540
Rosaceae	*Cormus domestica*	lacking nitrogen-fixing symbiosis	DQ860456	-	DQ863228
Rosaceae	*Pyrus communis*	lacking nitrogen-fixing symbiosis	DQ860473	JQ391382	DQ863245
Rosaceae	*Eriobotrya japonica*	lacking nitrogen-fixing symbiosis	DQ860462	U06800	DQ863234
Rosaceae	*Rhaphiolepis indica*	lacking nitrogen-fixing symbiosis	DQ860474	JQ391406	DQ863246
Rosaceae	*Osteomeles schwerinae*	lacking nitrogen-fixing symbiosis	DQ860468	-	DQ863240
Rosaceae	*Pyracantha coccinea*	lacking nitrogen-fixing symbiosis	DQ860472	JQ391393	DQ863244
Rosaceae	*Pseudocydonia sinensis*	lacking nitrogen-fixing symbiosis	-	-	DQ863243
Rosaceae	*Cydonia oblonga*	lacking nitrogen-fixing symbiosis	DQ860459	JQ391334	DQ863231
Rosaceae	*Torminalis clusii*	lacking nitrogen-fixing symbiosis	DQ860477	-	DQ863249
Rosaceae	*Chamaemespilus alpina*	lacking nitrogen-fixing symbiosis	DQ860455	-	DQ863227
Rosaceae	*Photinia parvifolia*	lacking nitrogen-fixing symbiosis	HQ427355	HQ427206	-
Rosaceae	*Chaenomeles cathayensis*	lacking nitrogen-fixing symbiosis	DQ860453	JQ391326	DQ863225
Rosaceae	*Dichotomanthes tristaniicarpa*	lacking nitrogen-fixing symbiosis	DQ860460	JQ391335	DQ863232

Rosaceae	*Docynia delavayi*	lacking nitrogen-fixing symbiosis	GQ434214	JQ391267	-
Rosaceae	*Malus sargentii*	lacking nitrogen-fixing symbiosis	DQ860466	JQ391365	DQ863238
Rosaceae	*Docyniopsis tschonoskii*	lacking nitrogen-fixing symbiosis	DQ860461	-	DQ863233
Rosaceae	*Chamaemeles coriacea*	lacking nitrogen-fixing symbiosis	DQ860454	-	DQ863226
Rosaceae	*Amelanchier bartramiana*	lacking nitrogen-fixing symbiosis	DQ860450	JQ391255	DQ863222
Rosaceae	*Aronia melanocarpa*	lacking nitrogen-fixing symbiosis	-	JQ391240	-
Rosaceae	*Stranvaesia davidiana*	lacking nitrogen-fixing symbiosis	DQ860476	JQ391397	DQ863248
Rosaceae	*Peraphyllum ramosissimum*	lacking nitrogen-fixing symbiosis	DQ860469	JQ391401	DQ863241
Rosaceae	*Malacomeles denticulata*	lacking nitrogen-fixing symbiosis	DQ860465	-	DQ863237
Rosaceae	*Mespilus germanica*	lacking nitrogen-fixing symbiosis	DQ860467	-	DQ863239
Rosaceae	*Kageneckia angustifolia*	lacking nitrogen-fixing symbiosis	DQ860447	U06808	DQ863219
Rosaceae	*Pygeum topengii*	lacking nitrogen-fixing symbiosis	DQ851230	-	-
Rosaceae	*Luetkea pectinata*	lacking nitrogen-fixing symbiosis	DQ851227	DQ250750	-
Rosaceae	*Aruncus dioicus*	lacking nitrogen-fixing symbiosis	AF288094	U06794	AF348536
Rosaceae	*Pentactina rupicola*	lacking nitrogen-fixing symbiosis	NC 016921	NC 016921	EU595410
Rosaceae	*Spiraea mongolica var. mongolica*	lacking nitrogen-fixing symbiosis	GQ434194	GQ436586	-
Rosaceae	*Kelseya uniflora*	lacking nitrogen-fixing symbiosis	DQ851226	-	-
Rosaceae	*Petrophytum caespitosum*	lacking nitrogen-fixing symbiosis	EU025920	-	-
Rosaceae	*Petrophyton caespitosum*	lacking nitrogen-fixing symbiosis	DQ851229	-	-
Rosaceae	*Exochorda racemosa*	lacking nitrogen-fixing symbiosis	AF288100	-	AF348542
Rosaceae	*Oemleria cerasiformis*	lacking nitrogen-fixing symbiosis	AF288110	-	AF348551
Rosaceae	*Coleogyne ramosissima*	lacking nitrogen-fixing symbiosis	DQ851224	-	-
Rosaceae	*Kerria japonica*	lacking nitrogen-fixing symbiosis	AB073686	DQ250749	-
Rosaceae	*Polylepis tarapacana*	lacking nitrogen-fixing symbiosis	EU914268	-	-
Rosaceae	*Cliffortia graminea*	lacking nitrogen-fixing symbiosis	EU914279	-	AY634722
Rosaceae	*Acaena latebrosa*	lacking nitrogen-fixing symbiosis	EU914270	-	AY634702
Rosaceae	*Margyricarpus pinnatus*	lacking nitrogen-fixing symbiosis	-	-	AY634730
Rosaceae	*Marcetella moquiniana*	lacking nitrogen-fixing symbiosis	-	-	AY634729
Rosaceae	*Sarcopoterium spinosum*	lacking nitrogen-fixing symbiosis	-	-	AY634777
Rosaceae	*Bencomia caudata*	lacking nitrogen-fixing symbiosis	-	-	AY634719
Rosaceae	*Hagenia abyssinica*	lacking nitrogen-fixing symbiosis	-	-	AY634727
Rosaceae	*Leucosidea sericea*	lacking nitrogen-fixing symbiosis	EU914265	-	AY634728
Rosaceae	*Horkeliella purpurascens*	lacking nitrogen-fixing symbiosis	-	-	GQ384737
Rosaceae	*Ivesia saxosa*	lacking nitrogen-fixing symbiosis	FR851336	-	GQ384742
Rosaceae	*Horkelia tridentata*	lacking nitrogen-fixing symbiosis	FR851329	-	-
Rosaceae	*Tylosperma lignosa*	lacking nitrogen-fixing symbiosis	-	-	GQ384793
Rosaceae	*Piletophyllum micropetalum*	lacking nitrogen-fixing symbiosis	-	-	GQ384783
Rosaceae	*Sibbaldia semiglabra*	lacking nitrogen-fixing symbiosis	-	-	FJ422303
Rosaceae	*Sibbaldiopsis tridentata*	lacking nitrogen-fixing symbiosis	-	-	GQ245528
Rosaceae	*Lachemilla andina*	lacking nitrogen-fixing symbiosis	-	-	HM453979
Rosaceae	*Schistophyllidium bifurcum*	lacking nitrogen-fixing symbiosis	-	-	GQ384681
Rosaceae	*Sibbaldianthe adpressa*	lacking nitrogen-fixing symbiosis	-	-	FJ422304
Rosaceae	*Alchemilla glabra*	lacking nitrogen-fixing symbiosis	JN894582	JN891525	-
Rosaceae	*Aphanes arvensis*	lacking nitrogen-fixing symbiosis	JN894468	JN892207	-
Rosaceae	*Fragaria vesca*	lacking nitrogen-fixing symbiosis	AF288102	HM850009	AF348545
Rosaceae	*Chamaerhodos mongholica*	lacking nitrogen-fixing symbiosis	-	-	FJ422285
Rosaceae	*Drymocallis rupestris*	lacking nitrogen-fixing symbiosis	JN893882	JN892820	FJ422292
Surianaceae	*Cadellia pentastylis**	lacking nitrogen-fixing symbiosis	EU604056	L29491	-
Surianaceae	*Suriana maritima**	lacking nitrogen-fixing symbiosis	AY386950	U07680	-
Surianaceae	*Recchia mexicana*	lacking nitrogen-fixing symbiosis	EU604045	AM234270	-
Surianaceae	*Stylobasium spathulatum*	lacking nitrogen-fixing symbiosis	EU604032	U06828	-
Surianaceae	*Guilfoylia monostylis*	lacking nitrogen-fixing symbiosis	EU604031	L29494	-
Tetramelaceae	*Octomeles sumatrana**	lacking nitrogen-fixing symbiosis	AY968455	L21942	AY968574
Tetramelaceae	*Tetrameles nudiflora**	lacking nitrogen-fixing symbiosis	AY968458	AF206828	-
Ticodendraceae	*Ticodendron incognitum**	lacking nitrogen-fixing symbiosis	AB015463	AF061197	AY147073
Ulmaceae	*Ampelocera hottleyi**	lacking nitrogen-fixing symbiosis	-	AF500335	AF501592
Ulmaceae	*Hemiptelea davidii**	lacking nitrogen-fixing symbiosis	AF345322	AF500336	-

Ulmaceae	*Holoptelea integrifolia**	lacking nitrogen-fixing symbiosis	-	D86315	-
Ulmaceae	*Ulmus procera**	lacking nitrogen-fixing symbiosis	JN894246	HM850433	AF400593
Ulmaceae	*Zelkova serrata**	lacking nitrogen-fixing symbiosis	AB572557	AF206835	-
Urticaceae	*Cecropia obtusifolia**	lacking nitrogen-fixing symbiosis	GQ981958	GQ981694	DQ179377
Urticaceae	*Debregeasia saeneb**	lacking nitrogen-fixing symbiosis	JF317422	JF317481	-
Urticaceae	*Dendrocnide stimulans**	lacking nitrogen-fixing symbiosis	-	FJ432247	FJ432274
Urticaceae	*Elatostema parvum**	lacking nitrogen-fixing symbiosis	-	AY208703	AY208733
Urticaceae	*Laportea canadensis**	lacking nitrogen-fixing symbiosis	AY257537	AF500356	-
Urticaceae	*Lecanthus peduncularis**	lacking nitrogen-fixing symbiosis	-	-	DQ179370
Urticaceae	*Leucosyke capitellata**	lacking nitrogen-fixing symbiosis	-	FJ976144	FJ432258
Urticaceae	*Parietaria debilis**	lacking nitrogen-fixing symbiosis	HM851112	HM850235	FJ432256
Urticaceae	*Pilea cadierei**	lacking nitrogen-fixing symbiosis	JF317431	JF317491	DQ179359
Urticaceae	*Pourouma tomentosa**	lacking nitrogen-fixing symbiosis	FJ514760	FJ038203	FJ039338
Urticaceae	*Pouzolzia mixta**	lacking nitrogen-fixing symbiosis	JF270899	JF265556	-
Urticaceae	*Procris insularis**	lacking nitrogen-fixing symbiosis	-	AY208706	AY208729
Urticaceae	*Urera baccifera**	lacking nitrogen-fixing symbiosis	HM446752.2	GQ981911	-
Urticaceae	*Gesnouinia arborea*	lacking nitrogen-fixing symbiosis	-	-	DQ179372
Urticaceae	*Nothocnide repanda*	lacking nitrogen-fixing symbiosis	-	-	FJ432253
Urticaceae	*Cypholophus macrocephalus*	lacking nitrogen-fixing symbiosis	-	-	FJ432254
Urticaceae	*Forsskaolea sp.*	lacking nitrogen-fixing symbiosis	-	AM235162	-
Urticaceae	*Droguetia ambigua*	lacking nitrogen-fixing symbiosis	-	AM235161	-
Urticaceae	*Oreocnide rufescens*	lacking nitrogen-fixing symbiosis	-	JF738384	-
Urticaceae	*Boehmeria macrophylla var. scabrella*	lacking nitrogen-fixing symbiosis	JF317436	JF317496	-
Urticaceae	*Gonostegia hirta*	lacking nitrogen-fixing symbiosis	-	GQ436551	-
Urticaceae	*Pipturus argenteus*	lacking nitrogen-fixing symbiosis	-	JF738411	-
Urticaceae	*Coussapoa ovalifolia*	lacking nitrogen-fixing symbiosis	-	-	AF501616
Urticaceae	*Maoutia puya*	lacking nitrogen-fixing symbiosis	-	-	FJ432259
Urticaceae	*Poikilospermum sp.*	lacking nitrogen-fixing symbiosis	-	AF500362	AF501617
Urticaceae	*Pellionia daveauana*	lacking nitrogen-fixing symbiosis	-	AF500358	AF501612
Urticaceae	*Myriocarpa longipes*	lacking nitrogen-fixing symbiosis	-	AY208705	AY208724
Urticaceae	*Discocnide mexicana*	lacking nitrogen-fixing symbiosis	-	-	DQ179369
Urticaceae	*Didymodoxa caffra*	lacking nitrogen-fixing symbiosis	-	AM235160	-
Urticaceae	*Obetia tenax*	lacking nitrogen-fixing symbiosis	JF270874	JF265528	-
Urticaceae	*Hesperocnide tenella*	lacking nitrogen-fixing symbiosis	-	AF500355	-
Urticaceae	*Urtica dioica**	lacking nitrogen-fixing symbiosis	GU266610	AF500361	AY208725
Outgroups:					
Crossosomataceae	*Crossosoma bigelovii**	lacking nitrogen-fixing symbiosis	DQ443456	DQ307100	DQ307148
Larreoideae	*Guaiacum guatemalense**	lacking nitrogen-fixing symbiosis	DQ401366	Y15019	EU253461
Melianthaceae	*Melianthus villosus**	lacking nitrogen-fixing symbiosis	JQ479132	JQ479189	JQ581537
Oxalidaceae	*Oxalis latifolia**	lacking nitrogen-fixing symbiosis	EU437339	EU002282	JN639571
Ranunculaceae	*Ranunculus macranthus**	lacking nitrogen-fixing symbiosis	NC 008796	NC 008796	NC 008796
Lepidobotryaceae	*Ruptiliocarpon caracolito**	lacking nitrogen-fixing symbiosis	AY935918	AJ402997	EU328801

Table S4. Minimal age constraints used in PL analysis. Fossils from which ages were obtained (including reference), their stratigraphic position, and corresponding age in million years (Ma), are indicated.

Node	Source of age	Stratigraphic position	Age (Ma)	References
CG Cucurbitaceae	Seeds of Cucurbitaceae	Palaeocene	56	Collinson[8] Collinson et al.[9]
CG (*Anacaona* + *Penelopeia*)	Amber	Late Early Miocene	16	Iturralde-Vinent & MacPhee[10]
SG *Linnaeosicyos*	Pollen of *Hexacolpites echinatus*	Oligocene	23	Salard-Cheboldaeff[11] Muller[12]
CG Betulaceae	Flower and fruit of *Bedellia*	Santonian	83	Sims et al.[13]
CG Fagales	Normapolles of Fagales	Middle Cenomanian	96	Pacltová[14,15]
CG Juglandaceae	Flowers of *Budvaecarpus*	Cenomanian-Santonian	83.6	Knobloch & Mai[16]
SG *Cyclocarya*	*Cyclocarya brownii*	Paleocene	56	Crane et al.[17]
CG Casuarinaceae	*Gymnostoma antiquum*	Late Paleocene	56	Scriven & Hill[18]
CG Coryloideae	Pollens of *Corylus*	Maestrichtian	66	Muller[12]
SG Fabaceae	Fruits of Caesalpinioid	Paleocene	56	Herendeen & Crane[19]
SG *Machaerium*	Leaflets of *Machaerium*	Eocene	33.9	Herendeen & Dilcher[20]
SG *Tipuana*	Fruits of *Tipuana*	Middle to Late Miocene	5.3	Burnham[21]
SG *Robinia*	Woods of *Robinia*	Late Eocene	33.9	Berggren et al.[22]
SG *Cercis*	Leaf and fruit of *Cercis*	Late Eocene	33.9	MacGinite[23] Manchester[24]
SG *Hymenaea*	Flowers of *Hymenaea*	Late Eocene	33.9	Hueber & Langenheim[25], Poinar[26], Graham[27]
,SG *Arcoa*	Leaves of *Arcoa*	Late Eocene	33.9	MacGinitie[23]
SG *Mezoneuron*	Fruit of *Mezoneuron*	Middle Eocene	37.8	Herendeen & Zarucchi[28], Herendeen & Dilcher[29]
SG mimosoid	Fower of *Protomimosoidea buchananensis*	Paleocene-Eocene boundary	56	Crepet & Taylor[30,31]
SG Papilionoideae	Fowers of *Barnebyanthus buchananensis*	Late Paleocene	56	Crepet & Herendeen[32]
SG *Styphnolobium*	Fruit of *Styphnolobium*	Middle Eocene	37.8	Herendeen & Dilcher[20]
CG Rosaceae	Flowers of Rosaceae	Turonian	89.8	Crepet & Nixon[33]
SG *Prunus*	Fruits and leaves of *Prunus*	Middle Eocene	37.8	Cevallos-Ferrizand Stockey[34], Manchester[35]
SG *Amelanchier*	Leaves of *Amelanchier*	Middle Eocene	37.8	Wolfe & Wehr[36]
SG *Comptonia*	*Comptonia difforme*	Middle Eocene	40	Berry[37]
SG *Ulmus*	Fruits of *Ulmus*	Early Eocene	47.8	Manchester[38]
SG *Celtis*	Leaves and fruits of *Celtis*	Paleocene	56	Manchester et al.[39]
CG Ulmaceae	Leaves of Ulmaceae	Paleocene	56	Manchester[38]

Table S5 | Fossil constraints used in BEAST

Node	Minimum age (Ma)	Log（Mean）	Log（Stdev）
CG Cucurbitaceae	56	1.5	0.5
CG Betulaceae	83	1.5	0.5
CG Fagales	96	1.5	0.5
CG Juglandaceae	83.6	1.5	0.5
CG Casuarinaceae	56	1.5	0.5
CG Coryloideae	66	1.5	0.5
SG mimosoid	56	1.5	0.5
SG Papilionoideae	56	1.5	0.5
SG *Comptonia*	40	1.5	0.5
CG Rosaceae	89.8	1.5	0.5
SG *Ulmus*	47.8	1.5	0.5

Supplementary References:

1. Xiang, X.-G. et al. Large-scale phylogenetic analyses reveal fagalean diversification promoted by the interplay of diaspores and environments in the Paleogene. *Perspect. Plant Ecol.* **16**, 101–10 (2014).

2. Yokoyama, J., Suzuki, M., Iwatsuki, K. & Hasebe, M. Molecular phylogeny of *Coriaria*, with special emphasis on the disjunct distribution. *Mol. Phylogenet. Evol.* **14**, 11–19 (2000).

3. Zhang, L. B., Simmons, M. P. & Renner, S. S. A phylogeny of Anisophylleaceae based on six nuclear and plastid loci: ancient disjunctions and recent dispersal between South America, Africa, and Asia. *Mol. Phylogenet. Evol.* **44**, 1057–67 (2007).

4. Chin, S.-W., Shaw, J., Haberle, R., Wen, J. & Potter, D. Diversification of almonds, peaches, plums and cherries molecular systematics and biogeographic history of *Prunus* (Rosaceae). Mol. Phyl. Evol. **76**, 34-48 (2014).

5. Bell, C. D., Soltis, D. E. & Soltis, P. S. The age and diversification of the

angiosperms re-revisited. *Am. J. Bot.* **97**, 1296–1303 (2010).

6. Richardson, J. E., Chatrou, L. W., Mols, J. B., Erkens, R. H. J. & Pirie, M. D. Historical biogeography of two cosmopolitan families of flowering plants: Annonaceae and Rhamnaceae. *Phil. Trans. R. Soc. Lond. B* **359**, 1495-508 (2004).
7. Magallón, S., Gómez-Acevedo, S., Sánchez-Reyes L. L. & Hernández-Hernández, T. A metacalibrated time-tree documents the early rise of flowering plant phylogenetic diversity. *New Phytol.* in press (2015).
8. Collinson, M. E. The Felpham flora: a preliminary report. *Tertiary Res.* **8**, 29–32 (1986).
9. Collinson, M. E., Boulter, M. C. & Holmes, P. R. [Magnoliophyta (‘Angiospermae’)]. *The fossil record* [Benton M. J. (ed.)] [809–841] (Chapman and Hall, London, 1993).
10. Iturralde-Vinent, M. A. & MacPhee, R. D. E. Age and paleogeographical origin of Dominican amber. *Science* **273**, 1850–1852 (1996).
11. Salard-Cheboldaeff, M. Sur la palynoflore Maestrichtienne et Tertiaire du bassin sédimentaire littoral du Cameroun. *Pollen Spores* **20**, 215–260 (1978).
12. Muller, J. Significance of fossil pollen for angiosperm history. *Ann. Missouri Bot. Gard.* **71**, 419–443 (1984).
13. Sims, H. J., Herendeen, P. S., Lupia, R., Christopher, R. A. & Crane, P. R. Fossil flowers with Normapolles pollen from the Late Cretaceous of southeastern North America. *Rev. Palaeobot. Palyno.* **106**, 131–151 (1999).
14. Pacltová, B. Pollen grains of angiosperms in the Cenomanian Peruc Formation in

Bohemia. *Palaeobotanist* **15**, 52–54. (1966).

15. Pacltová, B. The evolution and distribution of Normapolles pollen during the Cenophytic. *Rev. Palaeobot. Palyno.* **35**, 175–208 (1981).

16. Knobloch, E. & Mai, D. H. Monographie der früchte und samen in der Kreide von Mitteleuropa. *Edice Rozpravy ústředniho ústavu Geologického* **47**, 1–219 (1986).

17. Crane, P. R., Manchester, S. R. & Dilcher, D. L. A preliminary survey of fossil leaves and well-preserved reproductive structures from the Sentinel Butte Formation (Paleocene) near Almont, North Dakota. *Field. Geol.* **20**, 1–63 (1990).

18. Scriven, L. J. & Hill, R. S. Macrofossil Casuarinaceae: their identification and the oldest macrofossil record, Gymnostoma antiquum sp. nov., from the late Paleocene of New South Wales, Australia. *Aust. Syst. Bot.* **8**, 1035–1053 (1995).

19. Herendeen, P. S. & Crane, P. R. [Early caesalpiniod fruits from the Palaeogene of southern England]. *Advances in legume systematics, vol. 4 The fossil record* [Herendeen, P. S. & Dilcher, D. L. (eds)] [57–68] (Royal Botanic Gardens, Kew, 1992).

20. Herendeen, P. S. & Dilcher, D. L. *Advances in legume systematics, part4. The fossil record*. (Royal Botanic Gardens, Kew, 1992)

21. Burnham, R. A new species of winged fruit from the Miocene of Ecuador: *Tipuana ecuatoriana* (Leguminosae). *Am. J. Bot.* **82**, 599–1607 (1995).

22. Berggren, W. A., Kent, D. V., Swisher III, C. C. & Aubry, M. P. [A revised Cenozoic geochronology and chronostratigraphy]. *Geochronology, Time Scales and Global Stratigraphic Correlation, Society for Sedimentary Geology Special*

Publication No. 54 [Berggren, W. A., Kent, D. V., Aubry, M. P. & Hardenbol, J. (eds)] [129–212] (Oklahoma: SEPM, Tulsa, 1995).

23. MacGinitie, H. D. Fossil plants of the Florissant Beds, Colorado. *Carnegie Inst. Washington Publ.* **599**, 1–198 (1953).

24. Manchester, S. R. Update on the megafossil flora of Florissant, Colorado. *Denver Mus. Nature Sci. Ser. 4* **1**, 137–161 (2001).

25. Hueber, F. M. & Langenheim, J. Dominican amber tree had African ancestors. *Geotimes* **31**, 8–10 (1986).

26. Poinar, Jr. G. O. *Hymenaea protera* sp.n. (Leguminosae, Caesalpinioideae) from Dominican amber has African affinities. *Experientia* **47**, 1075–1082 (1991).

27. Graham, A. [The current status of the legume fossil record in the Caribbean region]. *Advances in legume systematics, part4: the fossil record* [Herendeen, P. S. & Dilcher, D. L. (eds)] [161–167] (Royal Botanic Gardens, Kew, 1992).

28. Herendeen, P. S. & Zarucchi, J. L. Validation of *Caesalpinia* subgenus *Mezoneuron* (Desf.) Vidal and new combinations in *Caesalpinia* for two species of *Mezoneuron* from Africa. *Ann. Missouri Bot. Gard.* **77**, 54–855 (1990).

29. Herendeen, P. S. & Dilcher, D. L. *Caesalpinia* subgenus *Mezoneuron* (Leguminosae, Caesalpinioideae) from the Tertiary of North America. *Am. J. Bot.* **78**, 1–12 (1991).

30. Crepet, W. L. & Taylor, D. W. The diversification of the Leguminosae: First fossil evidence of the Mimosoideae and Papilionoideae. *Science* **288**, 1087–1089 (1985).

31. Crepet, W. L. & Taylor, D. W. Primitive mimosoid flowers from the

Paleocene-Eocene and their systematic and evolutionary implications. *Am. J. Bot.* **73**, 548–563 (1986).

32. Crepet, W. L. & Herendeen, P. S. [Papilionoid flowers from the early Eocene of southeastern North America]. *Advances in legume systematics, part4: the fossil record* [Herendeen, P. S. & Dilcher, D. L. (eds)] [43–55] (Royal Botanic Gardens, Kew, 1992).

33. Crepet, W. L. & Nixon, K. C. [The fossil history of stamens]. *The anther: form, function and phylogeny* [D'Arcy, W. G. & Keating, R. C. (eds)] [25–57] (Cambridge University Press, Cambridge, 1996).

34. Cevallos-Ferriz, S. R. S. & Stockey, R. A. Fruits and seeds from the Princeton chert (Middle Eocene) of British Columbia: Rosaceae (Prunoideae). *Bot. Gaz.* **152**, 369–379 (1991).

35. Manchester, S. R. Fruits and seeds of the Middle Eocene Nut Beds flora, Clarno Formation, North Central Oregon. *Palaeontogr. Am.* **58**, 1–205 (1994).

36. Wolfe, J. A. & Wehr, W. Rosaceous Chamaebatiaria-like foliage from the paleogene of western North America. *Aliso* **12**, 177–200 (1988).

37. Berry, E. W. Living and fossil species of *Comptonia*. *Am. Nat.* **40**, 485–524 (1906).

38. Manchester, S. R. Biogeographical relationships of North American Tertiary floras. *Ann. Missouri Bot. Gard.* **86**, 472–522 (1999).

39. Manchester, S. R., Akhmetiev, M. A. & Kodrul, T. Leaves and fruits of *Celtis aspera* (Newberry) comb. nov. (Celtidaceae) from the Paleocene of North America and eastern Asia. *Int. J. Plant Sci.* **163**, 725–736 (2002).

Fig. S1-A

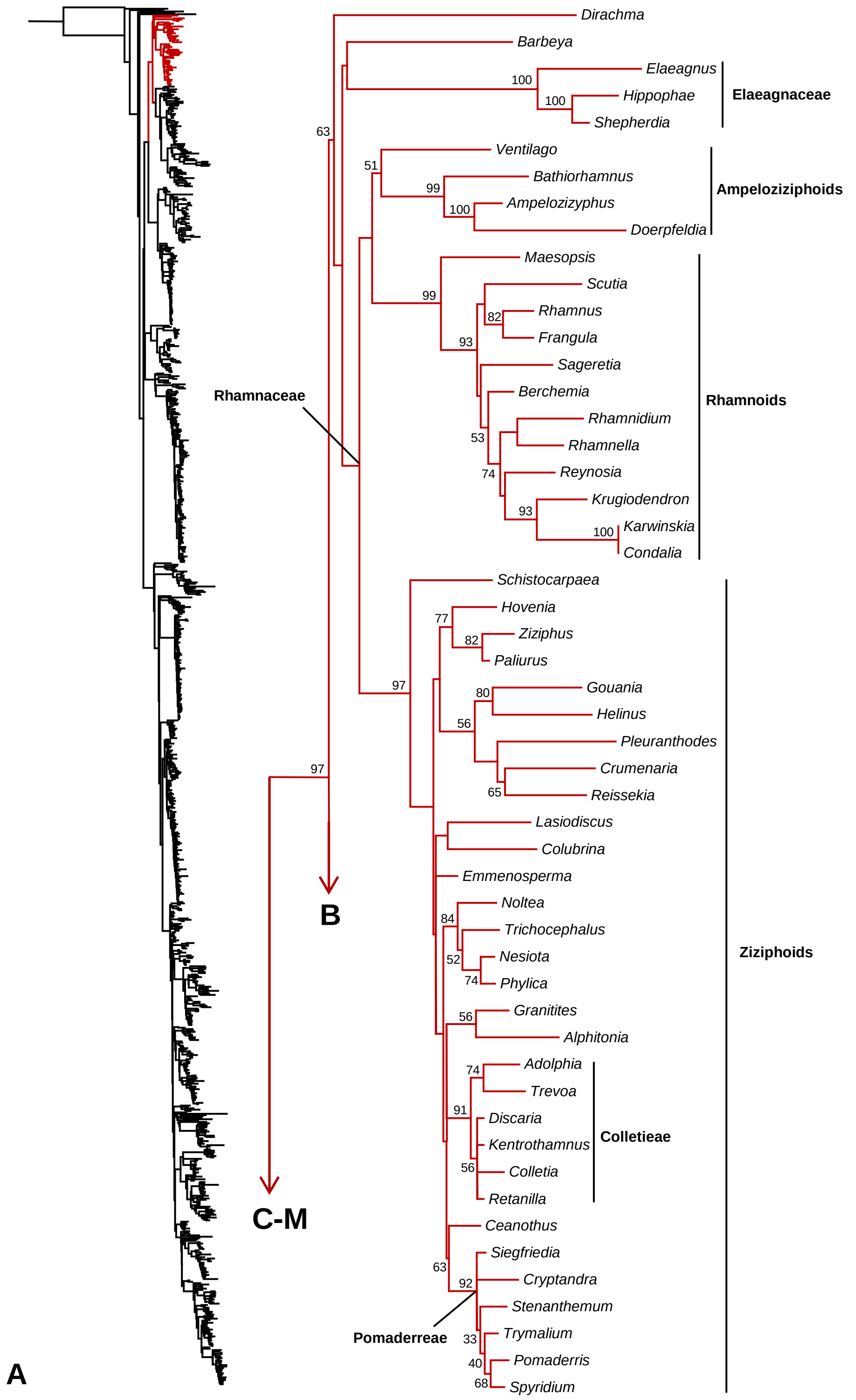

Dirachma
Barbeya
Elaeagnus
Hippophae
Shepherdia
Elaeagnaceae
Ventilago
Bathiorhamnus
Ampelozizyphus
Doerpfeldia
Ampeloziziphoids
Maesopsis
Scutia
Rhamnus
Frangula
Sageretia
Berchemia
Rhamnidium
Rhamnella
Reynosia
Krugiodendron
Karwinskia
Condalia
Rhamnoids
Rhamnaceae
Schistocarpaea
Hovenia
Ziziphus
Paliurus
Gouania
Helinus
Pleuranthodes
Crumenaria
Reissekia
Lasiodiscus
Colubrina
Emmenosperma
Noltea
Trichocephalus
Nesiota
Phylica
Granitites
Alphitonia
Adolphia
Trevoa
Discaria
Kentrothamnus
Colletia
Retanilla
Colletieae
Ceanothus
Siegfriedia
Cryptandra
Stenanthemum
Trymalium
Pomaderris
Spyridium
Pomaderreae
Ziziphoids
B
C-M
A

Fig. S1-B

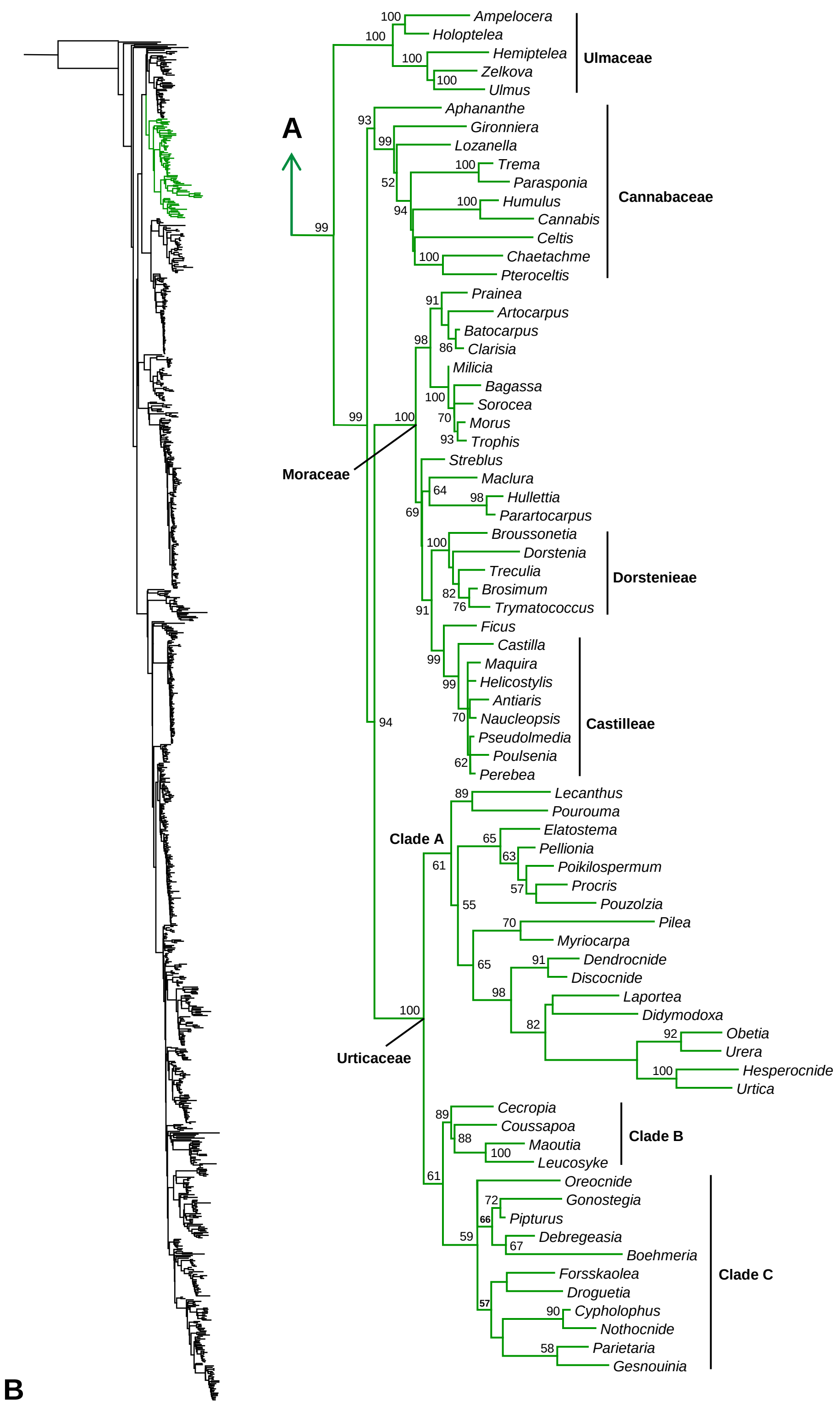

A
B
Ampelocera
Holoptelea
Hemiptelea
Zelkova
Ulmus
Ulmaceae
Aphananthe
Gironniera
Lozanella
Trema
Parasponia
Humulus
Cannabis
Celtis
Chaetachme
Pteroceltis
Cannabaceae
Prainea
Artocarpus
Batocarpus
Clarisia
Milicia
Bagassa
Sorocea
Morus
Trophis
Streblus
Maclura
Hullettia
Parartocarpus
Moraceae
Broussonetia
Dorstenia
Treculia
Brosimum
Trymatococcus
Dorstenieae
Ficus
Castilla
Maquira
Helicostylis
Antiaris
Naucleopsis
Pseudolmedia
Poulsenia
Perebea
Castilleae
Lecanthus
Pourouma
Clade A
Elatostema
Pellionia
Poikilospermum
Procris
Pouzolzia
Pilea
Myriocarpa
Dendrocnide
Discocnide
Laportea
Didymodoxa
Obetia
Urera
Hesperocnide
Urtica
Urticaceae
Cecropia
Coussapoa
Maoutia
Leucosyke
Clade B
Oreocnide
Gonostegia
Pipturus
Debregeasia
Boehmeria
Forsskaolea
Droguetia
Cypholophus
Nothocnide
Parietaria
Gesnouinia
Clade C

Fig. S1-C

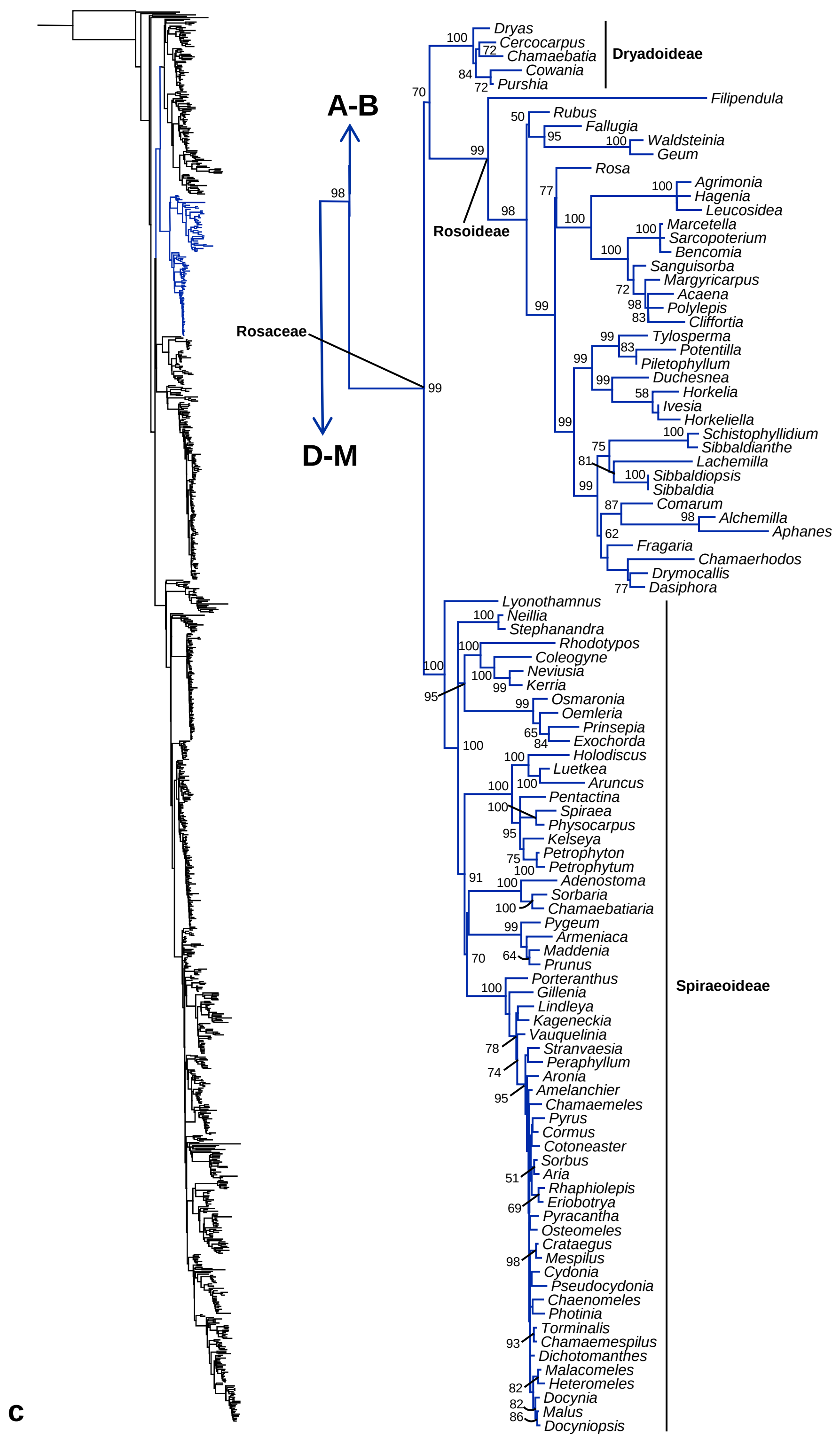
A-B
D-M
Rosaceae
Dryadoideae
Rosoideae
Spiraeoideae
Dryas
Cercocarpus
Chamaebatia
Cowania
Purshia
Filipendula
Rubus
Fallugia
Waldsteinia
Geum
Rosa
Agrimonia
Hagenia
Leucosidea
Marcetella
Sarcopoterium
Bencomia
Sanguisorba
Margyricarpus
Acaena
Polylepis
Cliffortia
Tylosperma
Potentilla
Piletophyllum
Duchesnea
Horkelia
Ivesia
Horkeliella
Schistophyllidium
Sibbaldianthe
Lachemilla
Sibbaldiopsis
Sibbaldia
Comarum
Alchemilla
Aphanes
Fragaria
Chamaerhodos
Drymocallis
Dasiphora
Lyonothamnus
Neillia
Stephanandra
Rhodotypos
Coleogyne
Neviusia
Kerria
Osmaronia
Oemleria
Prinsepia
Exochorda
Holodiscus
Luetkea
Aruncus
Pentactina
Spiraea
Physocarpus
Kelseya
Petrophyton
Petrophytum
Adenostoma
Sorbaria
Chamaebatiaria
Pygeum
Armeniaca
Maddenia
Prunus
Porteranthus
Gillenia
Lindleya
Kageneckia
Vauquelinia
Stranvaesia
Peraphyllum
Aronia
Amelanchier
Chamaemeles
Pyrus
Cormus
Cotoneaster
Sorbus
Aria
Rhaphiolepis
Eriobotrya
Pyracantha
Osteomeles
Crataegus
Mespilus
Cydonia
Pseudocydonia
Chaenomeles
Photinia
Torminalis
Chamaemespilus
Dichotomanthes
Malacomeles
Heteromeles
Docynia
Malus
Docyniopsis
c

Fig. S1-D

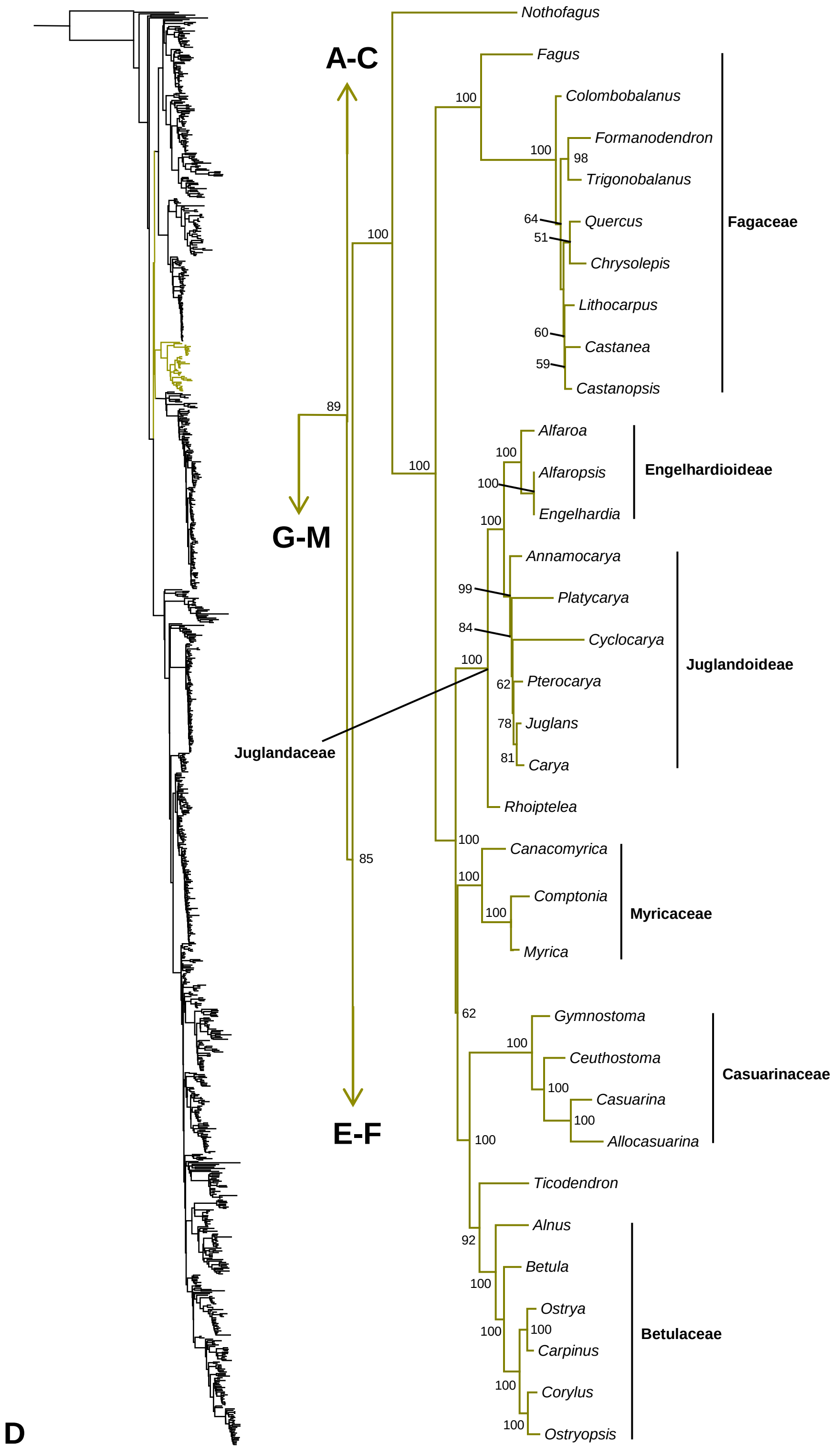

A-C
G-M
E-F
D
Nothofagus
Fagus
Colombobalanus
Formanodendron
Trigonobalanus
Quercus
Chrysolepis
Lithocarpus
Castanea
Castanopsis
Fagaceae
Alfaroa
Alfaropsis
Engelhardia
Engelhardioideae
Annamocarya
Platycarya
Cyclocarya
Pterocarya
Juglans
Carya
Juglandoideae
Juglandaceae
Rhoiptelea
Canacomyrica
Comptonia
Myrica
Myricaceae
Gymnostoma
Ceuthostoma
Casuarina
Allocasuarina
Casuarinaceae
Ticodendron
Alnus
Betula
Ostrya
Carpinus
Corylus
Ostryopsis
Betulaceae

Fig. S1-E

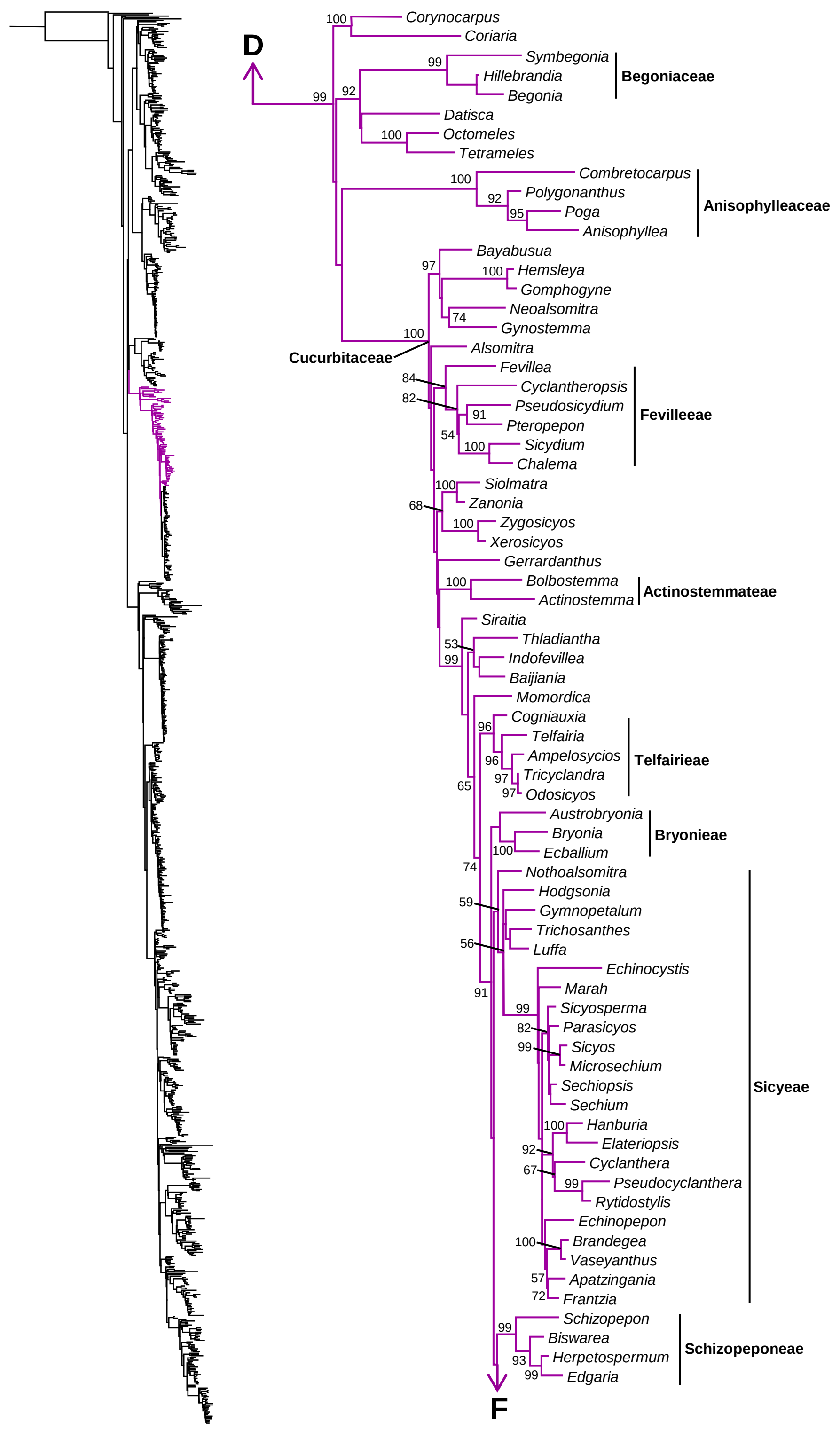
D
Corynocarpus
Coriaria
Symbegonia
Hillebrandia
Begonia
Begoniaceae
Datisca
Octomeles
Tetrameles
Combretocarpus
Polygonanthus
Poga
Anisophyllea
Anisophylleaceae
Bayabusua
Hemsleya
Gomphogyne
Neoalsomitra
Gynostemma
Cucurbitaceae
Alsomitra
Fevillea
Cyclantheropsis
Pseudosicydium
Pteropepon
Sicydium
Chalema
Fevilleeae
Siolmatra
Zanonia
Zygosicyos
Xerosicyos
Gerrardanthus
Bolbostemma
Actinostemma
Actinostemmateae
Siraitia
Thladiantha
Indofevillea
Baijiania
Momordica
Cogniauxia
Telfairia
Ampelosycios
Tricyclandra
Odosicyos
Telfairieae
Austrobryonia
Bryonia
Ecballium
Bryonieae
Nothoalsomitra
Hodgsonia
Gymnopetalum
Trichosanthes
Luffa
Echinocystis
Marah
Sicyosperma
Parasicyos
Sicyos
Microsechium
Sechiopsis
Sechium
Hanburia
Elateriopsis
Cyclanthera
Pseudocyclanthera
Rytidostylis
Echinopepon
Brandegea
Vaseyanthus
Apatzingania
Frantzia
Sicyeae
Schizopepon
Biswarea
Herpetospermum
Edgaria
Schizopeponeae
E
F

Fig. S1-F

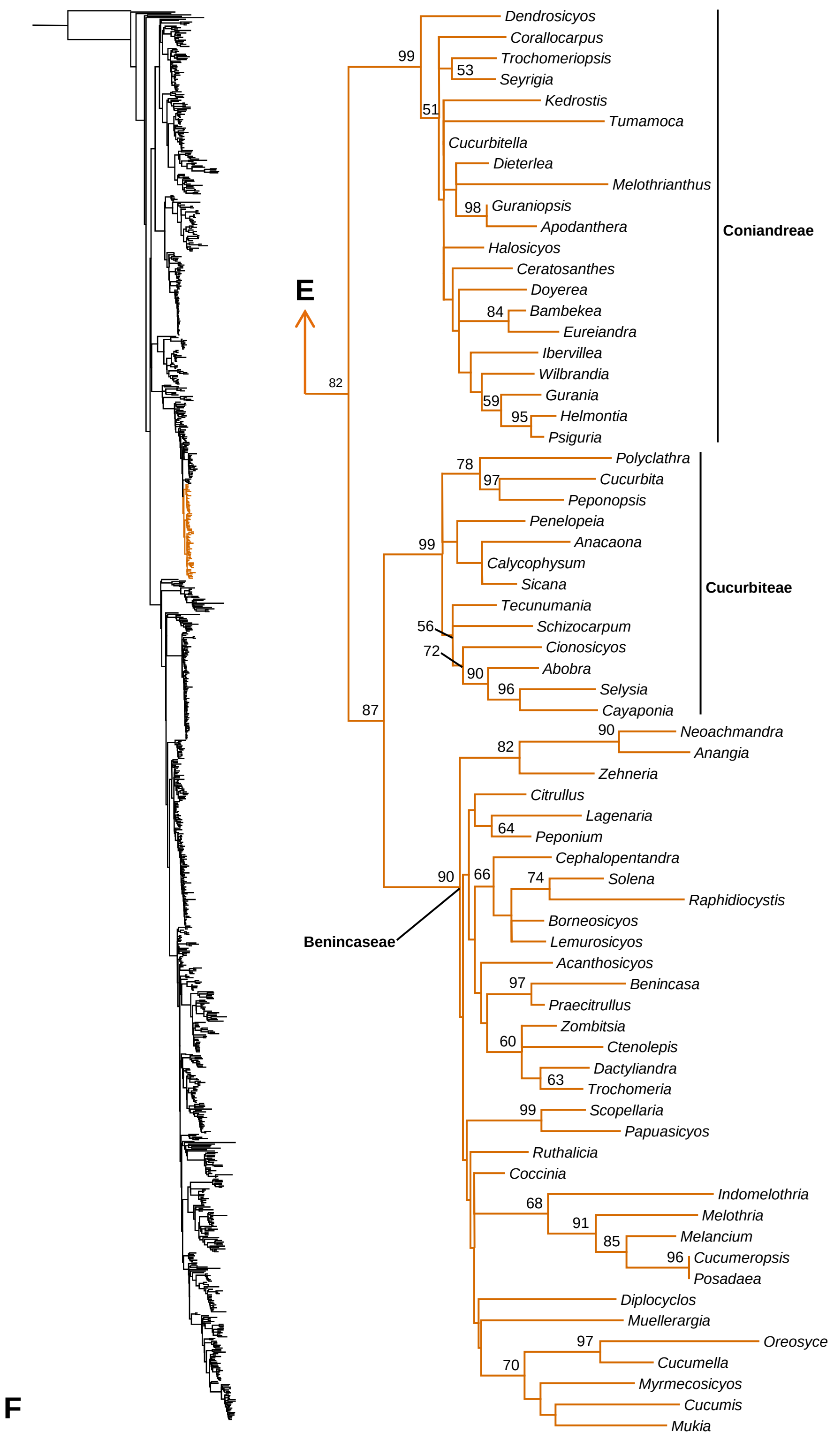
E
82
99
Dendrosicyos
Corallocarpus
53
Trochomeriopsis
Seyrigia
51
Kedrostis
Tumamoca
Cucurbitella
Dieterlea
Melothrianthus
98
Guraniopsis
Apodanthera
Halosicyos
Ceratosanthes
Doyerea
84
Bambekea
Eureiandra
Ibervillea
Wilbrandia
59
Gurania
95
Helmontia
Psiguria
Coniandreae
78
Polyclathra
97
Cucurbita
Peponopsis
99
Penelopeia
Anacaona
Calycophysum
Sicana
Tecunumania
56
Schizocarpum
72
Cionosicyos
90
Abobra
96
Selysia
Cayaponia
Cucurbiteae
87
90
Neoachmandra
82
Anangia
Zehneria
Citrullus
Lagenaria
64
Peponium
90
66
Cephalopentandra
74
Solena
Raphidiocystis
Borneosicyos
Lemurosicyos
Benincaseae
Acanthosicyos
97
Benincasa
Praecitrullus
60
Zombitsia
Ctenolepis
Dactyliandra
63
Trochomeria
99
Scopellaria
Papuasicyos
Ruthalicia
Coccinia
68
Indomelothria
91
Melothria
85
Melancium
96
Cucumeropsis
Posadaea
Diplocyclos
Muellerargia
97
Oreosyce
Cucumella
70
Myrmecosicyos
Cucumis
Mukia
F

Fig. S1-G

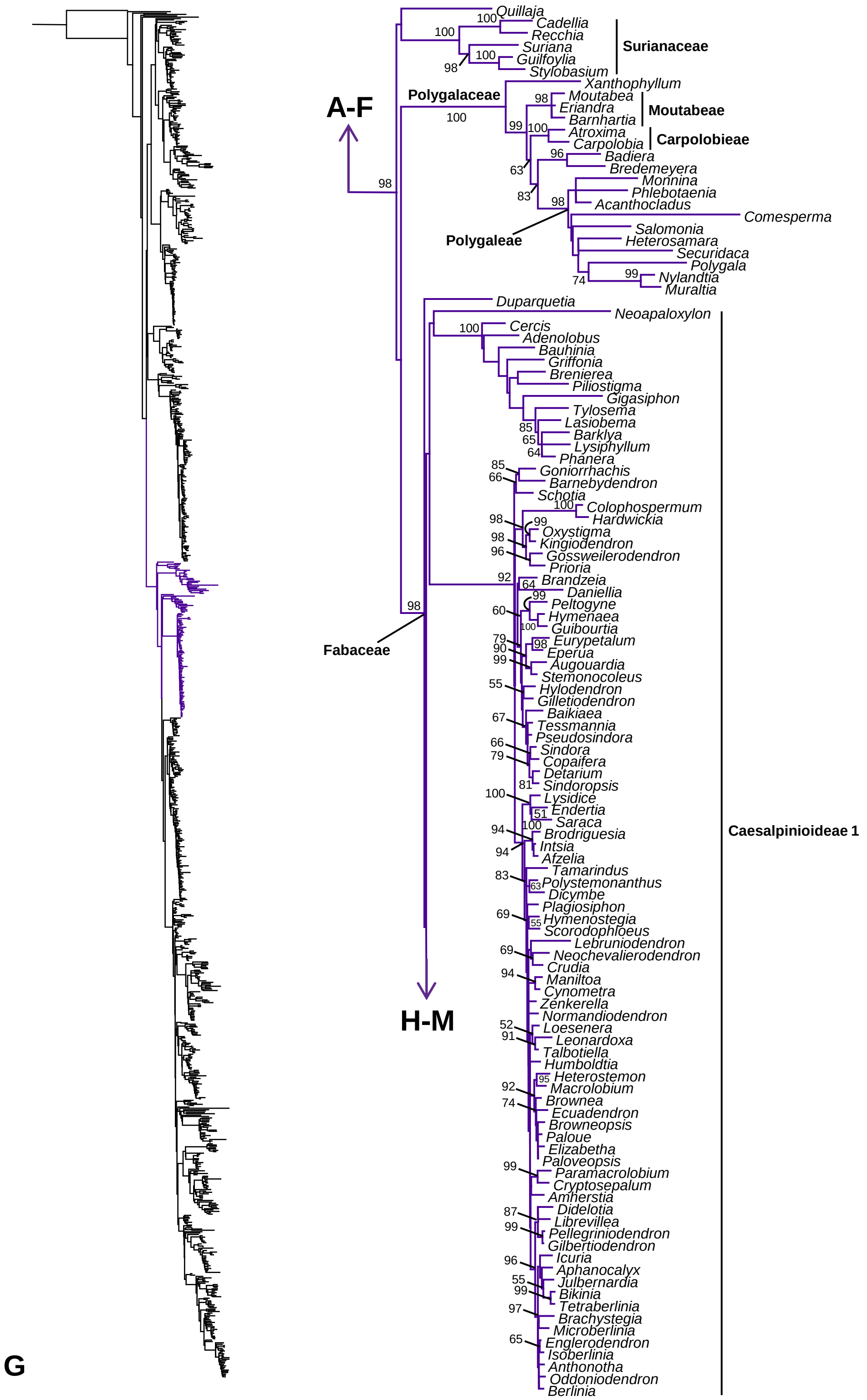

G
A-F
H-M
Quillaja
Cadellia
Recchia
Suriana
Guilfoylia
Stylobasium
Surianaceae
Polygalaceae
Xanthophyllum
Moutabea
Eriandra
Barnhartia
Moutabeae
Atroxima
Carpolobia
Carpolobieae
Badiera
Bredemeyera
Monnina
Phlebotaenia
Acanthocladus
Comesperma
Polygaleae
Salomonia
Heterosamara
Securidaca
Polygala
Nylandtia
Muraltia
Duparquetia
Neoapaloxylon
Cercis
Adenolobus
Bauhinia
Griffonia
Brenierea
Piliostigma
Gigasiphon
Tylosema
Lasiobema
Barklya
Lysiphyllum
Phanera
Goniorrhachis
Barnebydendron
Schotia
Colophospermum
Hardwickia
Oxystigma
Kingiodendron
Gossweilerodendron
Prioria
Brandzeia
Daniellia
Peltogyne
Hymenaea
Guibourtia
Eurypetalum
Eperua
Augouardia
Stemonocoleus
Hylodendron
Gilletiodendron
Baikiaea
Tessmannia
Pseudosindora
Sindora
Copaifera
Detarium
Sindoropsis
Lysidice
Endertia
Saraca
Brodriguesia
Intsia
Afzelia
Tamarindus
Polystemonanthus
Dicymbe
Plagiosiphon
Hymenostegia
Scorodophloeus
Lebruniodendron
Neochevalierodendron
Crudia
Maniltoa
Cynometra
Zenkerella
Normandiodendron
Loesenera
Leonardoxa
Talbotiella
Humboldtia
Heterostemon
Macrolobium
Brownea
Ecuadendron
Browneopsis
Paloue
Elizabetha
Paloveopsis
Paramacrolobium
Cryptosepalum
Amherstia
Didelotia
Librevillea
Pellegriniodendron
Gilbertiodendron
Icuria
Aphanocalyx
Julbernardia
Bikinia
Tetraberlinia
Brachystegia
Microberlinia
Englerodendron
Isoberlinia
Anthonotha
Oddoniodendron
Berlinia
Fabaceae
Caesalpinioideae 1

Fig. S1-H

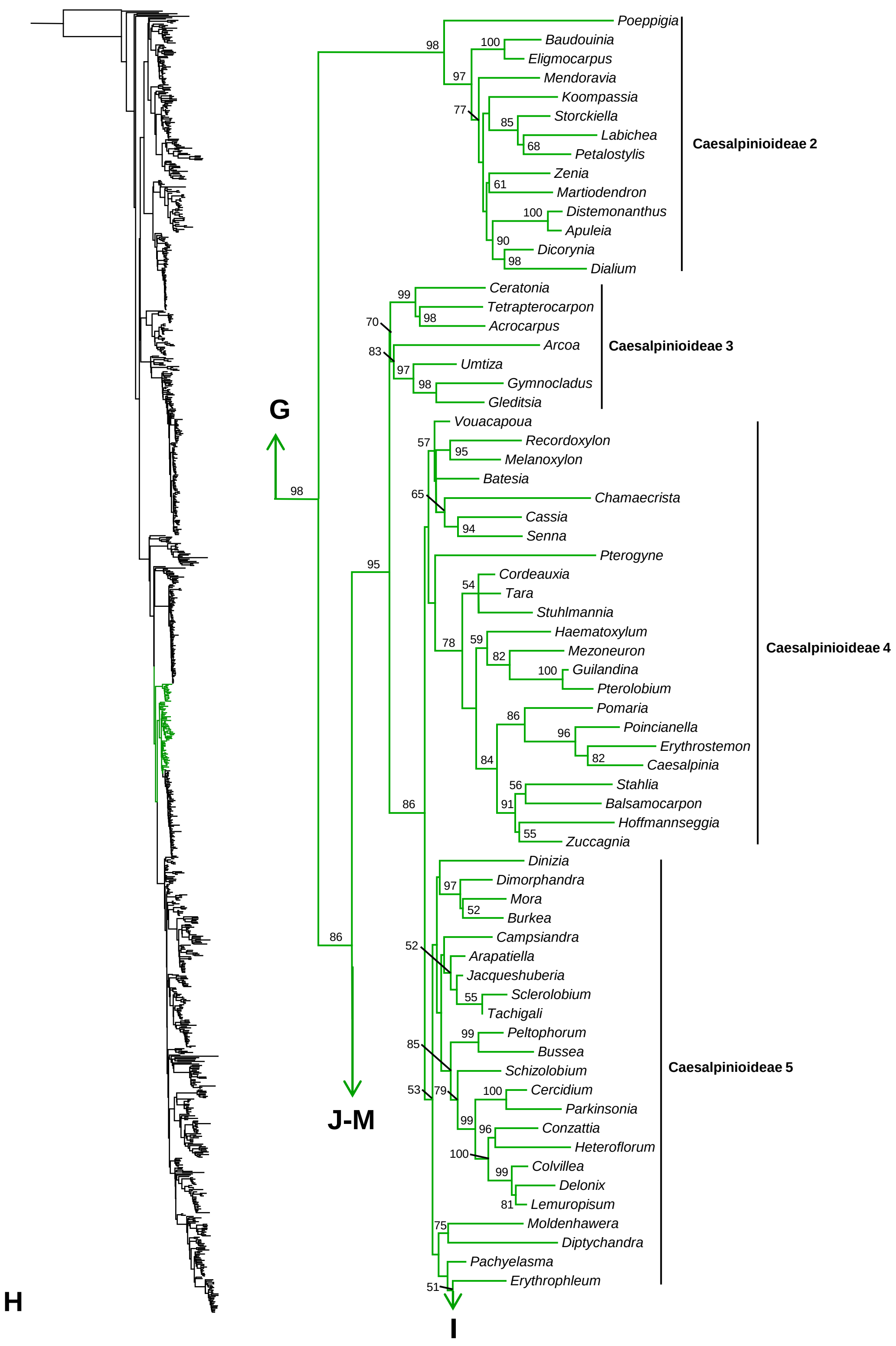
H
G
J-M
I
Poeppigia
Baudouinia
Eligmocarpus
Mendoravia
Koompassia
Storckiella
Labichea
Petalostylis
Zenia
Martiodendron
Distemonanthus
Apuleia
Dicorynia
Dialium
Caesalpinioideae 2
Ceratonia
Tetrapterocarpon
Acrocarpus
Arcoa
Umtiza
Gymnocladus
Gleditsia
Caesalpinioideae 3
Vouacapoua
Recordoxylon
Melanoxylon
Batesia
Chamaecrista
Cassia
Senna
Pterogyne
Cordeauxia
Tara
Stuhlmannia
Haematoxylum
Mezoneuron
Guilandina
Pterolobium
Pomaria
Poincianella
Erythrostemon
Caesalpinia
Stahlia
Balsamocarpon
Hoffmannseggia
Zuccagnia
Caesalpinioideae 4
Dinizia
Dimorphandra
Mora
Burkea
Campsiandra
Arapatiella
Jacqueshuberia
Sclerolobium
Tachigali
Peltophorum
Bussea
Schizolobium
Cercidium
Parkinsonia
Conzattia
Heteroflorum
Colvillea
Delonix
Lemuropisum
Moldenhawera
Diptychandra
Pachyelasma
Erythrophleum
Caesalpinioideae 5

Fig. S1-I

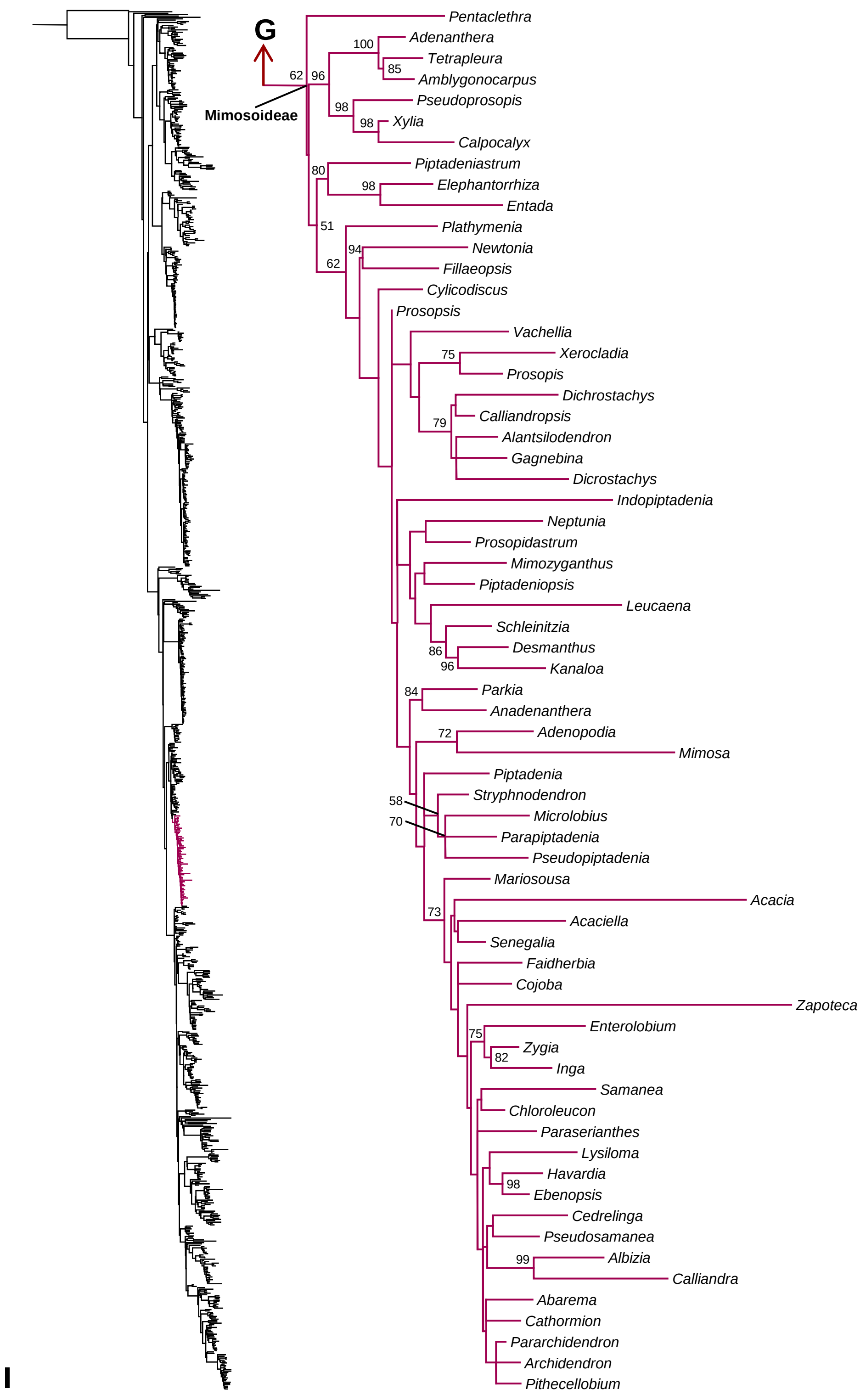
G
Mimosoideae
Pentaclethra
Adenanthera
Tetrapleura
Amblygonocarpus
Pseudoprosopis
Xylia
Calpocalyx
Piptadeniastrum
Elephantorrhiza
Entada
Plathymenia
Newtonia
Fillaeopsis
Cylicodiscus
Prosopsis
Vachellia
Xerocladia
Prosopis
Dichrostachys
Calliandropsis
Alantsilodendron
Gagnebina
Dicrostachys
Indopiptadenia
Neptunia
Prosopidastrum
Mimozyganthus
Piptadeniopsis
Leucaena
Schleinitzia
Desmanthus
Kanaloa
Parkia
Anadenanthera
Adenopodia
Mimosa
Piptadenia
Stryphnodendron
Microlobius
Parapiptadenia
Pseudopiptadenia
Mariosousa
Acacia
Acaciella
Senegalia
Faidherbia
Cojoba
Zapoteca
Enterolobium
Zygia
Inga
Samanea
Chloroleucon
Paraserianthes
Lysiloma
Havardia
Ebenopsis
Cedrelinga
Pseudosamanea
Albizia
Calliandra
Abarema
Cathormion
Pararchidendron
Archidendron
Pithecellobium
100
85
62
96
98
98
80
98
51
62
94
75
79
86
96
84
72
58
70
73
75
82
98
99

Fig. S1-J

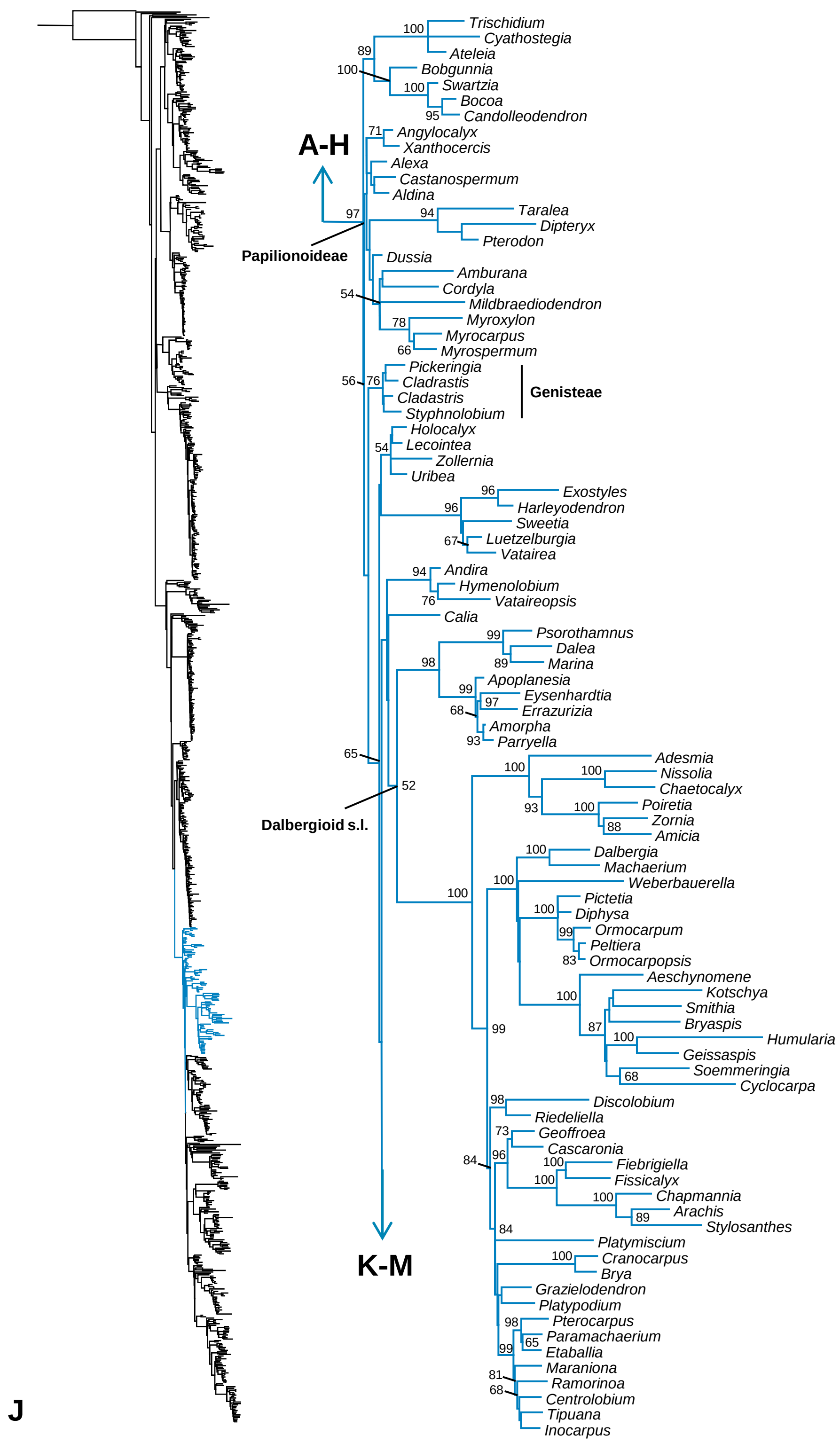

A-H
Papilionoideae
Genisteae
Dalbergioid s.l.
K-M
J
Trischidium
Cyathostegia
Ateleia
Bobgunnia
Swartzia
Bocoa
Candolleodendron
Angylocalyx
Xanthocercis
Alexa
Castanospermum
Aldina
Taralea
Dipteryx
Pterodon
Dussia
Amburana
Cordyla
Mildbraediodendron
Myroxylon
Myrocarpus
Myrospermum
Pickeringia
Cladrastis
Cladrastis
Styphnolobium
Holocalyx
Lecointea
Zollernia
Uribea
Exostyles
Harleyodendron
Sweetia
Luetzelburgia
Vatairea
Andira
Hymenolobium
Vataireopsis
Calia
Psorothamnus
Dalea
Marina
Apoplanesia
Eysenhardtia
Errazurizia
Amorpha
Parryella
Adesmia
Nissolia
Chaetocalyx
Poiretia
Zornia
Amicia
Dalbergia
Machaerium
Weberbauerella
Pictetia
Diphysa
Ormocarpum
Peltiera
Ormocarpopsis
Aeschynomene
Kotschya
Smithia
Bryaspis
Humularia
Geissaspis
Soemmeringia
Cyclocarpa
Discolobium
Riedeliella
Geoffroea
Cascaronia
Fiebrigiella
Fissicalyx
Chapmannia
Arachis
Stylosanthes
Platymiscium
Cranocarpus
Brya
Grazielodendron
Platypodium
Pterocarpus
Paramachaerium
Etaballia
Maraniona
Ramorinoa
Centrolobium
Tipuana
Inocarpus

Fig. S1-K

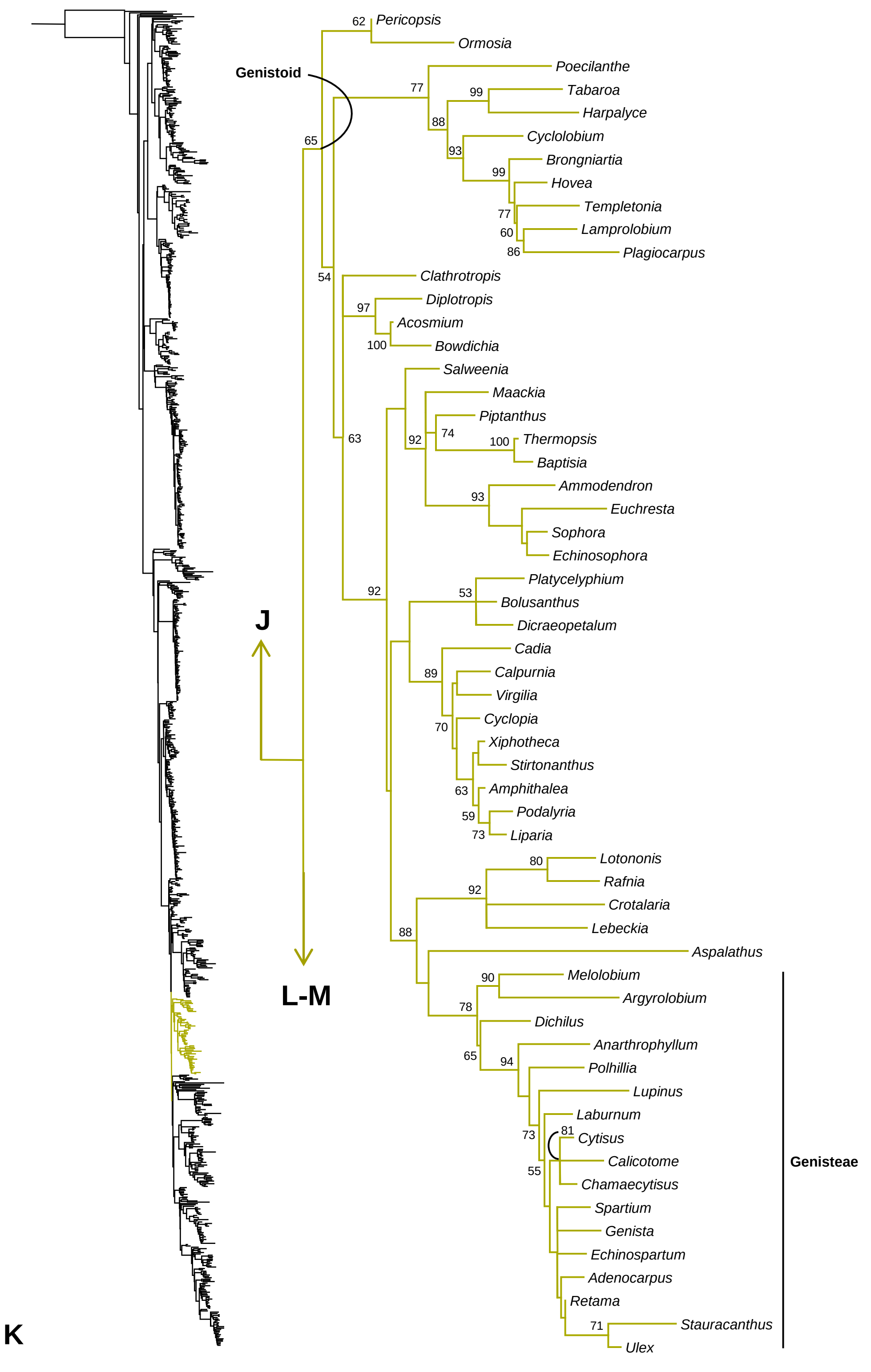
Genistoid
Pericopsis
Ormosia
Poecilanthe
Tabaroa
Harpalyce
Cyclolobium
Brongniartia
Hovea
Templetonia
Lamprolobium
Plagiocarpus
Clathrotropis
Diplotropis
Acosmium
Bowdichia
Salweenia
Maackia
Piptanthus
Thermopsis
Baptisia
Ammodendron
Euchresta
Sophora
Echinosophora
Platycelyphium
Bolusanthus
Dicraeopetalum
Cadia
Calpurnia
Virgilia
Cyclopia
Xiphotheca
Stirtonanthus
Amphithalea
Podalyria
Liparia
Lotononis
Rafnia
Crotalaria
Lebeckia
Aspalathus
Melolobium
Argyrolobium
Dichilus
Anarthrophyllum
Polhillia
Lupinus
Laburnum
Cytisus
Calicotome
Chamaecytisus
Spartium
Genista
Echinospartum
Adenocarpus
Retama
Stauracanthus
Ulex
Genisteae
J
L-M
K

Fig. S1-L

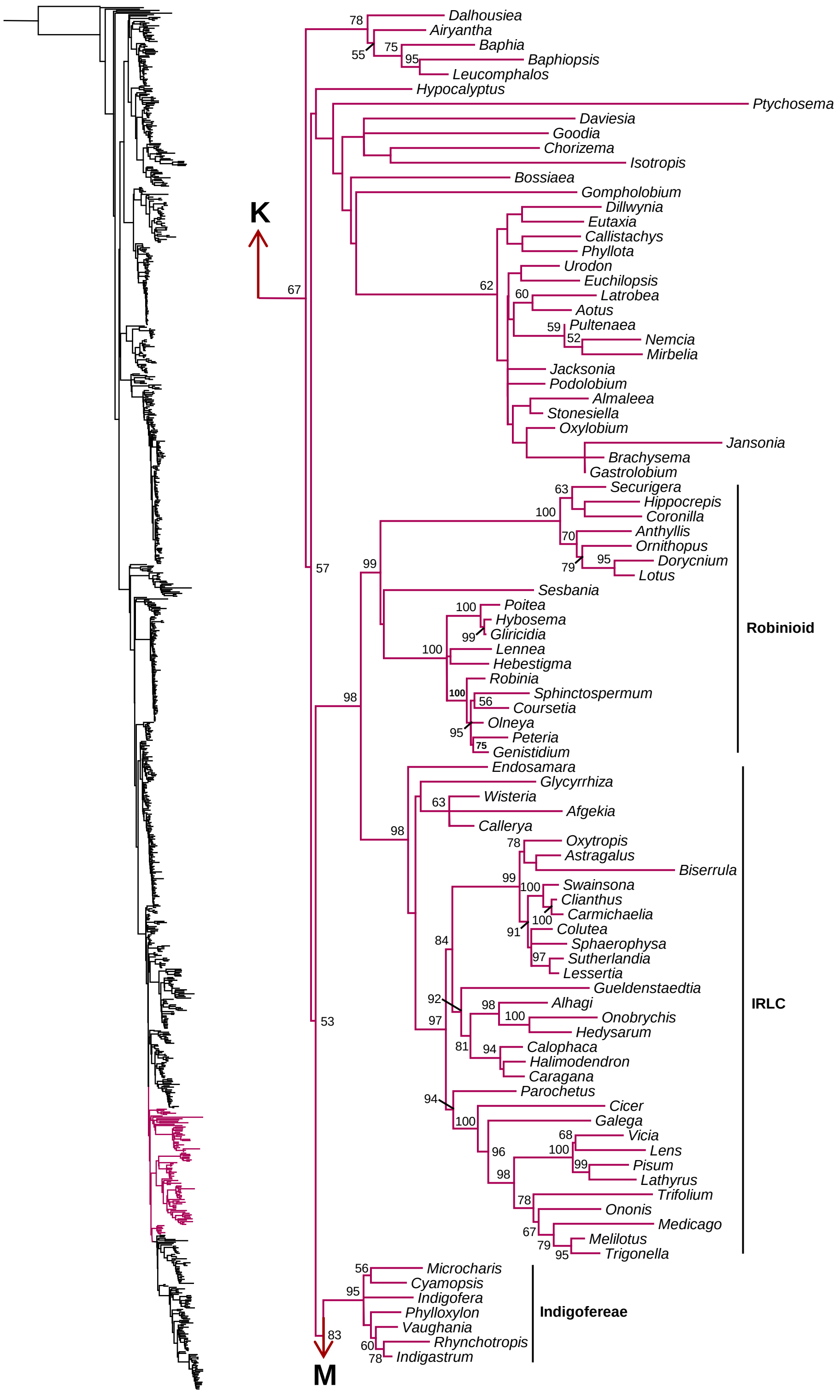

Dalhousiea
Airyantha
Baphia
Baphiopsis
Leucomphalos
Hypocalyptus
Ptychosema
Daviesia
Goodia
Chorizema
Isotropis
Bossiaea
Gompholobium
Dillwynia
Eutaxia
Callistachys
Phyllota
Urodon
Euchilopsis
Latrobea
Aotus
Pultenaea
Nemcia
Mirbelia
Jacksonia
Podolobium
Almaleea
Stonesiella
Oxylobium
Jansonia
Brachysema
Gastrolobium
Securigera
Hippocrepis
Coronilla
Anthyllis
Ornithopus
Dorycnium
Lotus
Sesbania
Poitea
Hybosema
Gliricidia
Lennea
Hebestigma
Robinia
Sphinctospermum
Coursetia
Olneya
Peteria
Genistidium
Robinioid
Endosamara
Glycyrrhiza
Wisteria
Afgekia
Callerya
Oxytropis
Astragalus
Biserrula
Swainsona
Clianthus
Carmichaelia
Colutea
Sphaerophysa
Sutherlandia
Lessertia
Gueldenstaedtia
Alhagi
Onobrychis
Hedysarum
Calophaca
Halimodendron
Caragana
Parochetus
Cicer
Galega
Vicia
Lens
Pisum
Lathyrus
Trifolium
Ononis
Medicago
Melilotus
Trigonella
IRLC
Microcharis
Cyamopsis
Indigofera
Phylloxylon
Vaughania
Rhynchotropis
Indigastrum
Indigofereae
K
M
L

Fig. S1-M

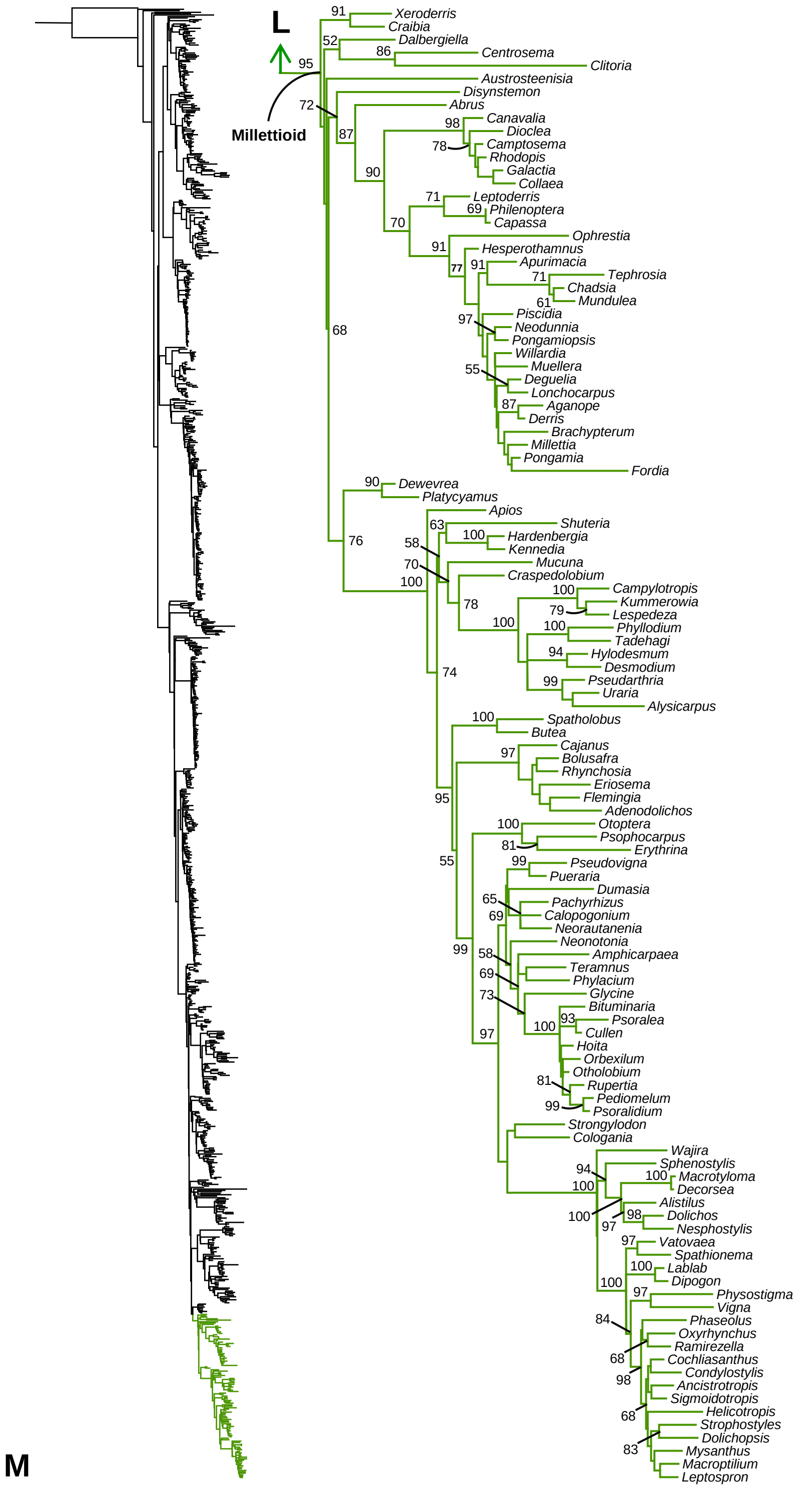
L
Millettioid
M
Xeroderris
Craibia
Dalbergiella
Centrosema
Clitoria
Austrosteenisia
Disynstemon
Abrus
Canavalia
Dioclea
Camptosema
Rhodopis
Galactia
Collaea
Leptoderris
Philenoptera
Capassa
Ophrestia
Hesperothamnus
Apurimacia
Tephrosia
Chadsia
Mundulea
Piscidia
Neodunnia
Pongamiopsis
Willardia
Muellera
Deguelia
Lonchocarpus
Aganope
Derris
Brachypterum
Millettia
Pongamia
Fordia
Dewevrea
Platycyamus
Apios
Shuteria
Hardenbergia
Kennedia
Mucuna
Craspedolobium
Campylotropis
Kummerowia
Lespedeza
Phyllodium
Tadehagi
Hylodesmum
Desmodium
Pseudarthria
Uraria
Alysicarpus
Spatholobus
Butea
Cajanus
Bolusafra
Rhynchosia
Eriosema
Flemingia
Adenodolichos
Otoptera
Psophocarpus
Erythrina
Pseudovigna
Pueraria
Dumasia
Pachyrhizus
Calopogonium
Neorautanenia
Neonotonia
Amphicarpaea
Teramnus
Phylacium
Glycine
Bituminaria
Psoralea
Cullen
Hoita
Orbexilum
Otholobium
Rupertia
Pediomelum
Psoralidium
Strongylodon
Cologania
Wajira
Sphenostylis
Macrotyloma
Decorsea
Alistilus
Dolichos
Nesphostylis
Vatovaea
Spathionema
Lablab
Dipogon
Physostigma
Vigna
Phaseolus
Oxyrhynchus
Ramirezella
Cochliasanthus
Condylostylis
Ancistrotropis
Sigmoidotropis
Helicotropis
Strophostyles
Dolichopsis
Mysanthus
Macroptilium
Leptospron

Fig. S2-1

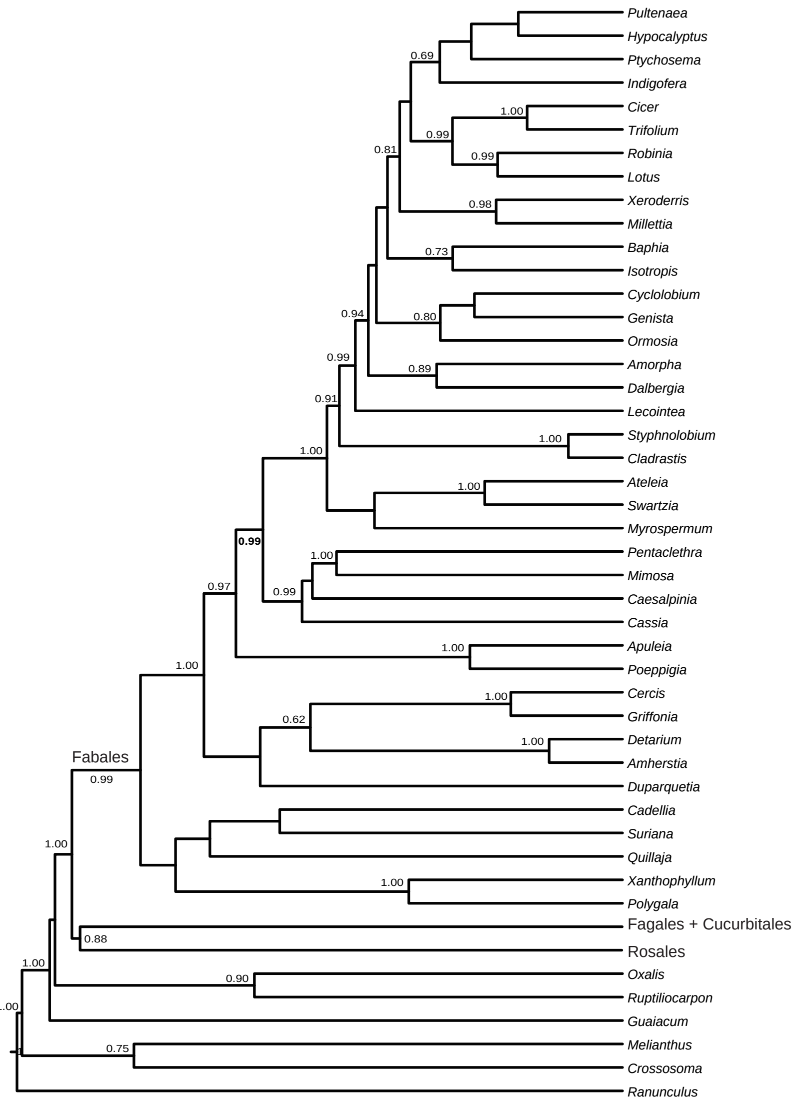

Pultenaea
Hypocalyptus
Ptychosema
Indigofera
Cicer
Trifolium
Robinia
Lotus
Xeroderris
Millettia
Baphia
Isotropis
Cyclolobium
Genista
Ormosia
Amorpha
Dalbergia
Lecointea
Styphnolobium
Cladrastis
Ateleia
Swartzia
Myrospermum
Pentaclethra
Mimosa
Caesalpinia
Cassia
Apuleia
Poeppigia
Cercis
Griffonia
Detarium
Amherstia
Duparquetia
Cadellia
Suriana
Quillaja
Xanthophyllum
Polygala
Fagales + Cucurbitales
Rosales
Oxalis
Ruptiliocarpon
Guaiacum
Melianthus
Crossosoma
Ranunculus
Fabales
0.69
1.00
0.99
0.99
0.81
0.98
0.73
0.94
0.80
0.99
0.89
0.91
1.00
1.00
1.00
0.99
1.00
0.97
0.99
1.00
1.00
1.00
0.62
1.00
0.99
1.00
1.00
0.88
1.00
0.90
1.00
0.75

Fig. S2-2

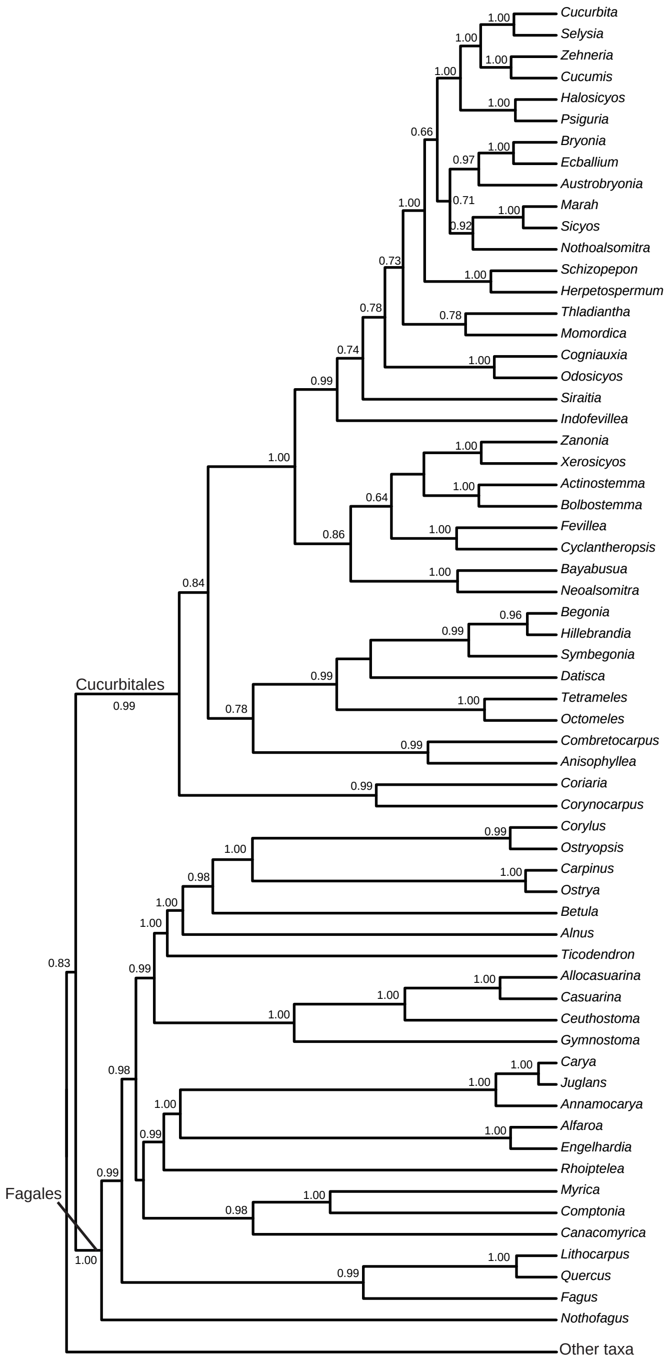
Cucurbita
Selysia
Zehneria
Cucumis
Halosicyos
Psiguria
Bryonia
Ecballium
Austrobryonia
Marah
Sicyos
Nothoalsomitra
Schizopepon
Herpetospermum
Thladiantha
Momordica
Cogniauxia
Odosicyos
Siraitia
Indofevillea
Zanonia
Xerosicyos
Actinostemma
Bolbostemma
Fevillea
Cyclantheropsis
Bayabusua
Neoalsomitra
Begonia
Hillebrandia
Symbegonia
Datisca
Tetrameles
Octomeles
Combretocarpus
Anisophyllea
Coriaria
Corynocarpus
Corylus
Ostryopsis
Carpinus
Ostrya
Betula
Alnus
Ticodendron
Allocasuarina
Casuarina
Ceuthostoma
Gymnostoma
Carya
Juglans
Annamocarya
Alfaroa
Engelhardia
Rhoiptelea
Myrica
Comptonia
Canacomyrica
Lithocarpus
Quercus
Fagus
Nothofagus
Other taxa
Cucurbitales
Fagales

Fig. S2-3

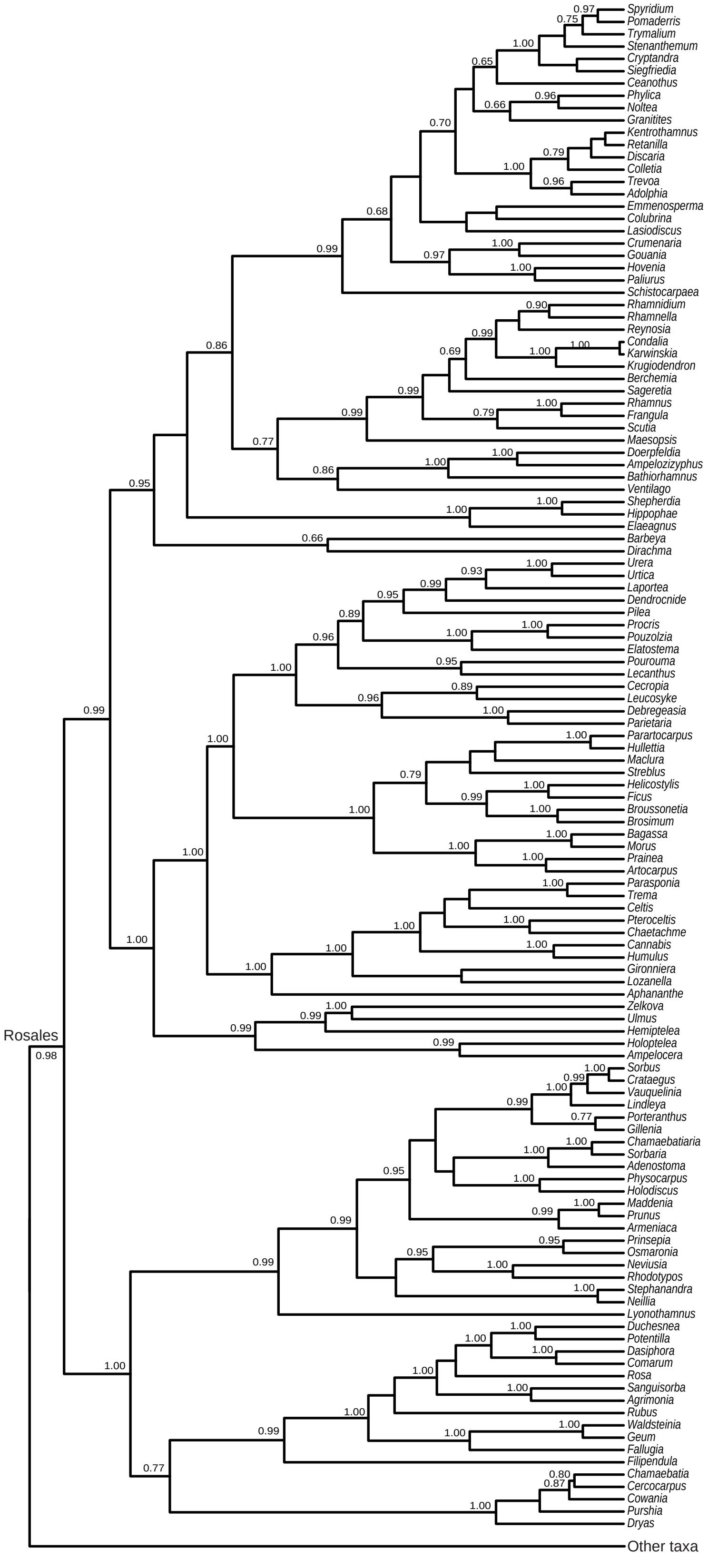
Spyridium
Pomaderris
Trymalium
Stenanthemum
Cryptandra
Siegfriedia
Ceanothus
Phylica
Noltea
Granitites
Kentrothamnus
Retanilla
Discaria
Colletia
Trevoa
Adolphia
Emmenosperma
Colubrina
Lasiodiscus
Crumenaria
Gouania
Hovenia
Paliurus
Schistocarpaea
Rhamnidium
Rhamnella
Reynosia
Condalia
Karwinskia
Krugiodendron
Berchemia
Sageretia
Rhamnus
Frangula
Scutia
Maesopsis
Doerpfeldia
Ampelozizyphus
Bathiorhamnus
Ventilago
Shepherdia
Hippophae
Elaeagnus
Barbeya
Dirachma
Urera
Urtica
Laportea
Dendrocnide
Pilea
Procris
Pouzolzia
Elatostema
Pourouma
Lecanthus
Cecropia
Leucosyke
Debregeasia
Parietaria
Parartocarpus
Hullettia
Maclura
Streblus
Helicostylis
Ficus
Broussonetia
Brosimum
Bagassa
Morus
Prainea
Artocarpus
Parasponia
Trema
Celtis
Pteroceltis
Chaetachme
Cannabis
Humulus
Gironniera
Lozanella
Aphananthe
Zelkova
Ulmus
Hemiptelea
Holoptelea
Ampelocera
Rosales
Sorbus
Crataegus
Vauquelinia
Lindleya
Porteranthus
Gillenia
Chamaebatiaria
Sorbaria
Adenostoma
Physocarpus
Holodiscus
Maddenia
Prunus
Armeniaca
Prinsepia
Osmaronia
Neviusia
Rhodotypos
Stephanandra
Neillia
Lyonothamnus
Duchesnea
Potentilla
Dasiphora
Comarum
Rosa
Sanguisorba
Agrimonia
Rubus
Waldsteinia
Geum
Fallugia
Filipendula
Chamaebatia
Cercocarpus
Cowania
Purshia
Dryas
Other taxa

Fig. S3-1

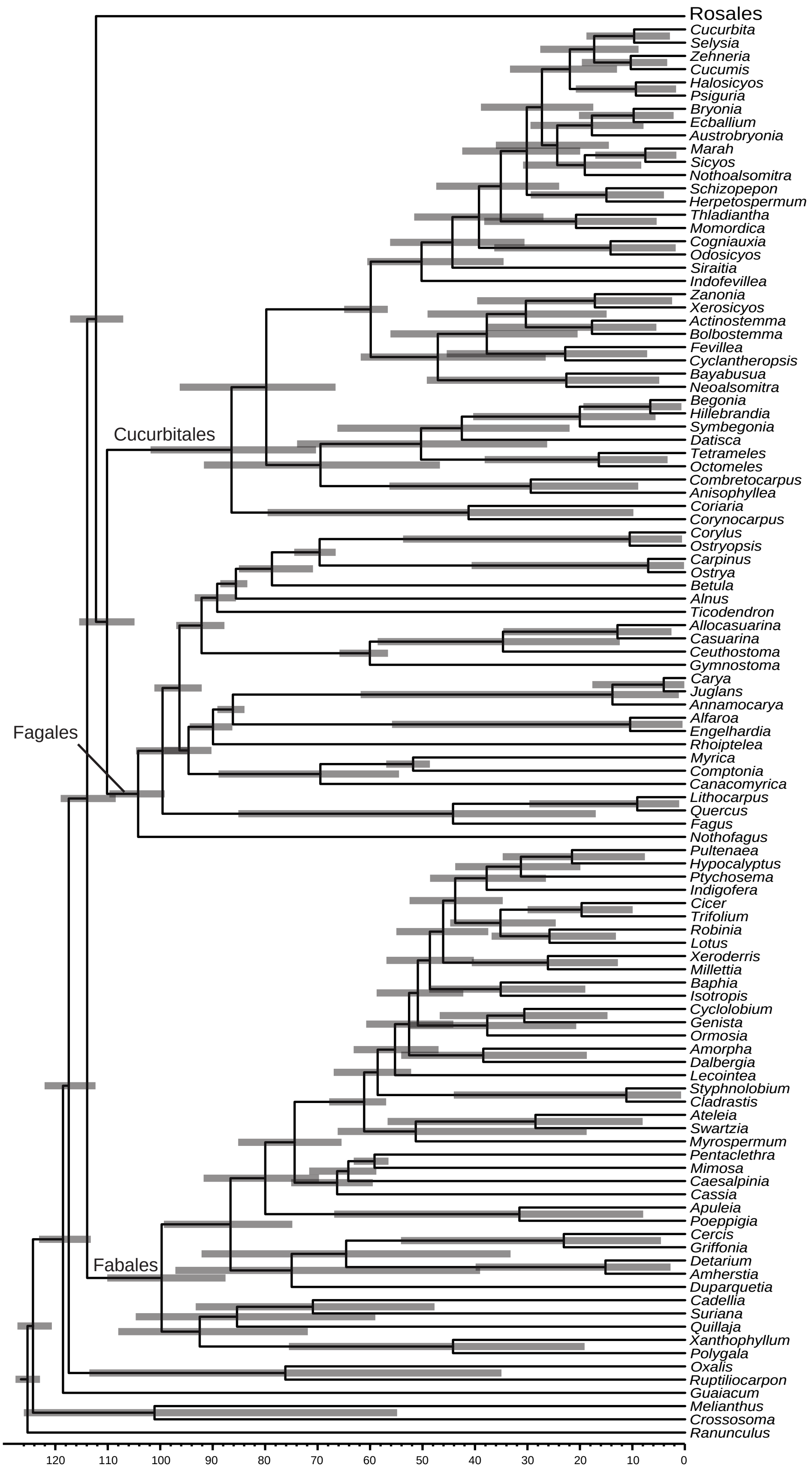
Rosales
Cucurbita
Selysia
Zehneria
Cucumis
Halosicyos
Psiguria
Bryonia
Ecballium
Austrobryonia
Marah
Sicyos
Nothoalsomitra
Schizopepon
Herpetospermum
Thladiantha
Momordica
Cogniauxia
Odosicyos
Siraitia
Indofevillea
Zanonia
Xerosicyos
Actinostemma
Bolbostemma
Fevillea
Cyclantheropsis
Bayabusua
Neoalsomitra
Begonia
Hillebrandia
Symbegonia
Datisca
Tetrameles
Octomeles
Combretocarpus
Anisophyllea
Coriaria
Corynocarpus
Corylus
Ostryopsis
Carpinus
Ostrya
Betula
Alnus
Ticodendron
Allocasuarina
Casuarina
Ceuthostoma
Gymnostoma
Carya
Juglans
Annamocarya
Alfaroa
Engelhardia
Rhoiptelea
Myrica
Comptonia
Canacomyrica
Lithocarpus
Quercus
Fagus
Nothofagus
Pultenaea
Hypocalyptus
Ptychosema
Indigofera
Cicer
Trifolium
Robinia
Lotus
Xeroderris
Millettia
Baphia
Isotropis
Cyclolobium
Genista
Ormosia
Amorpha
Dalbergia
Lecointea
Styphnolobium
Cladrastis
Ateleia
Swartzia
Myrospermum
Pentaclethra
Mimosa
Caesalpinia
Cassia
Apuleia
Poeppigia
Cercis
Griffonia
Detarium
Amherstia
Duparquetia
Cadellia
Suriana
Quillaja
Xanthophyllum
Polygala
Oxalis
Ruptiliocarpon
Guaiacum
Melianthus
Crossosoma
Ranunculus
Cucurbitales
Fagales
Fabales
120
110
100
90
80
70
60
50
40
30
20
10
0

Fig. S3-2

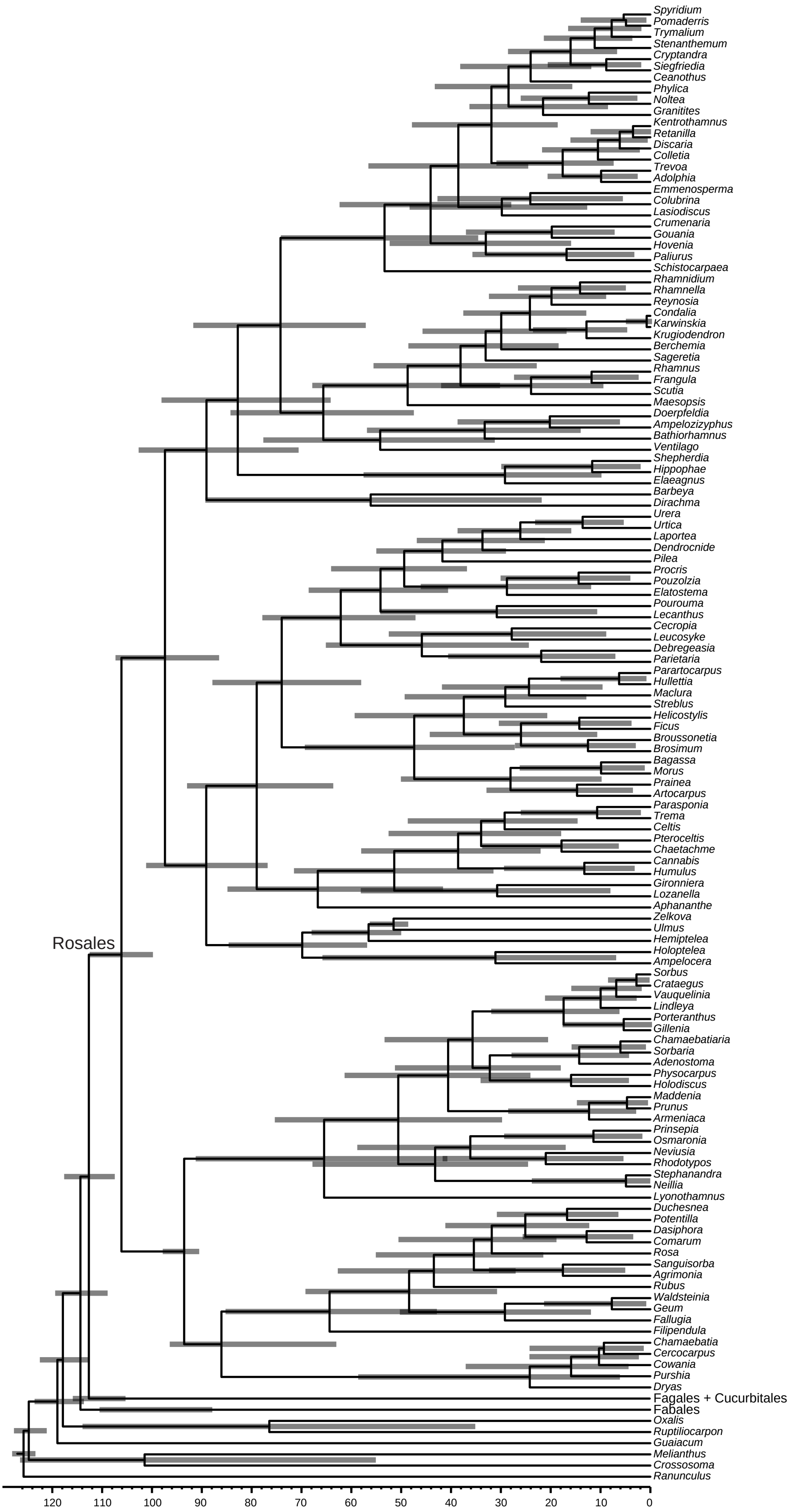
Spyridium
Pomaderris
Trymalium
Stenanthemum
Cryptandra
Siegfriedia
Ceanothus
Phylica
Noltea
Granitites
Kentrothamnus
Retanilla
Discaria
Colletia
Trevoa
Adolphia
Emmenosperma
Colubrina
Lasiodiscus
Crumenaria
Gouania
Hovenia
Paliurus
Schistocarpaea
Rhamnidium
Rhamnella
Reynosia
Condalia
Karwinskia
Krugiodendron
Berchemia
Sageretia
Rhamnus
Frangula
Scutia
Maesopsis
Doerpfeldia
Ampelozizyphus
Bathiorhamnus
Ventilago
Shepherdia
Hippophae
Elaeagnus
Barbeya
Dirachma
Urera
Urtica
Laportea
Dendrocnide
Pilea
Procris
Pouzolzia
Elatostema
Pourouma
Lecanthus
Cecropia
Leucosyke
Debregeasia
Parietaria
Parartocarpus
Hullettia
Maclura
Streblus
Helicostylis
Ficus
Broussonetia
Brosimum
Bagassa
Morus
Prainea
Artocarpus
Parasponia
Trema
Celtis
Pteroceltis
Chaetachme
Cannabis
Humulus
Gironniera
Lozanella
Aphananthe
Zelkova
Ulmus
Hemiptelea
Holoptelea
Ampelocera
Sorbus
Crataegus
Vauquelinia
Lindleya
Porteranthus
Gillenia
Chamaebatiaria
Sorbaria
Adenostoma
Physocarpus
Holodiscus
Maddenia
Prunus
Armeniaca
Prinsepia
Osmaronia
Neviusia
Rhodotypos
Stephanandra
Neillia
Lyonothamnus
Duchesnea
Potentilla
Dasiphora
Comarum
Rosa
Sanguisorba
Agrimonia
Rubus
Waldsteinia
Geum
Fallugia
Filipendula
Chamaebatia
Cercocarpus
Cowania
Purshia
Dryas
Fagales + Cucurbitales
Fabales
Oxalis
Ruptiliocarpon
Guaiacum
Melianthus
Crossosoma
Ranunculus
Rosales
120 110 100 90 80 70 60 50 40 30 20 10 0
